# The MsSCL28.5–MdMYB87 module enhances drought tolerance by promoting adventitious root development in apple rootstocks

**DOI:** 10.1093/hr/uhag211

**Published:** 2026-06-02

**Authors:** Maihemuti Turupu, Bingyang Du, Xiang Zhang, Zhengyang Wen, Haoyu Wang, Shuo Wang, Yaxin Chen, Feiyue Quan, Changhao Ru, Dingliang Zhang, Tianhong Li

**Affiliations:** Frontiers Science Center for Molecular Design Breeding, College of Horticulture, China Agricultural University, Beijing 100193, China; Frontiers Science Center for Molecular Design Breeding, College of Horticulture, China Agricultural University, Beijing 100193, China; Institute of Fruit and Tea Research, Qingdao Academy of Agricultural Sciences, Qingdao, Shandong 266000, China; Frontiers Science Center for Molecular Design Breeding, College of Horticulture, China Agricultural University, Beijing 100193, China; Frontiers Science Center for Molecular Design Breeding, College of Horticulture, China Agricultural University, Beijing 100193, China; Frontiers Science Center for Molecular Design Breeding, College of Horticulture, China Agricultural University, Beijing 100193, China; Frontiers Science Center for Molecular Design Breeding, College of Horticulture, China Agricultural University, Beijing 100193, China; Frontiers Science Center for Molecular Design Breeding, College of Horticulture, China Agricultural University, Beijing 100193, China; Frontiers Science Center for Molecular Design Breeding, College of Horticulture, China Agricultural University, Beijing 100193, China; Frontiers Science Center for Molecular Design Breeding, College of Horticulture, China Agricultural University, Beijing 100193, China; Frontiers Science Center for Molecular Design Breeding, College of Horticulture, China Agricultural University, Beijing 100193, China

## Abstract

Drought stress severely limits apple cultivation. *Malus sieversii*, a wild ancestor of domesticated apple, provides a valuable genetic resource for improving drought tolerance in cultivated apple rootstocks. Although plant-specific GRAS [GIBBERELLIC-ACID INSENSITIVE (GAI), REPRESSOR of GAI, and SCARECROW] transcription factors (TFs) are known to regulate development and stress responses, their precise functions and mechanisms in apple remain largely unexplored. Here, we demonstrated that overexpression of *MsSCL28.5*, a GRAS TF from *M. sieversii*, substantially enhances drought tolerance of rootstock ‘M26’ (*Malus domestica*). Transgenic plants exhibited increased adventitious root (AR) growth and improved reactive oxygen species scavenging capacity. MsSCL28.5 directly binds to the promoters of *MdLBD16a* and *MdLBD21b*, key genes regulating AR growth. We further identified the TF MdMYB87 as an interacting partner of MsSCL28.5. The resulting MsSCL28.5–MdMYB87 complex synergistically activates the transcription of *MdLBD16a*/*21b*. Additionally, grafting experiments confirmed that *MsSCL28.5*-overexpressing rootstocks systemically enhance the drought resistance of non-transgenic scions. Furthermore, we discovered that comprising reduced methylation of the *SCL28.5* promoter coupled with a TATA-box insertion in the *MYB87* promoter collectively contributes to the superior drought tolerance observed in *M. sieversii*. Our findings present molecular insights into how transcriptional regulation of SCL28.5-MYB87 module are integrated into drought response of different apple rootstocks.

## Introduction

Drought is one of the main abiotic stresses that impairs crop growth, reduces yield and quality, and ultimately threatens global food security [1, 2]. Apple (*Malus domestica* Borkh.) is a major economic fruit crop cultivated worldwide. In China, key apple production regions are predominantly located in arid and semiarid areas, where persistent drought limits the development of high-quality, sustainable apple industry [3]. Wild apple (*Malus sieversii*), the ancestor of modern domesticated apple cultivars, possesses a wide range of characterized resistance traits to both abiotic and biotic stresses. This makes it a valuable genetic resource for breeding and a promising source of drought-resistant rootstocks [4, 5]. However, the direct use of vigorous wild apple as a rootstock is often impractical for modern high-density orchards. Instead, dwarfing and semidwarfing rootstocks, such as the widely adopted ‘M26’ (*M. domestica*), are preferred. A key agronomic shortcoming of ‘M26’ is their shallow root system which restricts their application in arid areas. The contrasting drought tolerance of *M. sieversii* and ‘M26’ are rooted in distinct physiological traits. Under drought stress, *M. sieversii* maintains a significantly higher root to shoot ratio (R/S ratio) and greater root hydraulic conductivity than drought-sensitive rootstocks, enabling more efficient water uptake and transport [6]. It also shows elevated activities of superoxide dismutase (SOD) and peroxidase (POD), along with higher abscisic acid accumulation, which collectively mitigate drought-induced oxidative damage [6]. In contrast, ‘M26’ exhibits limited root biomass production under drought stress, and shows little stomatal closure even under severe water deficit [7]. Consistently, comparative studies have identified ‘M26’ as the most drought-susceptible genotype among common rootstocks, exhibiting the greatest reduction in photosynthetic performance under drought stress [8]. These well-documented physiological differences highlight *M. sieversii* as a promising donor of drought-tolerance traits. Therefore, using modern molecular techniques to identify and transfer key drought-tolerance genes from resilient wild germplasm (*M. sieversii*) into elite dwarfing rootstocks is of great significance for the development of the apple industry.

Drought stress can cause a range of detrimental changes to the plants, such as accumulation of reactive oxygen species (ROS), chlorophyll degradation, leaf wilting, and [9, 10]. In response, plants activate the multifaceted adaptive programs encompassing physiological adjustments, such as enhancing antioxidant activity, and morphological changes, such as altering root architecture [11–14]. Crucially, under drought stress, the expression of a variety of transcription factors (TFs), such as MYB (v-myb avian myeloblastosis viral oncogene homolog), bHLH (basic helix–loop–helix), APETALA2/ethylene-responsive factor, GRAS [GIBBERELLIC-ACID INSENSITIVE (GAI), REPRESSOR of GAI, and SCARECROW], and basic leucine zipper, which regulate extensive transcriptional networks to promote stress adaptation [15–17]. In apple, several TFs have been implicated in this process. For instance, MsNAC022 enhances tolerance by activating proline synthesis and ROS-scavenging genes [18], MdbHLH160 alleviates oxidative damage by upregulating *MdSOD1* [19], and MsDREB6.2 promotes root development [20]. Among the diverse TF families, the plant-specific GRAS proteins play critical roles in multiple biological processes, including organ development and gibberellin (GA) signal transduction [21]. They often function within protein complexes and are classified into subfamilies, such as DELLA, SHR, and SCL [22, 23]. Notably, GRAS TFs also contribute to stress adaptation, for instance, *PeSCL7* from poplar (*Populus* L.) [24], *AtSCL14* from Arabidopsis (*Arabidopsis thaliana*) [25], and *OsGRAS23* from rice (*Oryza sativa* L.) [26] enhance drought and oxidative stress tolerance. In transgenic tomato plants, overexpression (OE) of *SlGRAS24* resulted in longer roots and greater plant height [27]. The SCL28 subfamily, in particular, influences root growth by regulating cell division. Arabidopsis *scl28* mutants exhibit reduced root growth [28], while *RhSCL28* promotes petal size in rose (*Rosa hybrida*) by activating cell cycle genes [29]. To date, few GRAS TFs that regulate drought tolerance have been identified in apple, and the underlying mechanisms remains unknown.

Plant roots are essential multifunctional organs involved in the absorption of water and nutrients [30]. The molecular regulation of root architecture is a critical process for stress adaptation. In Arabidopsis, AUXIN RESPONSE FACTORs ARF7/ARF19 promote lateral root development by activating LATERAL ORGAN BOUNDARY DOMAIN (LBD) genes like *LBD16* and *LBD29* [31]. This module is conserved across diverse species and can be stress-responsive, as demonstrated by the salt-induced TF ZAT6, which enhances the salt tolerance in Arabidopsis by binding to the *LBD16* promoter to stimulate lateral root formation [32]. Similarly, in apple, specific TFs and microRNAs, including MsNAC022 and msi-miR156ab, have been shown to improve drought tolerance partly by promoting root growth [18, 33]. Given the paramount importance of roots in water acquisition, breeding rootstocks with superior root systems is a primary goal for sustainable apple cultivation. However, whether and how GRAS TFs are involved in LBD-mediated root growth in apple remains elusive.

The precise regulation of stress-responsive genes involves not only sequence-specific TFs but also dynamic epigenetic modifications, such as DNA methylation, which modulate transcriptional accessibility without altering the DNA sequence [34–36]. In plants, promoter methylation usually represses gene expression [37]. Its function extends beyond stress responses to fundamental developmental processes. For instance, global hypomethylation, often induced by DNA methyltransferase inhibitor 2′-deoxy-5-azacytidine (5-AZA), promotes various regeneration steps in plants, such as, callus development in peach [38] and shoot regeneration in soybean [39]. In apple, rejuvenation and adventitious rooting capacity are positively correlated with reduced DNA methylation levels, and 5-AZA treatment enhances the expression of rooting-related genes [40]. Despite this established link between methylation and developmental progress, the role of differential DNA methylation in shaping stress resilience traits between *M. domestica* and *M. sieversii* remains largely unexplored. Conventional breeding of apple is challenging due to its long generation life cycle. Molecular breeding offers a strategy to accelerate the development of new varieties with desirable traits [41, 42]. In practice, stress-resilient apple cultivation often relies on grafting a high-quality fruit-bearing scions onto a rootstock that provides robust stress tolerance. In our previous studies, we have investigated the biological functions of candidate genes in GL-3 or in the rootstock ‘M26’ itself [5, 18, 20, 33, 43]. In this work, we take a critical step toward application by validating the essential role of *MsSCL28.5*-OE plants as a rootstock. This approach demonstrates a direct pathway for translating genetically enhanced rootstocks into agricultural practice.

In our previous work, we identified the GRAS TF SCL28.5 as highly expressed in the roots of *M. sieversii* and responsive to drought stress [43]. As a member of the GRAS family, MsSCL28.5 is likely involved in multiple biological processes; however, its specific role and molecular mechanism in conferring drought tolerance to the cultivated apple rootstocks remained unexplored. In the current study, we reported that OE of *MsSCL28.5* significantly enhances drought tolerance of ‘M26’. MsSCL28.5 interacts with TF MdMYB87 to form a transcriptional complex that synergistically activates the expression of *MdLBD16a* and *MdLBD21b*, thereby promoting the adventitious root (AR) growth. Grafting experiments confirm that *MsSCL28.5*-OE rootstocks systemically enhance the drought resistance of wild-type scions. Furthermore, we reveal that the superior drought tolerance of *M. sieversii* is associated with two complementary regulatory mechanisms: reduced methylation of the *SCL28.5* promoter and a TATA-box insertion in the *MYB87* promoter that enhances their transactivation. Collectively, our findings elucidate a key transcriptional-epigenetic module governing AR growth and drought resilience, providing a theoretical framework for molecular breeding strategies targeting drought resilience in apple rootstocks.

## Results

### *MsSCL28.5* enhances drought tolerance by promoting the adventitious root growth in ‘M26’

We previously identified *MsSCL28.5* as a candidate gene for drought tolerance via high-throughput sequencing and degradome analysis [43]. To investigate the biological function of *MsSCL28.5* in drought tolerance, we generated transgenic lines overexpressing *MsSCL28.5* in the apple rootstock ‘M26’ via *Agrobacterium tumefaciens*-mediated transformation. Three independent OE lines (OE-20, OE-31, and OE-32) were obtained, which exhibited 2.1 to 3.5-fold higher *MsSCL28.5* transcript levels compared to the non-transgenic ‘M26’ plants (Supplementary Fig. S1A). Four-month-old, well-grown ‘M26’ plants and three *MsSCL28.5*-OE lines were subjected to a 14-day drought stress treatment. During drought stress treatment, soil relative water content (SRWC) was measured (Supplementary Fig. S1B). Under well-watered conditions, the growth of ‘M26’ and *MsSCL28.5*-OE plants was similar. However, following a 14-day drought treatment, the *MsSCL28.5*-OE plants displayed significantly enhanced drought tolerance, characterized by superior overall growth and a higher leaf relative water content (Fig. 1A and B). The *MsSCL28.5*-OE plants also accumulated more proline and less MDA (malondialdehyde), a marker of cellular oxidative stress, after drought stress (Fig. 1C and D). Drought stress triggers oxidative damage via ROS accumulation. In response, plants activate antioxidant defense systems to mitigate this damage. We found that following drought stress, the activities of key antioxidant enzymes (SOD, catalase [CAT] , POD, and ascorbate peroxidase [APX]) were significantly higher in *MsSCL28.5*-OE plants than in ‘M26’ plants (Supplementary Fig. S2A–D). Consequently, *MsSCL28.5*-OE plants accumulated less superoxide (O_2_^−^) and hydrogen peroxide (H_2_O_2_) after drought stress (Supplementary Fig. S2E and F), which was visually confirmed by nitro blue tetrazolium (NBT) staining and 3,3-diaminobenzidine (DAB) staining (Supplementary Fig. S2G and H).

**Figure 1:**
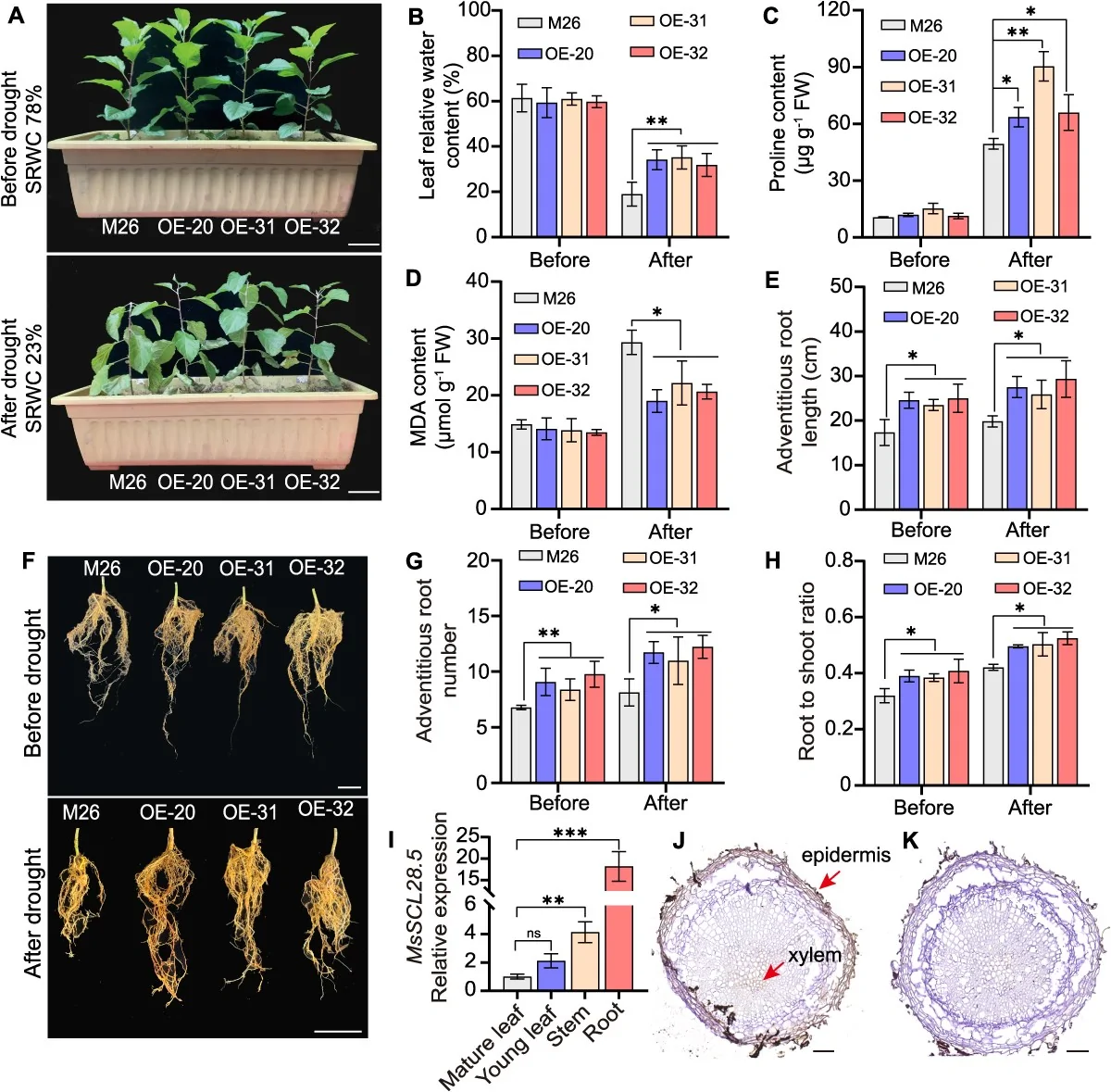
Overexpression of *MsSCL28.5* enhances drought tolerance of ‘M26’ by promoting AR growth. (A) Phenotypes of 4-month-old non-transgenic ‘M26’ and *MsSCL28.5*-OE apple plants before and after a 14-day drought treatment, scale bars = 5 cm. (B) Leaf relative water content, (C) proline content (measured in leaf samples), (D) MDA content (measured in leaf samples), (E) AR length of ‘M26’ and *MsSCL28.5*-OE apple plants. (F) Root phenotypes of 4-month-old ‘M26’ and *MsSCL28.5*-OE apple plants before and after the drought stress, scale bars = 5 cm. (G) Adventitious root number, (H) R/S ratio of ‘M26’ and *MsSCL28.5*-OE apple plants. (I) Tissue-specific expression pattern of *MsSCL28.5*. (J and K) Localization of *MsSCL28.5* in the root tips of ‘M26’ by *in situ* hybridization assay with an antisense probe (J), and sense probe (K), scale bars = 50 μm. Measurements were taken before and after a 14-day drought treatment. Data are represented as means ± SD from three biological replicates. Asterisks indicate significant differences between the *MsSCL28.5*-OE plants and non-transgenic ‘M26’ plants (^*^P < 0.05, ^**^P < 0.01, and ^***^  *P* < 0.001; based on the one-way ANOVA with Tukey’s multiple comparisons test).

Beyond the physiological improvements, *MsSCL28.5* OE also induced significant morphological changes in the root system of transgenic plants. Phenotypic observation revealed that the *MsSCL28.5*-OE plants produced longer and more numerous ARs (Fig. 1E–G) and exhibited a higher R/S ratio (Fig. 1H), compared with ‘M26’ plants, both before and after the drought. Tissue specific expression analysis revealed that *MsSCL28.5* is predominantly expressed in the roots of apple plants (Fig. 1I). To further localize *MsSCL28.5* transcripts within the root, we performed an *in situ* hybridization assay and found that *MsSCL28.5* transcripts localized to the root epidermis and xylem (Fig. 1J and K); this is consistent with its role in AR growth. Longitudinal section analysis of the root tips indicated that the promoted root growth observed in *MsSCL28.5*-OE plants was associated with enhanced cell elongation in the root elongation zone, as the cells in *MsSCL28.5*-OE plants were notably longer than those in ‘M26’ (Supplementary Fig. S3). Furthermore, *MsSCL28.5*-OE plants also demonstrated better performance under mannitol-mediated osmotic stress, accumulating more proline and less MDA, O_2_^−^, and H_2_O_2_ (Supplementary Fig. S4). Collectively, these results demonstrate that the enhanced drought tolerance conferred by *MsSCL28.5* OE is tightly associated with a more vigorous AR system.

### *MsSCL28.5*-transformed rootstock confers drought resistance to wild-type scion

Drought-resistant rootstocks are critical for apple cultivation. To evaluate the agricultural potential of *MsSCL28.5* in rootstock improvement, we assessed its capacity to confer systemic drought tolerance through grafting. We grafted scions of GL-3 (the progeny of *M. domestica* ‘Royal Gala’) onto either ‘M26’ or *MsSCL28.5*-OE rootstocks. For grafting, we selected OE-20 transgenic line, which exhibited the highest relative *MsSCL28.5* levels among the three OE lines generated. Following a 14-day drought treatment, the GL-3/OE-20 grafted plants displayed markedly superior phenotype compared to GL-3/M26 plants, exhibiting less leaf wilting and a significantly higher leaf water content (Fig. 2A and B). Under drought stress, GL-3/OE-20 plants developed longer and more ARs than those in GL-3/M26 plants (Fig. 2C–E), and had a higher R/S ratio (Fig. 2F). The transgenic rootstock also positively influenced scion physiology. Although drought reduced the photosynthetic rate in both graft combinations, the reduction was less severe in GL-3/OE-20 plants (Fig. 2G). GL-3/OE-20 plants maintained a lower stomatal conductance, resulting in improved instantaneous water-use efficiency under drought compared to GL-3/M26 plants (Fig. 2H–I). Moreover, the activities of SOD, CAT, POD, and APX in GL-3/OE-20 plants were significantly higher than those in GL-3/M26 plants (Supplementary Fig. S5A–D). Consequently, the accumulation of O_2_^−^ and H_2_O_2_ were lower in GL-3/OE-20 plants after drought stress, the results confirmed by NBT and DAB staining, respectively (Supplementary Fig. S5E–H). These results demonstrate that the *MsSCL28.5-*OE rootstock systemically enhances the drought resilience of the grafted scion by improved AR development.

**Figure 2:**
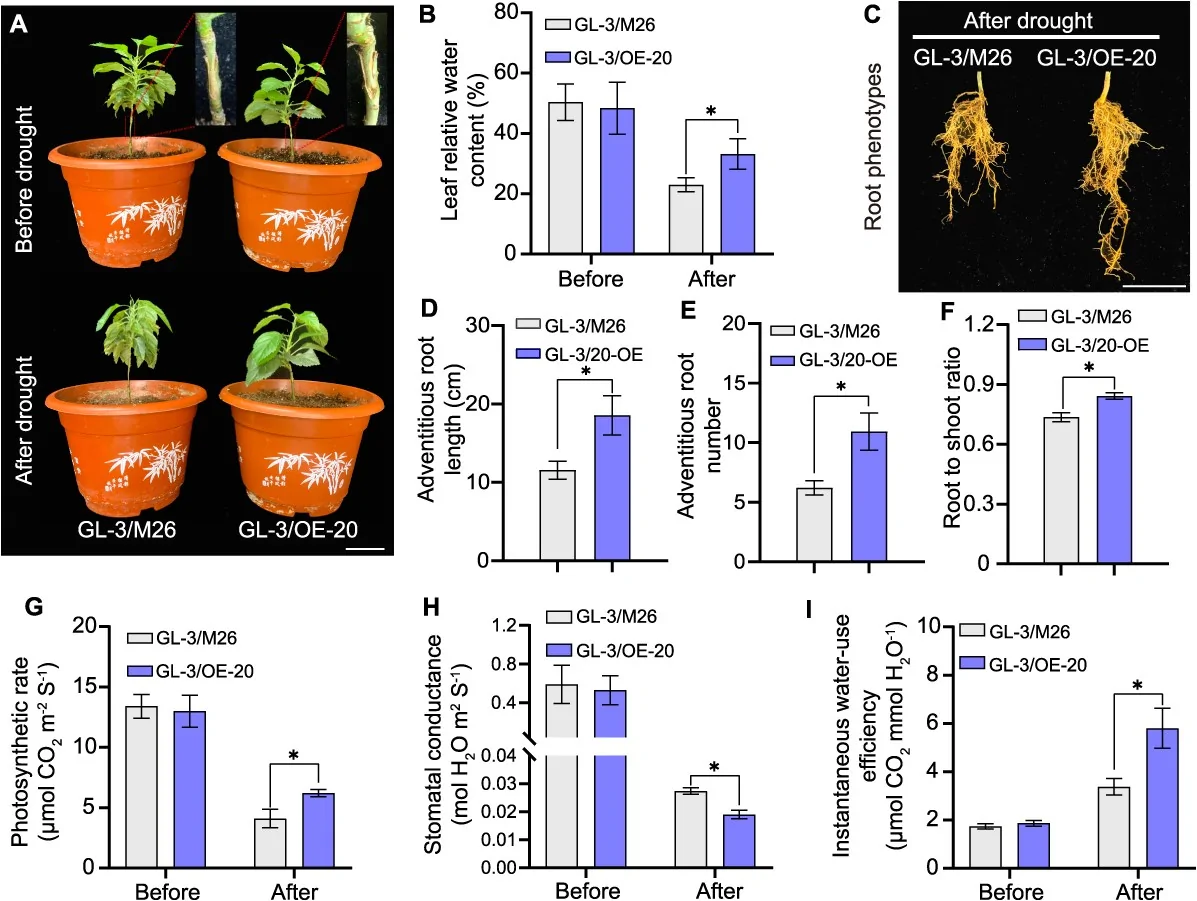
*MsSCL28.5*-OE rootstock enhances drought tolerance of wild-type scion. (A) Phenotypes of 6-month-old GL-3/M26 and GL-3/OE-20 grafted plants before and after a 14-day drought treatment, scale bar = 5 cm. (B) Leaf relative water content of GL-3/M26 and GL-3/OE-20 plants. (C) Root phenotypes of 6-month-old GL-3/M26 and GL-3/OE-20 plants after 14-day drought treatment. (D) Adventitious root length, (E) AR number, (F) R/S ratio of GL-3/M26 and GL-3/OE-20 plants. (G) Net photosynthetic rate (P_n_), (H) stomatal conductance, (H) instantaneous water-use efficiency (WUE_i_) of GL-3/M26 and GL-3/OE-20 plants. Measurements were taken before and after a 14-day drought treatment. Data are represented as means ± SD (*n* = 3 for B, D–F; *n* = 5 for G–I). Asterisks indicate significant differences between the GL-3/OE-20 plants and GL-3/M26 plants (^*^P < 0.05; based on Student’s *t*-test).

### MsSCL28.5 directly binds and activates the *MdLBD16a* and *MdLBD21b* promoters

Given the well-established role of LBD TFs in AR development [44, 45], we hypothesized that MsSCL28.5 might promote root development through this family. We therefore profiled the expression of apple *LBD* genes and found that *MdLBD4c*, *MdLBD7*, *MdLBD16a*, *MdLBD20b*, *MdLBD21a*, *MdLBD21b*, *MdLBD24c*, and *MdLBD41b* were significantly upregulated in the *MsSCL28.5*-OE plants (Supplementary Fig. S6). To elucidate the molecular mechanism underlying MsSCL28.5-mediated root system remodeling, we focused on its potential regulation of key *LBD* genes. A yeast one-hybrid (Y1H) assay revealed that MsSCL28.5 specifically binds to the promoters of *MdLBD16a*, *MdLBD21a*, and *MdLBD21b* (Fig. 3A). *MdLBD16a* and *MdLBD21b* were selected for further validation because the *MdLBD21b* exhibited the most pronounced upregulation in *MsSCL28.5*-OE plants, and *MdLBD21a* shares sequence similarity with *MdLBD21b*. Electrophoretic mobility shift assays (EMSAs) confirmed that MsSCL28.5-GST protein binds to the biotin-labeled probes from the promoters of *MdLBD16a* and *MdLBD21b*. This binding was specifically competed away by an excess of unlabeled probe containing the GAAAAT *cis*-element, establishing it is the precise binding site for MsSCL28.5 (Fig. 3B and C). Additionally, chromatin immunoprecipitation (ChIP)-quantitative polymerase chain reaction (qPCR) assays were conducted using *MsSCL28.5*-OE plants that constitutively express MsSCL28.5-green fluorescent protein (GFP). Upon immunoprecipitation with anti-GFP antibodies, promoter fragments of *MdLBD16a* and *MdLBD21b* encompassing SCL binding sites was significantly enriched in *MsSCL28.5*-OE plants, compared with ‘M26’plants, confirming *in vivo* binding (Fig. 3D–F). Dual-luciferase (LUC) reporter assay was performed to analyze the transcriptional regulation of *MdLBD16a* and *MdLBD21b* by MsSCL28.5. Co-expression of MsSCL28.5 with the *MdLBD16a pro*::LUC or *MdLBD21b pro*::LUC reporters resulted in a significantly stronger luminescence signal and a higher LUC/Renilla luciferase (REN) ratio compared to the empty vector (EV) control (Fig. 3G–K). These results demonstrate that MsSCL28.5 transcriptionally activates the expression of *MdLBD16a* and *MdLBD21b* by directly binding to their promoters.

**Figure 3:**
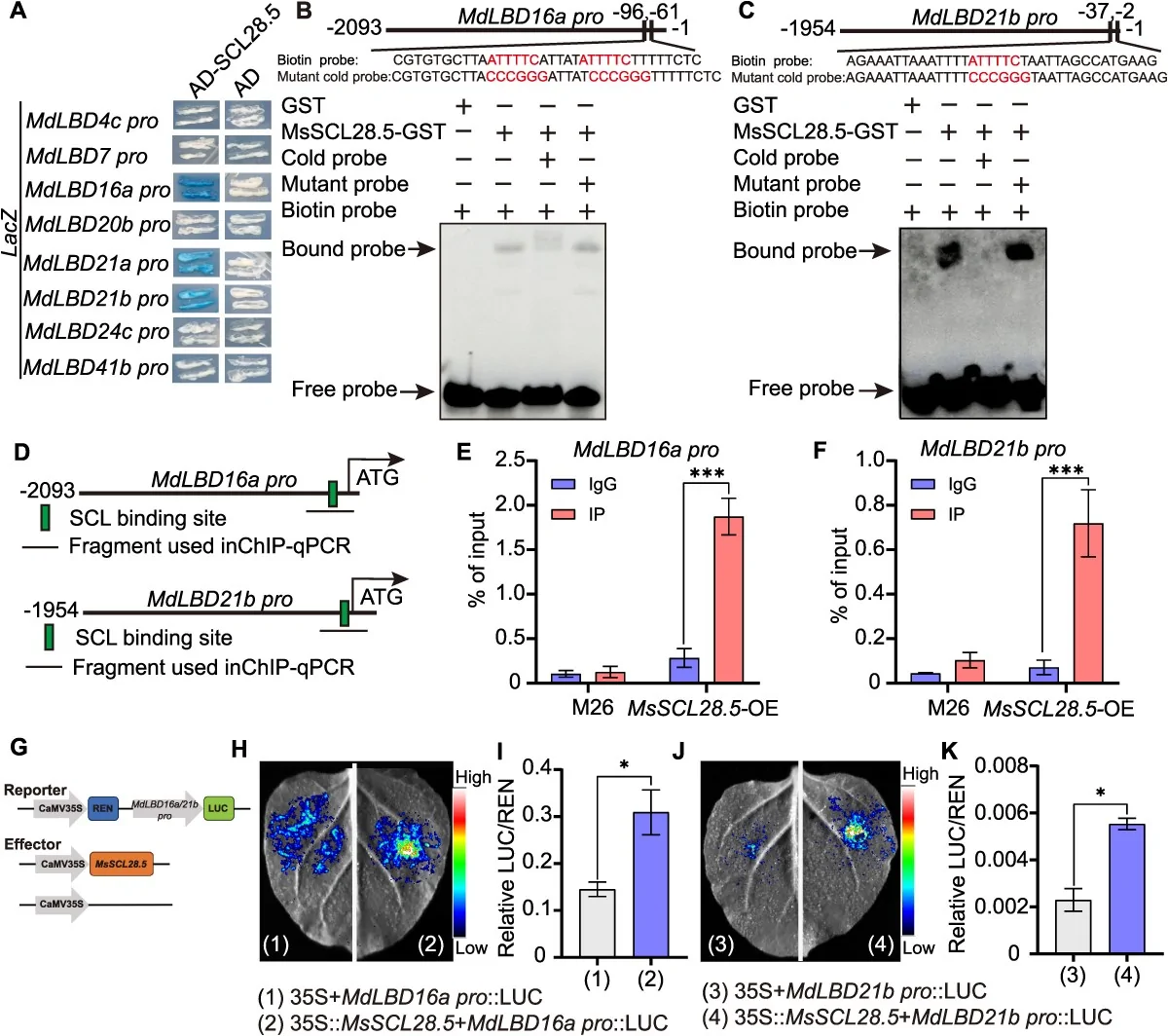
MsSCL28.5 regulates *MdLBD16a* and *MdLBD21b* expression by directly binding to their promoters. (A) Y1H assay showing that MsSCL28.5 directly binds to the *MdLBD16a*, *MdLBD21a*, and *MdLBD21b* promoters. (B and C) EMSA showing that MsSCL28.5-GST protein specifically binds to the biotin-labeled probes from the promoters of *MdLBD16a* (B) and *MdLBD21b* (C). (D–F) ChIP-qPCR validation of *in vivo* binding of MsSCL28.5 to the promoters of *MdLBD16a* and *MdLBD21b*. (D) Schematic diagram of SCL binding sites and qPCR amplicon positions on the promoters of *MdLBD16a* and *MdLBD21b*. (E and F) ChIP-qPCR analysis using leaf tissues from 40-day-old non-transgenic ‘M26’and *MsSCL28.5*-OE tissue-cultured plants. Immunoprecipitation was performed with anti-GFP antibody, and normal rabbit IgG was used as negative control. (E) Enrichment of *MdLBD16a* promoter fragment; (F) enrichment of *MdLBD21b* promoter fragment. Data are represented as means ± SD (*n* = 3). Asterisks indicate significant differences compared with the corresponding IgG control (^***^P < 0.001; based on Student’s *t*-test). (G) Schematic diagram of the effector and reporter constructs used in the dual-LUC assay. (H–K) Dual-LUC assay showing that MsSCL28.5 transactivates *MdLBD16a* and *MdLBD21b* expression in *N. benthamiana* leaves. Representative images of *N. benthamiana* leaves 72 h after infiltration are shown. The relative LUC activities normalized to the REN activity (LUC/REN) were quantified. Data are represented as means ± SD (*n* = 3). Asterisks indicate significant differences compared with the EV control (^*^P < 0.05; based on Student’s *t*-test).

### MsSCL28.5 interacts with MdMYB87 and MsSCL28.5–MdMYB87 complex synergistically activates the *MdLBD16a* and *MdLBD21b* expression

Although MsSCL28.5 localized to the nucleus, it lacks intrinsic transcriptional activation activity in yeast cells (Supplementary Fig. S7). To investigate whether MsSCL28.5 regulates *MdLBD16a/21b* expression through protein–protein interactions, we performed a yeast library screen using MsSCL28.5 as bait to identify its interacting partners. We identified MdMYB87 as a candidate interactor, which was confirmed by a targeted yeast two-hybrid assay (Y2H) (Fig. 4A). The interaction between MdMYB87 and MsSCL28.5 was further validated by luciferase complementation imaging (LCI) assay and *in vitro* by a pull-down assay (Fig. 4B and C). Sequence analysis revealed that MdMYB87 contains conserved R2R3 MYB DNA-binding domains (BDs) (Supplementary Fig. S8). Subcellular localization analysis revealed that MdMYB87 is localized in the nucleus, and has transcriptional activation ability in yeast cells (Supplementary Fig. S9). Notably, the coding sequence of *SCL28.5* is identical between *M. sieversii* and *M. domestica* (Supplementary Fig. S10). Although *MYB87* harbors a single nucleotide polymorphism (SNP) between the two species (Supplementary Fig. S11), this variation does not alter the protein’s ability to interact with SCL28.5 (Supplementary Fig. S12A), indicating that these two proteins can form a functional regulatory module in a natural biological context.

**Figure 4:**
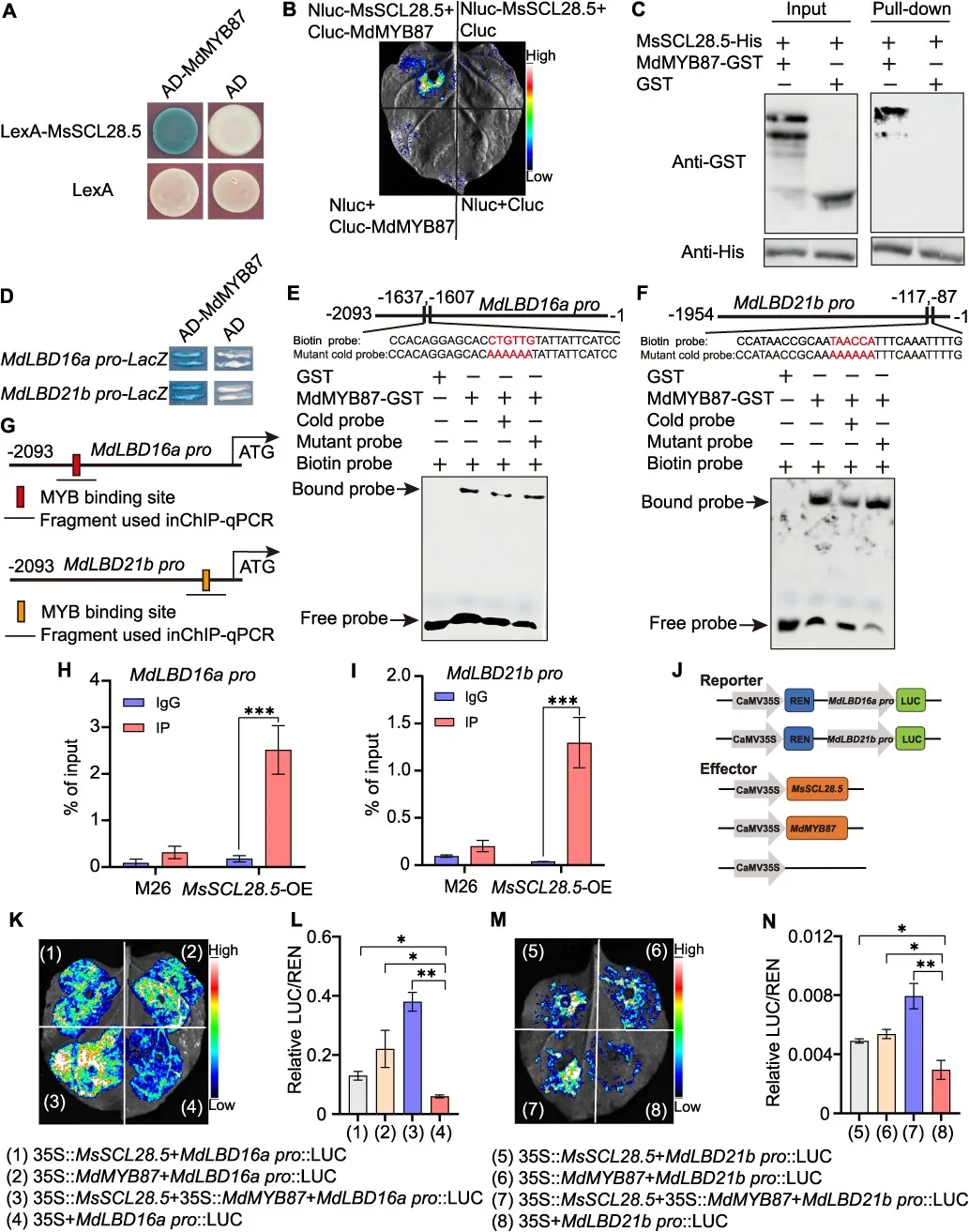
MsSCL28.5 interacts with MdMYB87 and they cooperatively activate *MdLBD16a* and *MdLBD21b* expression. (A) Y2H assay showing that MsSCL28.5 interacts with MdMYB87, MsSCL28.5 was fused with the LexA DNA-BD in pEG202 vector, and MdMYB87 was fused to the AD in pB42AD. LexA and AD served as the negative controls. (B) LCI assay showing the interaction between MsSCL28.5 and MdMYB87 in *N. benthamian*a leaves. MsSCL28.5 was fused to the nLUC fragment, while MdMYB87 was fused to the cLUC fragment. The combinations of MsSCL28.5-nLUC and cLUC, nLUC and MdMYB87-cLUC were used as negative controls. Representative image of *N. benthamiana* leave 72 h after infiltration are shown. Three independent replicates were performed. Data are represented as means ± SD (*n* = 3). (C) Pull-down assay showing that MsSCL28.5 directly interacts with MdMYB87 *in vitro*. The MsSCL28.5-His recombinant protein was incubated with recombinant protein MdMYB87-GST and immobilized with His beads. (D) Y1H assay showing that MdMYB87 directly binds to the *MdLBD16a* and *MdLBD21b* promoters. The LacZ reporter gene was driven by the *MdLBD16a* and *MdLBD21b* promoters. (E and F) EMSA showing that MdMYB87-GST and MdMYB87-His recombinant protein specifically binds to the biotin-labeled probes from *MdLBD16a* and *MdLBD21b* promoters. (G–I) ChIP-qPCR validation of *in vivo* binding of MdMYB87 to the promoters of *MdLBD16a* and *MdLBD21b*. (G) Schematic diagram of MYB binding sites and qPCR amplicon positions on the promoters of *MdLBD16a* and *MdLBD21b*. (H and I) ChIP-qPCR analysis using leaf tissues from 40-day-old non-transgenic ‘M26’and *MdMYB87*-OE tissue-cultured plants. Immunoprecipitation was performed with anti-GFP antibody, and normal rabbit IgG was used as negative control. (H) Enrichment of *MdLBD16a* promoter fragment; (I) enrichment of *MdLBD21b* promoter fragment. Data are represented as means ± SD (*n* = 3). Asterisks indicate significant differences compared with the corresponding IgG control (***P < 0.001; based on Student’s *t*-test). (J) Schematic diagram of the effector and reporter constructs used in the dual-LUC assay. (K–N) Dual-LUC assay showing that co-transformation of MsSCL28.5 and MdMYB87 synergistically transactivates *MdLBD16a* and *MdLBD21b* expression in *N. benthamiana* leaves. Representative images of *N. benthamiana* leaves 72 h after infiltration are shown. The relative LUC activities normalized to the REN activity (LUC/REN) were quantified. Data are represented as means ± SD (*n* = 3). Asterisks indicate significant differences compared with the EV control. (^*^P < 0.05, ^**^P < 0.01; based on the one-way ANOVA with Tukey’s multiple comparisons test).

Interestingly, Y1H and EMSA assay showed that MdMYB87 can also directly bind to the promoters of *MdLBD16a* and *MdLBD21b* (Fig. 4D–F). ChIP-qPCR results further validated this interaction *in vivo*. Upon immunoprecipitation with anti-GFP antibodies, promoter fragments of *MdLBD16a* and *MdLBD21b* encompassing the MYB binding sites were significantly enriched in *MdMYB87*-OE plants, compared with ‘M26’plants (Fig. 4G–I). Since both MsSCL28.5 and MdMYB87 target to the promoters of *MdLBD16a* and *MdLBD21b*, we further investigated their synergistic regulatory effect on *MdLBD16a* and *MdLBD21b* using dual-LUC reporter assay. The results showed that co-expression of MsSCL28.5 and MdMYB87 resulted in a significantly higher LUC/REN ratio and stronger luminescence signal for both the *MdLBD16a pro*::LUC and *MdLBD21b pro*::LUC reporters compared to expression of either protein alone (Fig. 4J–N), indicating synergistic transactivation. Moreover, MdMYB87 and MsMYB87 exhibited equivalent synergistic effects with MsSCL28.5 in transactivating the *LBD16a* promoter (Supplementary Fig. S12B and C), further demonstrating that the SNP variant does not compromise the functional compatibility of the module. Additionally, although MsSCL28.5 and MdMYB87 bind to distinct promoter elements, MdMYB87 enhanced the DNA-binding activity of MsSCL28.5. An EMSA assay showed that the binding signal was increased with the increasing amount of MdMYB87 added to the MsSCL28.5 binding reactions (Supplementary Fig. S13). These data established that MdMYB87 is not only a direct transcriptional activator of *MdLBD16a/21b* but also a cofactor that potentiates MsSCL28.5’s DNA-binding and transactivation capacity.

### *MdMYB87* confers drought tolerance and promotes adventitious root growth in ‘M26’

Given the physical and functional interaction between MsSCL28.5 and MdMYB87, we sought to determine whether MdMYB87 contributes to drought resilience. We generated two independent *MdMYB87*-OE (OE-12 and OE-16) lines in the ‘M26’ rootstock, which exhibited approximately 3-fold higher transcript levels than the non-transgenic ‘M26’ (Supplementary Fig. S14). Following a 14-day drought treatment, the *MdMYB87*-OE plants displayed a pronounced drought-tolerant phenotype, including reduced leaf wilting and a significantly higher leaf relative water content compared to ‘M26’ (Fig. 5A and B). Biochemically, the transgenic lines accumulated more proline and less MDA, indicating enhanced osmo-protection and reduced oxidative damage (Fig. 5C and D). Phenotypic observation revealed that *MdMYB87*-OE plants maintained a higher R/S ratio and developed longer and more numerous ARs compared to ‘M26’ plants both before and after the drought treatment (Fig. 5E–H). This enhanced AR development was associated with the upregulation of *MdLBD16a* and *MdLBD21b*, whose transcript levels were significantly higher in the roots of *MdMYB87*-OE plants (Supplementary Fig. S15). In addition, NBT and DAB staining revealed lower accumulation of O_2_^−^ and H_2_O_2_ in the *MdMYB87*-OE plants under drought stress (Fig. 5I and J; Supplementary Fig. S16A and B). This reduction in ROS levels correlated with elevated activities of key ROS-scavenging enzymes (SOD, CAT, POD, and APX) (Supplementary Fig. S16C–F). Collectively, these results demonstrate that *MdMYB87* functions as a positive regulator of drought tolerance in apple, enhancing resistance through a combination of physiological protection, improved ARs development, and enhanced antioxidant capacity.

**Figure 5:**
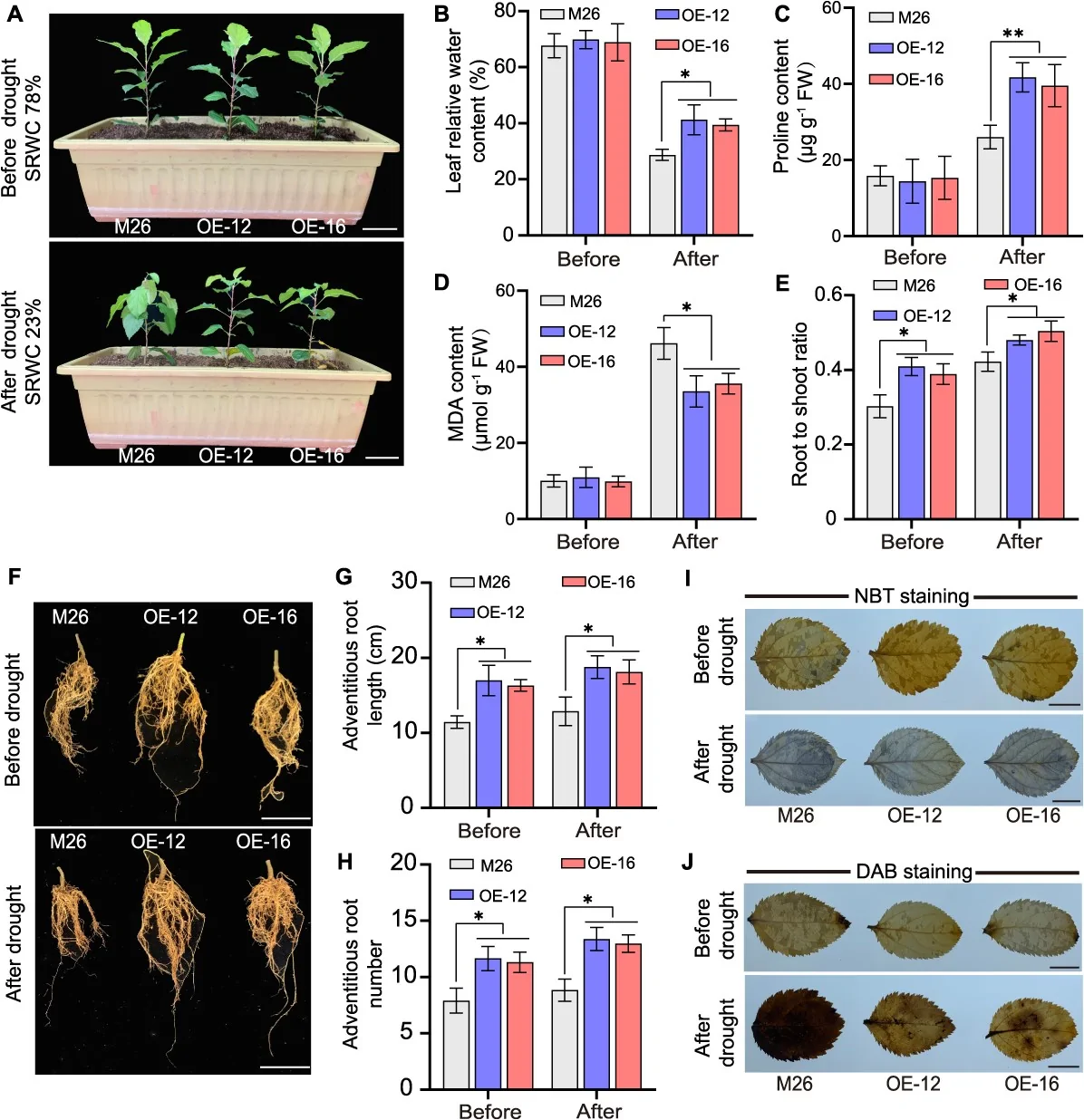
Overexpression of *MdMYB87* enhances drought tolerance of ‘M26’ through increasing AR growth. (A) Phenotypes of 4-month-old non-transgenic ‘M26’ and *MdMYB87*-OE apple plants before and after a 14-day drought treatment, scale bars = 5 cm. (B) Leaf relative water content, (C) proline content, (D) MDA content in ‘M26’ and *MdMYB87*-OE apple plants (measured in leaf samples). (E) R/S ratio of ‘M26’ and *MsSCL28.5*-OE apple plants. (F) Root phenotypes of ‘M26’and *MdMYB87*-OE apple plants 120 days after transplantation into the soil, scale bars = 5 cm. (G) Adventitious root length, (H) AR number of ‘M26’ and *MdMYB87*-OE apple plants. (I) Detection of O_2_^−^ accumulation by NBT staining, scale bars = 1 cm. (J) Detection of H_2_O_2_ accumulation by DAB staining, scale bars = 1 cm. Measurements were taken before and after a 14-day drought treatment. Data are represented as means ± SD (*n* = 3). Asterisks indicate significant differences between the *MdMYB87*-OE plants and non-transgenic ‘M26’ plants. (^*^P < 0.05, ^**^P < 0.01; based on the one-way ANOVA with Tukey’s multiple comparisons test).

### Expression analysis of the SCL28.5-MYB87 module in different apple rootstocks

The superior drought tolerance of the wild apple progenitor *M. sieversii* compared with the cultivated dwarfing rootstock ‘M26’ (*M. domestica*), prompted us to investigate whether differential regulation of the SCL28.5-MYB87 module underlies this phenotypic variation. *SCL28.5* expression was induced by drought in both genotypes but was consistently higher in *M. sieversii* (Fig. 6A). Analysis of the promoter sequences revealed three SNPs in the promoters of *SCL28.5* between the two genotypes; however, these SNPs do not alter known *cis*-regulatory elements (Supplementary Figs S17 and S18), suggesting a potential epigenetic mechanism. Treating plants with the DNA methylation inhibitor 5-aza-2′-deoxycytidine (5-AZA) significantly increased *SCL28.5* expression in *M. domestica* but not in *M. sieversii*, implicating that differential promoter methylation is involved (Fig. 6B). Bisulfite sequencing PCR (BSP) confirmed that a specific region encompassing the fourth CpG island within the *SCL28.5* promoter was highly methylated in *M. domestica* compared to *M. sieversii* (Fig. 6C and D). Notably, drought stress induced a reduction in methylation levels at this locus in both genotypes, although the absolute methylation level remained substantially higher in *M. domestica* than in *M. sieversii* under both control and stress conditions. This finding was further confirmed by McrBC-qPCR analysis (Supplementary Fig. S19). To understand the basis for this differential methylation, we examined the expression of DNA methyltransferases and demethylases. Under drought stress, *M. domestica* exhibited upregulated expression of several DNA methyltransferase genes (*DNMT2*, *CMT2*, *CMT3*, *DMR2*, *MET1a*, and *MET1b*), whereas their expression was reduced or unchanged in *M. sieversii*. Concurrently, the expression of the DNA demethylase *ROS2* was significantly lower in *M. domestica* (Supplementary Fig. S20A). This expression profile is consistent with the hypermethylated state of the *SCL28.5* promoter in *M. domestica*. Furthermore, treating ‘M26’ plants with 5-AZA not only increased *SCL28.5* expression but also significantly enhanced their drought tolerance, reducing leaf wilting (Supplementary Fig. S20B). This demonstrates a direct functional link between DNA methylation and drought resilience. Collectively, these results indicate that the attenuated drought induction of *SCL28.5* in *M. domestica* is associated with promoter hypermethylation, likely driven by an imbalance in the expression of DNA methyltransferases and demethylases.

**Figure 6:**
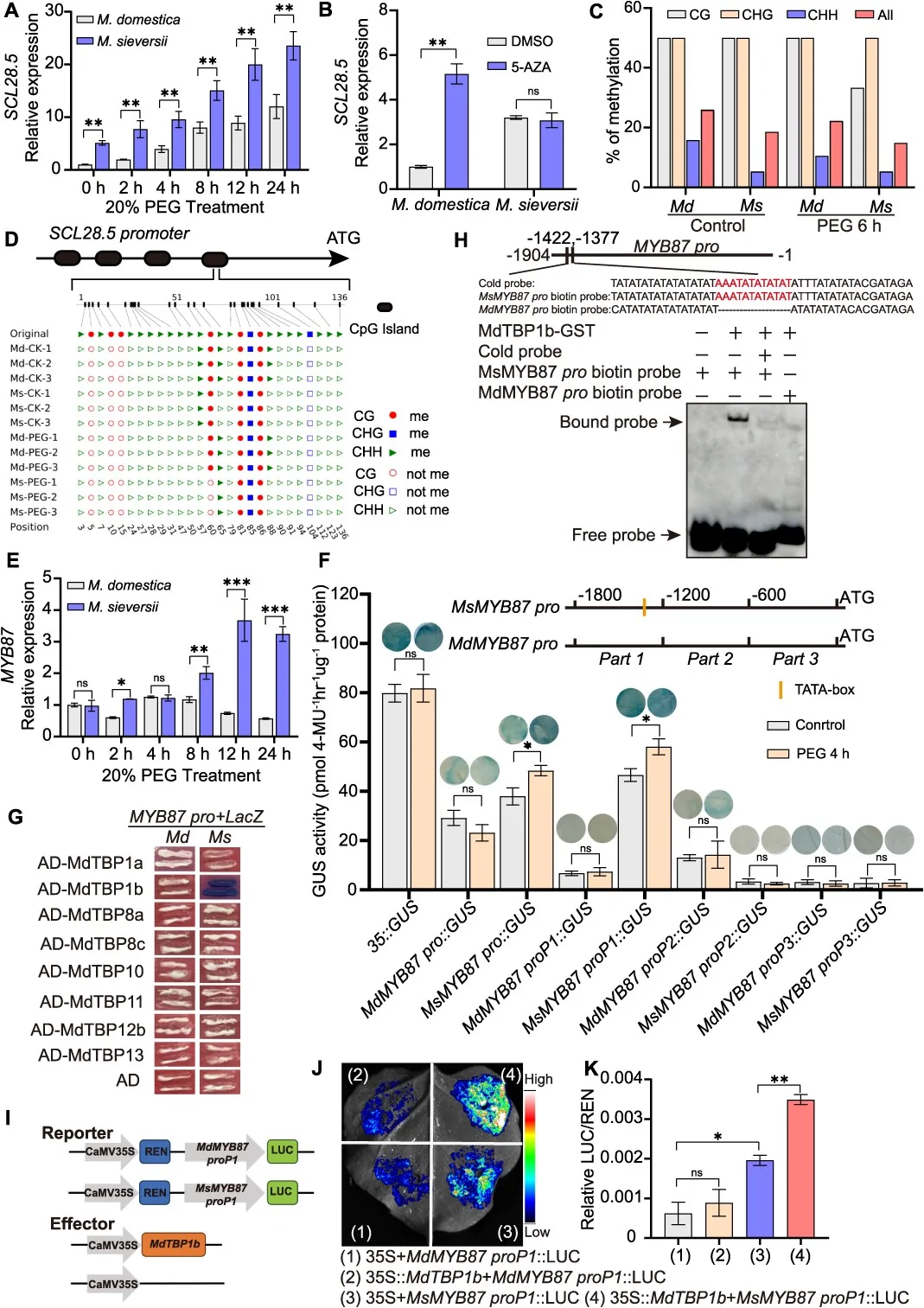
Differential regulation of the SCL28.5-MYB87 module underlies the superior drought tolerance of wild apple (*M. sieversii*). (A) Relative expression levels of *SCL28.5* in *M. sieversi* and the cultivated rootstock ‘M26’ (*M. domestica*) under 20% PEG treatment. (B) Relative expression levels of *SCL28.5* in the two genotypes treated with DMSO or 5-AZA. (C) Methylation level of *SCL28.5* promoter under control or PEG treatment for 6 h analyzed by BSP. (D) Analysis of CpG island structure and DNA methylation sites in the *SCL28.5* promoter. BSP data for the *SCL28.5* promoter from the *M. domestica* and *M. sieversii* under control and PEG-induced drought conditions are shown. Three monoclonal tests were performed, and the methylation sites were analyzed using the online website CyMATE compared with the original sequence. (E) Relative expression levels of *MYB87* in *M. sieversi* and the *M. domestica* under 20% PEG treatment. (F) GUS reporter assays of *MsMYB87* and *MdMYB87* promoter activity in *N. benthamiana* leaves. (G) Y1H assay showing that TBP1b can bind to the *MsMYB87* promoter. (H) EMSA showing TBP1b specifically binds to the Ms*MYB87* promoter. (I) Schematic diagram of the effector and reporter constructs used in the dual-LUC assay. (J and K) Dual-LUC assay showing that TBP1b transactivates *MsMYB87* expression in *N. benthamiana* leaves. Representative images of *N. benthamiana* leaves 72 h after infiltration are shown. The relative LUC activities normalized to the REN activity (LUC/REN) were quantified. Data are represented as means ± SD (*n* = 3). Asterisks indicate significant differences. (^*^P < 0.05, ^**^P < 0.01; A, B, E, and F based on Student’s *t*-test; K based on the one-way ANOVA with Tukey’s multiple comparisons test).

In contrast to the drought-inducible expression of *MsMYB87* in *M. sieversii*, *MYB87* allele in *M. domestica* showed no significant response to drought stress (Fig. 6E). Comparative analysis of the approximately 1.9 kb promoter regions revealed substantial sequence divergence, including multiple SNPs and two deletion sites in the *MdMYB87* promoter (Supplementary Fig. S21). To link these structural variations to functional differences, we first performed a glucuronidase (GUS) reporter assay. This confirmed that the full-length *MsMYB87* promoter had significantly higher basal and polyethylene glycol (PEG)-induced activity compared to the *MdMYB87* promoter (Fig. 6F). To pinpoint the key regulatory region responsible for this activity difference, we divided each promoter into three contiguous fragments of equal length. The GUS activity was predominantly driven by the most distal fragment (Part1) of both promoters, whereas the other two fragments showed minimal activity (Fig. 6F). Within this critical Part1 region, we identified a TATA-box motif present in *M. sieversii* but absent in *M. domestica*. We hypothesized that this structural difference affects the recruitment of general transcription machinery. RNA polymerase II-mediated transcription by the preinitiation complex begins with the binding of the TATA-binding proteins to the TATA-box [46]. Y1H assay identified that TATA-binding protein TBP1b specifically interacts with the *MsMYB87* promoter containing the TATA-box, but not with the corresponding *MdMYB87* promoter lacking this element (Fig. 6G). This specific interaction was confirmed by EMSA *in virto* (Fig. 6H). Furthermore, a dual-LUC assay demonstrated that TBP1b specifically transactivates the *MsMYB87* promoter (Fig. 6I–K). Reverse transcription-quantitative PCR (RT-qPCR) result also showed that *TBP1b* expression is induced by PEG-induced drought stress in both *M*. *sieversii* and *M*. *domestica* (Supplementary Fig. S22). Taken together, these results can explain the higher *MYB87* transcript levels in *M. sieversii* under drought condition. Consistent with the elevated expression of both *SCL28.5* and *MYB87* in *M. sieversii*, the transcript levels of their downstream targets *LBD16a* and *LBD21b* were also significantly higher in *M. sieversii* than in ‘M26’ under drought stress conditions (Supplementary Fig. S23).

The enhanced drought tolerance of *M. sieversii* is associated with a more active SCL28.5-MYB87 module, achieved through two distinct mechanisms, lower methylation of the *SCL28.5* promoter allowing full stress-induced expression, and a promoter structural variant in *MYB87* that enables efficient recruitment of the general transcription machinery via TBP1b, leading to higher and inducible expression.

## Discussion

Drought stress limits plant growth and yield worldwide. To alleviate the detrimental impacts of drought stress, plants developed complex adaptive strategies, including morphological adjustments, such as root architecture remodeling, as well as physiological and molecular responses, such as enhancing antioxidant capacity and regulating stress-related transcriptional networks [18, 33]. Among the key regulators of these processes, GRAS TFs are well-established integrators of plant development and abiotic stress responses [24, 28]. However, their specific roles and mechanisms in apple remain largely unexplored. Here, in this study, the molecular mechanism by which MsSCL28.5, a GRAS TF from *M. sieversii*, regulates drought tolerance in apple was elucidated. We demonstrate that SCL28.5 interacts with the TF MYB87 to form a cohesive transcriptional complex. This SCL28.5-MYB87 module synergistically transactivates the expression of downstream target genes *LBD16a* and *LBD21b* to improve AR growth, thereby conferring enhanced drought tolerance in the cultivated rootstock ‘M26’. This study provides new insights into the interaction mechanisms between GRAS and MYB TFs during stress responses in apple.

Roots are the main organs for plant anchorage and the acquisition of water and nutrients; their architecture is dynamically regulated in response to environmental cues, including drought [47]. In *A. thaliana*, the TF LBD16 is a well-established key regulator of lateral root initiation, functioning as a direct downstream target of the auxin response factors ARF7 and ARF19 within the canonical auxin signaling pathway [31, 48]. In apple, we demonstrate that drought attenuates root auxin levels (Supplementary Fig. S24) while concurrently inducing the SCL28.5–MYB87 transcription complex. This module directly and synergistically transactivates *LBD16a* and *LBD21b* to drive AR development, ensuring a vital growth response under drought stress. This finding aligns with emerging evidence that *LBD* genes can be regulated by diverse TFs beyond the core auxin pathway, allowing for the integration of multiple environmental signals [49]. The function of this regulatory module is underpinned by the specific spatial expression of its components. Tissue-specific analysis revealed that *MsSCL28.5* is predominantly expressed in roots, and *in situ* hybridization further localized strong signals to the epidermal and xylem cells of the root tip (Fig. 1I–K). This expression pattern within root proliferative zones is consistent with reports that *SCL28* is often active in developing organs, and directly influence root morphogenesis [50]. Consequently, *MsSCL28.5-*OE plants exhibited a pronounced cellular phenotype in the root elongation zone, with significantly greater cell lengths compared to ‘M26’ plants (Supplementary Fig. S3A–C). Given the established role of SCL28 proteins in regulating cell cycle progression, the observed promotion of both AR development and cell elongation may stem from altered cell cycle dynamics or a specific function in cell expansion [29]. However, this argument should await further investigation.

Grafting is a common method for improving scion growth, early flowering, fruit quality and stress resistance by combining a desirable scion with a resilient rootstock [41, 51, 52]. In walnut (*Juglans regia*), transgenic rootstocks can systemically modulate the physiology of the grafted scion through long-distance signaling of GA under drought stress [42]. In our grafting experiment, we found that when GL-3 scions grafted onto *MsSCL28.5*-OE rootstocks (GL-3/OE-20) exhibited significantly enhanced drought resilience compared with those on non-transgenic ‘M26’ (GL-3/M26) rootstocks (Fig. 2A and B). This systemic improvement is driven by the engineered rootstock’s superior root architecture and higher R/S ratio (Fig. 2C–F), which enhances water and nutrient acquisition. Consequently, the GL-3/OE-20 grafted plants maintained higher photosynthetic rates and water-use efficiency in the scion under drought stress (Fig. 2G–I). Additionally, GL-3/OE-20 grafted plants exhibited reduced oxidative damage through a more robust antioxidant system in the scion (Supplementary Fig. S5). Our findings confirm that the *MsSCL28.5* OE not only functions within the rootstock but also transmits a potent drought-adaptive signal across the graft union, establishing a solid foundation for its application in breeding stress-tolerant apple rootstocks. In addition, grafting is a powerful tool for dissecting long-distance signaling molecules in plants [42, 53]. Notably, mRNAs of certain GRAS family TFs, particularly *GAI*, have been documented to traffic across graft unions in apple and pear (*Pyrus bretschneideri* Bunge) [54, 55]. In our study, GL-3/OE-20 grafted plants exhibited superior drought tolerance, attributable not only to the improved root architecture of the OE rootstock but also to systemic physiological changes in the scion, such as reduced stomatal conductance and oxidative damage (Fig. 2; Supplementary Fig. S5). Given that *MsSCL28.5* is a member of the GRAS family, an intriguing hypothesis emerges: *MsSCL28.5* mRNA itself, or mRNAs of its downstream targets, could act as mobile signal molecules from the transgenic rootstock to the wild-type scion. Therefore, investigating the mobility of *MsSCL28.5* mRNA is essential to define the mechanism underlying this graft-transmissible drought tolerance, and will be a pivotal focus of our future work.

The wild apple *M. sieversii* is highly stress-resistant, whereas the widely cultivated dwarfing rootstock ‘M26’ (*M. domestica*) has shallow roots and is susceptible to drought. Understanding the molecular basis of this difference is of great significance for breeding stress-tolerant apple rootstocks. The utilization of natural variation is a key strategy for dissecting the genetic architecture of complex traits and accelerating plant breeding [56]. In apple, variations in promoter sequences have been established as important drivers of phenotypic diversity [18, 57]. In this study, we identified a key *cis*-regulatory variant underlying the differential expression of *MYB87*. A TATA-box motif, present in the proximal promoter region (P1) of *MsMYB87*, is absent in the *MdMYB87* allele of ‘M26’ (Supplementary Fig. S21). This structural deficiency impedes the binding of the general TF TBP1b (Fig. 6G and H), leading to diminished promoter activity and consequently lower, non-inducible *MYB87* transcript levels in *M. domestica* under drought (Fig. 6E). Similar variants have been described in the promoters of *MdMYB44* and *MdIRT1*, which are related to fruit quality and stress adaption in apples, respectively [58, 59]. DNA methylation serves as a critical epigenetic modification that regulates gene transcription without altering the DNA sequence [60], and plays an important role in plant response to wide range of abiotic stresses [61]. Parallel to this sequence-based variation, we uncovered a critical epigenetic layer of regulation for *SCL28.5*. Despite nearly identical promoter sequences (Supplementary Fig. S17), *SCL28.5* exhibits divergent expression pattern between the two genotypes (Fig. 6A). This discrepancy is attributed to differential promoter methylation, as treatment with the methylation inhibitor 5-AZA specifically elevated *SCL28.5* expression in *M. domestica*, but not in *M. sieversii* (Fig. 6B)*.* BSP and McrBC-qPCR analysis confirmed that the *SCL28.5* promoter CpG island containing the key ABRE element is significantly higher methylated in *M. domestica* compared to *M. sieversii*, under both control and drought conditions (Fig. 6C and D; Supplementary Fig. S19). This aligns with established roles of stress-linked promoter methylation in modulating plant stress tolerance [37, 62]. The relative levels of DNA methyltransferases and demethylases are critical factors in determining plant DNA methylation levels. We found that under drought stress, *M. domestica* exhibited upregulated expression of several DNA methyltransferase genes (*DNMT2*, *CMT2*, *CMT3*, *DMR2*, *MET1a*, and *MET1b*), whereas their expression was reduced or unchanged in *M. sieversii*. Concurrently, the expression of the DNA demethylase *ROS2* was significantly lower in *M. domestica* (Supplementary Fig. S20A). However, whether a specific methyltransferase or demethylase directly acts on the *SCL28.5* promoter requires further investigation. Collectively, the superior drought resilience of *M. sieversii* can be attributed to the synergistic action of a lower methylated *SCL28.5* promoter and a TATA-box-containing *MYB87* promoter. This dual configuration ensures high-level production of SCL28.5–MYB87 protein complex under stress. This module synergistically activates the AR development related genes *LBD16a* and *LBD21b*, which results in higher transcript levels of both genes in *M. sieversii* than in *M. domestica* under drought stress (Supplementary Fig. S23). The resulting enhanced root growth provides a tangible physiological advantage, contributing directly to the improved drought tolerance observed in *M. sieversii*.

In summary, we propose a working model in which differential regulation of the SCL28.5-MYB87 module underpins the contrasting drought tolerance between *M. domestica* and *M. sieversii* rootstocks (Fig. 7). In drought-sensitive *M. domestica*, a highly methylated promoter attenuates *SCL28.5* stress-induced expression, and the absence of a key TATA-box in the *MYB87* promoter prevents efficient binding of the transcriptional activator TBP1b, limiting MYB87 expression. Conversely, in the drought-tolerant *M. sieversii*, lower methylated *SCL28.5* promoter permits full stress-induced expression, and a TATA-box motif enables strong TBP1b binding and high *MYB87* expression under stress. The consequent abundance of SCL28.5–MYB87 protein complex synergistically activates the expression of *LBD16a* and *LBD21b.* This enhanced transcriptional activation promotes a more extensive AR system in *M. sieversii*, which contributes to its superior drought tolerance. Our findings present molecular insights into how transcriptional regulation of SCL28.5-MYB87 module are integrated into the drought response of different apple rootstocks.

**Figure 7:**
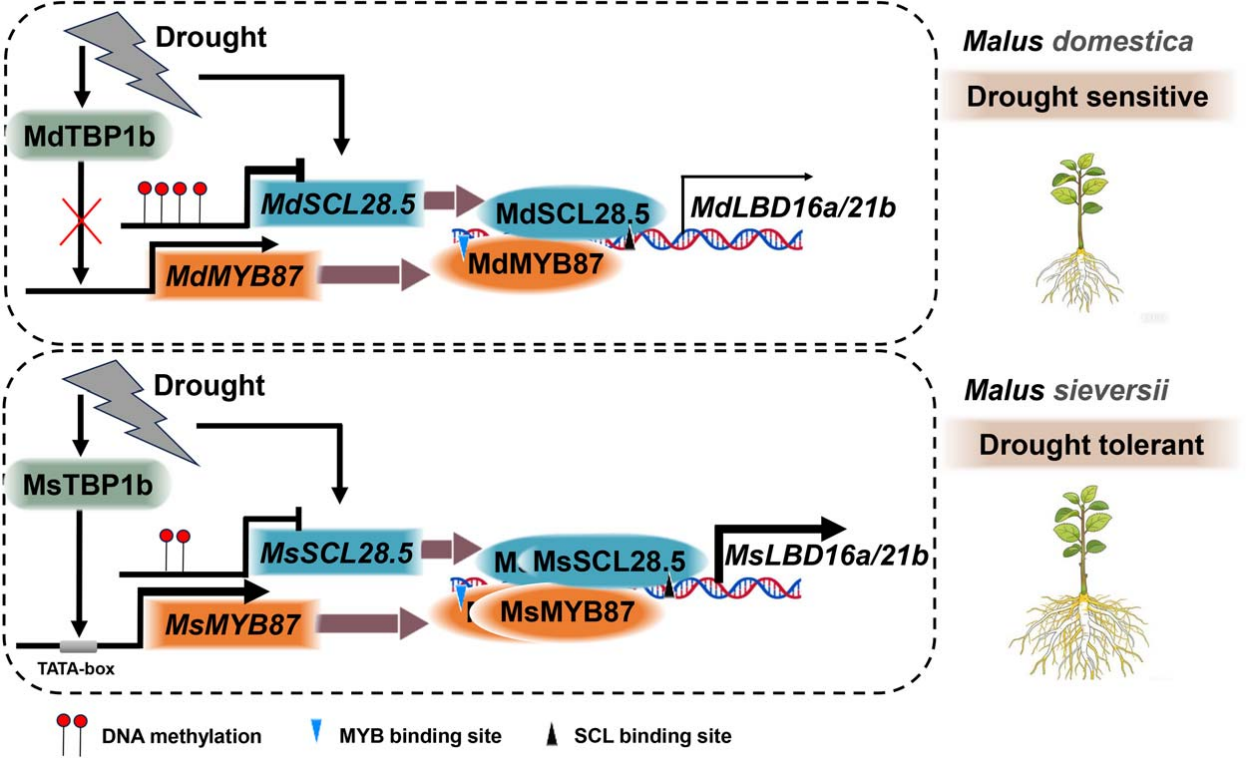
A proposed working model of the role of the SCL28.5-MYB87 module in the drought stress response in different apple rootstocks. In *M. domestica*, highly methylated promoter attenuates *MdSCL28.5* stress-induced expression, and the absence of a key TATA-box in the *MdMYB87* promoter prevents efficient binding of MdTBP1b, thereby limiting MdMYB87 expression. In *M. sieversii*, lower methylated *MsSCL28.5* promoter permits full stress-induced expression, and a TATA-box variant enables strong MsTBP1b binding, driving high *MsMYB87* expression under stress. The resulting higher levels of MsSCL28.5–MsMYB87 protein complex cooperatively binds to and transactivates the promoters of *MsLBD16a* and *MsLBD21a*, which are the key regulators of AR development. This enhanced transcriptional activation promotes a more extensive AR system in *M. sieversii*, contributing to its superior drought tolerance.

## Materials and methods

### Plant material and growth conditions

‘M26’ plants were tissue cultured on Murashige & Skoog (MS) medium [4.43 g/l MS salts, 30 g/l sucrose, 0.3 mg/l 6-benzylaminopurine, 0.2 mg/l 1-naphthaleneacetic acid, 0.5 mg/l gibberellic acid (GA_3_), and 7.5 g/l agar, pH 5.8], under a 14 h/10 h (light/dark) photoperiod, 25°C with 40% air humidity condition. Four-week-old tissue-cultured ‘M26’ plants were used for apple stable genetic transformation as previously reported method with little modifications [43]. Forty-day-old tissue-cultured plants then transplanted into soil for further treatments.

### Construction of overexpression vectors and apple genetic transformation

For the OE vectors, the full-length coding sequences (CDS) of *MsSCL28.5* and *MdMYB87* cloned from the root samples of *M. sieversii* and the ‘M26’ and individually inserted into the PRI101-eGFP vector, resulting in the *MsSCL28.5*-PRI101-eGFP and *MdMYB87*-PRI101-eGFP constructs. The OE plasmids (*MsSCL28.5*-PRI101-eGFP and *MdMYB87*-PRI101-eGFP) were introduced into *A. tumefaciens* strain EHA105 for stable transformation of ‘M26’. Putative transformants were propagated and verified using RT-qPCR.

### Stress treatment

To evaluate drought tolerance, transgenic and non-transgenic apple plants were subjected to both osmotic and natural drought stress conditions. For the osmotic stress treatment, non-transgenic and *MsSCL28.5*-OE plants were grown on MS medium supplemented with or without 300 mM mannitol for 14 days. Plants grown on standard MS medium served as untreated controls. For the natural drought stress treatment, 4-month-old soil-grown plants of the same lines were exposed to drought by withholding water for 14 days. Throughout this period, SRWC was monitored. Leaf samples were collected at the end of the stress period, immediately frozen in liquid nitrogen, and stored for subsequent physiological analysis. In both experiments, each treatment group consisted of nine plants, with three plants pooled as one biological replicate. Three independent biological replicates were performed to ensure statistical robustness.

### Grafting experiment

Grafting was performed in April 2025. Four-month-old soil-grown ‘M26’ plants and *MsSCL28.5*-OE transgenic plants with similar growth status were used as rootstocks. Four-month-old soil-grown GL-3 (the progeny of *M. domestica* ‘Royal Gala’) were used as scions. The rootstocks were topped, leaving a stem segment of ~8 cm in length. The lower lateral leaves were removed, and a longitudinal incision of ~0.5 cm was made at the top cut surface of the rootstock. Scions (~2 cm in length) were excised from nontransgenic GL-3 plants, each bearing a single bud. The basal end of each scion was trimmed into a wedge shape (~1 cm long) and inserted into the incision of the rootstock. The graft union was securely wrapped with plastic film to prevent desiccation. After grafting, the plants were grown under an 18 h/6 h (light/dark) photoperiod at 25°C and 40% relative humidity. Once the graft unions had fully healed, plants with similar growth status were selected for drought stress treatment.

### RNA extraction and RT-qPCR analysis

Total RNA extraction and RT-qPCR was conducted as previously described [5]. *MdActin7* was used as an internal quantification control. Relative expression level was calculated using 2^−ΔΔCt^ method [63]. Three roots collected from three independent plants were pooled as one biological replicate, and three biological replicates with three technical repeats were used. The primers are listed in Supplementary Table S1, and the accession number of genes are listed in Supplementary Table S2 (see online supplementary material).

### *In situ* hybridization assay

Spatial gene expression of *MsSCL28.5* was analyzed by *in situ* hybridization. The experiment was conducted as previously described method [64]. Root tip segments (~0.5 cm from the apex) were collected from 4-week-old, soil-grown ‘M26’ plants. Gene-specific antisense oligonucleotide probe (sequences provided in Supplementary Table S1) was used for hybridization.

### Transcriptional activity analysis and subcellular localization assay

The transcriptional activation of MsSCL28.5 and MdMYB87 were assessed using a yeast system. The CDSs of *MsSCL28.5* and *MdMYB87* were fused to the DNA-BD in the LexA vector. The resulting LexA-MsSCL28.5 and LexA-MdMYB87 construct and an empty pB42AD vector were co-transformed into the yeast reporter strain EGY48. The transformants were screened on selective medium (SD/−Trp−Ura) supplemented with X-gal (catalog no. X0300, Lablead, Beijng, China). To determine the subcellular localization of MsSCL28.5 and MdMYB87, the CDSs (without the stop codon) were cloned into the pRI101 vector to generate the 35S::MsSCL28.5-eGFP and 35S::MdMYB87-eGFP fusion construct. The resulting recombinant plasmids were introduced into *A. tumefaciens* strain GV3101, which was subsequently infiltrated into the *Nicotiana benthamiana* leaves. After a 48-h incubation, the subcellular localization of the fluorescence signals was observed using a confocal laser scanning microscope.

### DNA methylation assay, 5-azacytidine treatment, and bisulfite sequencing PCR

To assess locus-specific DNA methylation, we extracted genomic DNA from *M. sieversii* and ‘M26’ plants using a Plant Genomic DNA Kit (catalog no. A1227A, Tiangen, Beijing, China). Approximately 300 ng of DNA was subsequently treated with sodium bisulfite using an EZ DNA Methylation-Gold Kit (catalog no. D5005, Zymo Research, California, USA). The bisulfite-treated DNA was then amplified by PCR using Ex Taq Mastermix (catalog no. 01032, CoWin, Jiangsu, China). The resulting PCR products were purified from an agarose gel with a Gel Extraction Kit (catalog no. EG101-02, TransGen, Beijing, China), cloned into the pClone007 vector (catalog no. 007VS, TSINGKE, Beijing, China) and sequenced. The cloned sequences were aligned to a reference sequence, and methylation patterns and levels were visualized and calculated using CyMATE online software [65]. The primers are listed in Supplementary Table S1 (see online supplementary material).

To investigate the functional role of DNA methylation, we treated plants with the DNA methyltransferase inhibitor 2′-deoxy-5-AZA (Sigma, A3656, USA). *M. sieversii* and ‘M26’ plants were first acclimated in a half-strength Hoagland nutrient solution for 2 weeks. Subsequently, plants were grown in the same nutrient solution with or without a supplement of 10 μg/ml 5-AZA for 10 days under long-day conditions (14 h light/10 h dark) at 25°C. Following this pretreatment, plants were subjected to a drought stress by applying 20% (w/v) PEG6000 for 0 or 6 h [66, 67].

### McrBC analysis

Genomic DNA was extracted as described above. For each reaction, 700 ng of genomic DNA was digested with 1 μl McrBC (catalog no. 1234A, Takara, Japan) in a 20 μl reaction mixture containing 1× McrBC Buffer, 0.01% BSA, and 1× GTP at 37°C for 4 h. A parallel reaction without McrBC served as a negative control. After heat inactivation at 75°C for 10 min, the digestion products were used as templates for qPCR with primers listed in Supplementary Table S1. The relative levels of DNA retained after McrBC digestion were calculated using the 2^–ΔΔCt^ method with *MdActin7* serving as an internal control, and ΔΔCt was determined as ΔCt(McrBC^+^) – ΔCt(McrBC^−^). Lower values indicate higher methylation levels.

### Yeast library screening and yeast two-hybrid assay

Yeast library screening was performed as previously described [5]. A targeted yeast two hybrid was used to confirm the interaction between MsSCL28.5 and MdMYB87. The CDS of *MsSCL28.5* was cloned into the DNA-BD vector (LexA), while the CDS of *MdMYB87* was cloned into the activation domain (AD) vector (pB42AD). The resulting fusion constructs, LexA-MsSCL28.5 (BD) and pB42AD-MdMYB87 (AD) and help plasmid PSH18, were co-transformed into the yeast reporter strain EGY48, which contains the *p8op::LacZ* reporter gene [68]. Protein interaction was assessed by growing the transformed yeast on selective medium (SD/−Trp−His−Ura) supplemented with X-Gal. The development of a blue color indicated the successful reconstitution of the TF and activation of the *LacZ* reporter, confirming a positive interaction.

### Luciferase complementation imaging assay

Protein–protein interaction between MsSCL28.5 and MdMYB87 was further verified in planta using a firefly LCI assay. The CDS of *MsSCL28.5* was fused to the N-terminal fragment of luciferase (nLUC), while *MdMYB87* was fused to the C-terminal fragment (cLUC). The two constructs were co-expressed in leaves of *N. benthamiana* via *A. tumefaciens*-mediated infiltration (strain GV3101). After infiltration, plants were maintained under continuous darkness for 12 h and then replaced it under light for 36 h to assess the interaction [68]. Luciferase activity was then visualized and captured using a Night SHADE LB985 Plant Imaging System (Berthold) [5].

### Pull-down assay

Pull-down assay was conducted to confirm the direct interaction between MsSCL28.5 and MdMYB87. The CDSs of *MsSCL28.5* and *MdMYB87* were cloned into pET32a and pGEX-4 T-1vectors, respectively, to generate MsSCL28.5-His and MdMYB87-GST fusion constructs. These constructs were expressed in the *Escherechia coli* Rosetta E3 strain [5, 69]. For the binding assay, MsSCL28.5-His protein was immobilized onto His-tag purification beads (catalog no. 70601, Beaver Bio, Suzhou, China), and incubated with MdMYB87-GST protein at 4°C for 1 h. Following incubation, the beads were washed three times with washing buffer to remove non-specifically bound proteins. Bound proteins were analyzed by western blot using an anti-GST/His antibody. A sample with purified GST protein alone was included as a negative control.

### Yeast one-hybrid assay

The CDS of *MsSCL28.5*, *MdMYB87*, and *MdTBPs* were individually inserted into pB42AD vector. Promoters of *MdLBDs*, *MdMYB87*, and *MsMYB87* were separately cloned into the pLacZi2μ vector, and co-transformed into yeast strain EGY48. Y1H assay was performed as described [69].

### Dual-luciferase assay

The CDSs of *MsSCL28.5*, *MdMYB8*,*7* and *MdTBP1b* were individually cloned into the pGreenII 0029 62-SK vector to generate effector constructs. The promoters of *MdLBD16a*, *MdLBD21b*, and P1 fragments of *MdMYB87*, *MsMYB87* were fused upstream of the firefly *LUC* gene in the pGreenII 0800-LUC vector to create the reporter constructs. These effector and reporter constructs were co-expressed in leaves of *N. benthamiana* via *A. tumefaciens*-mediated transient transformation, with an empty effector vector was included as a negative control [5, 18, 69]. After incubation, LUC activity was visualized using a chemiluminescence analysis system (Night SHADE, LB985, Berthold, Germany). and LUC/REN ratio were determined using Dual-luciferase Reporter Assay Kit (catalog no. DL101-01, Vazyme, Nanjing, China) following the instructions of manufacturer.

### Electrophoretic mobility-shift assay

EMSA were conducted to verify the direct binding of MsSCL28.5 and MdMYB87 to the promoters of *MdLBD16a* and *MdLBD21b* and binding of MdTBP1b to the promoter of *MdMYB87*and *MsMYB87*. The assay was performed using a Chemiluminescent EMSA Kit (catalog no. GS009, Beyotime, Shanghai, China) following the manufacturer’s instructions. Briefly, biotin-labeled DNA probes, corresponding to the promoter regions of *MdLBD16a*, *MdLBD21b*, *MdMYB87*, and *MsMYB87* that contain the putative recognition sites for GRAS, MYB, and TBP TFs, were synthesized by Sangon Biotech (Beijing, China). These labeled probes were incubated with the recombinant proteins at room temperature for 20 minutes. To test binding specificity, competition assays were included by adding a substantial excess of unlabeled probes (cold competitors) to the binding reaction. The DNA–protein complexes were resolved from on 8% non-denaturing polyacrylamide gels and visualized using the chemiluminescence detection system.

### Chromatin immunoprecipitation-quantitative polymerase chain reaction assay

ChIP-qPCR was performed using a Smart-ChIP™ Kit (catalog no. ChIP1001, Engibody, Shanghai, China) according to the manufacturer’s instructions. In brief, leaves from 40-day-old tissue-cultured transgenic plants expressing *MsSCL28.5-GFP*, *MdMYB87-GFP*, and non-transgenic ‘M26’ plants were cross-linked in 1% formaldehyde. The isolated chromatin was sheared by sonication. Immunoprecipitation was conducted with an anti-GFP antibody and normal rabbit immunoglobulin G (IgG) was used as a negative control. After reverse cross-linking and protein removal, the recovered DNA was analyzed by qPCR. Enrichment was quantified using the %input method. Ct values were normalized to input chromatin; ΔCt = Ct (input) – Ct (IP), and %input was calculated as 2^(ΔCt)^ × 100%. Primers are listed in Supplementary Table S1.

### β-Glucuronidase reporter assay

The promoters of *MsMYB87* and *MdMYB87* were first cloned from the roots of *M. sieversii* and ‘M26’, divided into same-length three fragments, and the full-length promoters and different fragments were inserted into *EcoR* I/*Nco* I site of the pCAMBIA1301 vector, which contains the GUS reporter gene. Vectors were transformed into *A. tumefaciens* strain GV3101. Transient transformation in *N. benthamiana* leaves was performed as described [18]. Transgenic *N. benthamiana* plants were treated with 20% PEG6000 (W/V) solution for 6 h. The histochemical staining of GUS and GUS activity analysis were performed as described [33].

### Measurement of the adventitious root length, number, and root to shoot ratio

The plants were carefully removed from the pots, and the root systems were gently washed with water to remove adhering soil. The length of AR was measured from the base of the stem to the root tip using a ruler, and the longest AR per plant was recorded. For AR number, only ARs emerging directly from the basal stem were counted, and lateral roots branching from these primary ARs were excluded. Three independent biological replicates (each consisting of three plants per line) were evaluated, and the average number of ARs per plant was calculated. For R/S ratio determination, plants were destructively harvested after drought treatment or under normal growth conditions. Each plant was separated into aboveground (stem and leaves) and belowground (roots) parts. The samples were dried to constant weight at 65°C. Each sample was weighed using an electronic balance. The R/S ratio was calculated using the following equation: R/S ratio = belowground dry weight/aboveground dry weight.

### Measurement of the physiological index and auxin content

Measurement of the SOD, CAT, POD, and APX activities were performed as previously described [3]. Proline and MDA contents were measured as previously described [5]. O_2_^−^ and H_2_O_2_ contents were quantified as previously described [70]. NBT and DAB staining were employed to visualize the accumulated O_2_^−^ and H_2_O_2_ according to previous methods [18]. Auxin (indole-3-acetic acid, IAA) content was detected by using the ELISA kit (catalog no. JLC11318, Gelatins, Shanghai, China).

### Determination of photosynthetic parameters and leaf relative water content

Before or after the drought stress, the photosynthetic parameters of grafted plants were monitored by using a photosynthetic apparatus (Li-6400, LICOR, Huntington Beach, CA, USA); five measurements were performed for each group as previously described. The leaf relative water content was determined as previously described [71].

### Statistical analysis

All statistical analysis were performed by using the GraphPad Prism V7 (https://www.graphpad.com/scientific-software/prism/), For multiple comparisons, one-way analysis of variance (ANOVA) with Tukey’s multiple comparisons test was used. For pairwise comparisons between two groups, two-tailed Student’s *t*-test was used. Three independent biological replicates were performed for each determination. Data are shown as mean ± standard deviation (SD), differences asterisks between these results were considered as statistically significant (^*^P < 0.05, ^**^P < 0.01, and ^***^  *P* < 0.001).

## Supplementary Material

Web_Material_uhag211

## Data Availability

All relevant data supporting our findings are available in the manuscript and supplementary materials.
