# Supplementary material for: The MsSCL28.5–MdMYB87 module enhances drought tolerance by promoting adventitious root development in apple rootstocks
Source: Hortic Res. 2026 Jun 2;13(11):uhag211. doi: 10.1093/hr/uhag211 (PMC13718307; doi:10.1093/hr/uhag211)
Supplement: Supplementary file 1 [file web_material_uhag211.zip › Supplemental Figure S1.pdf]

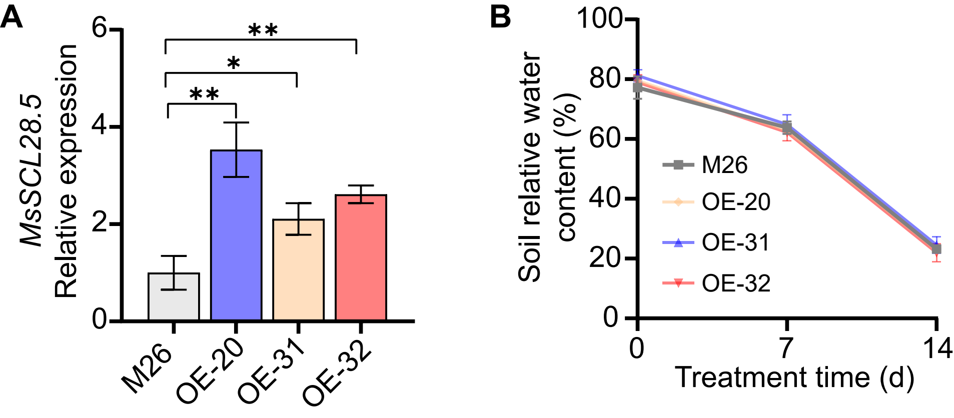
A
MsSCL28.5
Relative expression
6
4
2
0
**
*
**
M26
OE-20
OE-31
OE-32
B
Soil relative water content (%)
100
80
60
40
20
0
M26
OE-20
OE-31
OE-32
0
7
14
Treatment time (d)
