# Supplementary material for: The MsSCL28.5–MdMYB87 module enhances drought tolerance by promoting adventitious root development in apple rootstocks
Source: Hortic Res. 2026 Jun 2;13(11):uhag211. doi: 10.1093/hr/uhag211 (PMC13718307; doi:10.1093/hr/uhag211)
Supplement: Supplementary file 1 [file web_material_uhag211.zip › Supplemental Figure S2.pdf]

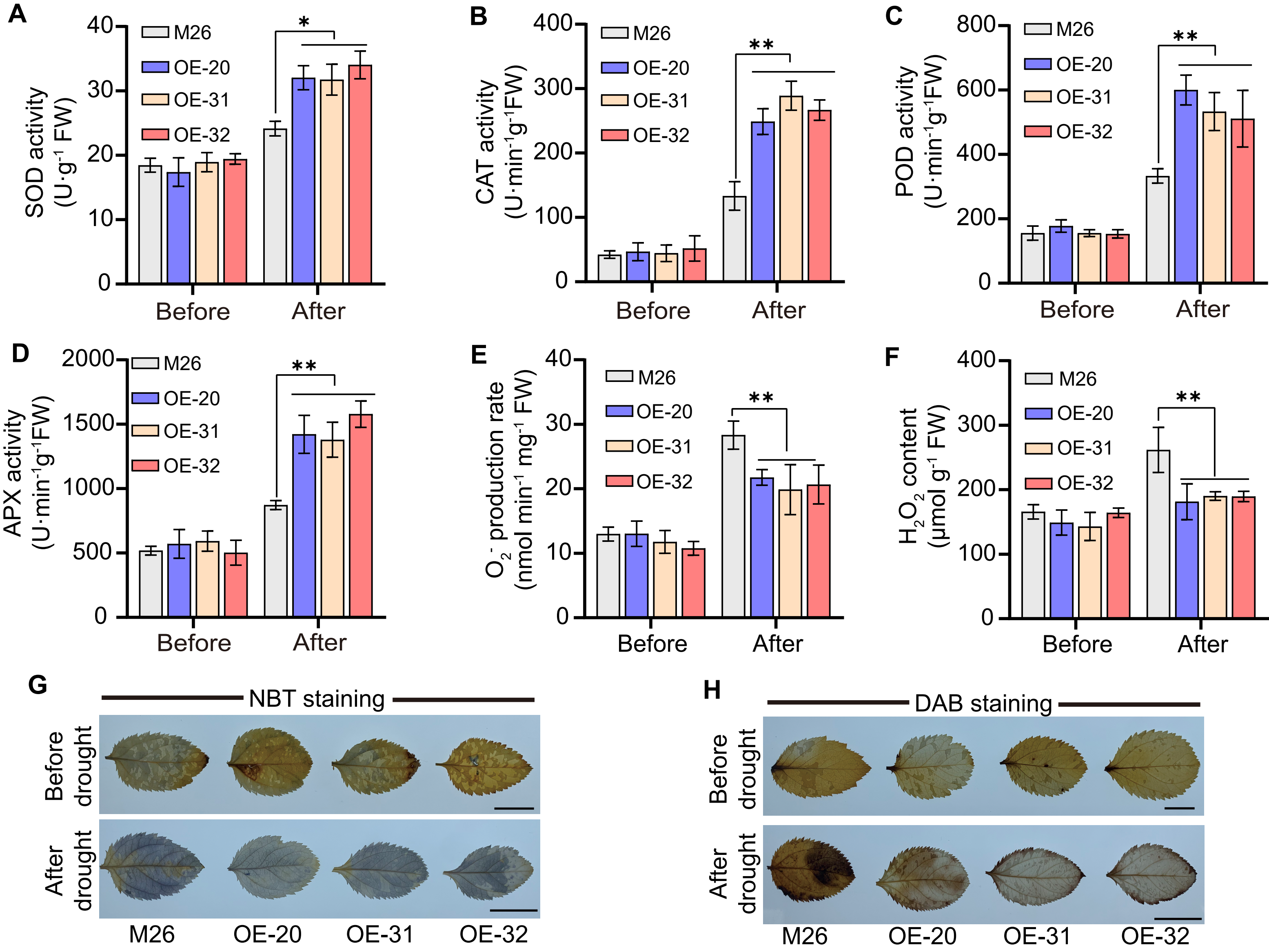

A
SOD activity (U·g-1 FW)
M26
OE-20
OE-31
OE-32
*
Before
After
B
CAT activity (U·min-1g-1FW)
**
C
POD activity (U·min-1g-1FW)
**
D
APX activity (U·min-1g-1FW)
**
E
O2- production rate (nmol min-1 mg-1 FW)
**
F
H2O2 content (μmol g-1 FW)
**
G
NBT staining
Before drought
After drought
H
DAB staining
M26
OE-20
OE-31
OE-32
