# Supplementary material for: The MsSCL28.5–MdMYB87 module enhances drought tolerance by promoting adventitious root development in apple rootstocks
Source: Hortic Res. 2026 Jun 2;13(11):uhag211. doi: 10.1093/hr/uhag211 (PMC13718307; doi:10.1093/hr/uhag211)
Supplement: Supplementary file 1 [file web_material_uhag211.zip › Supplemental Figure S3.pdf]

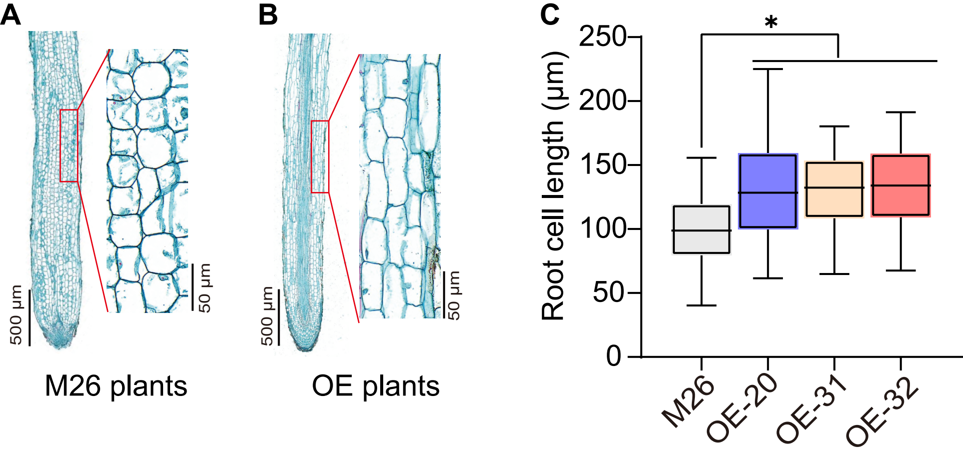

A
B
C
500 µm
50 µm
500 µm
50 µm
M26 plants
OE plants
Root cell length (µm)
250
200
150
100
50
0
*
M26
OE-20
OE-31
OE-32
