# Supplementary material for: The MsSCL28.5–MdMYB87 module enhances drought tolerance by promoting adventitious root development in apple rootstocks
Source: Hortic Res. 2026 Jun 2;13(11):uhag211. doi: 10.1093/hr/uhag211 (PMC13718307; doi:10.1093/hr/uhag211)
Supplement: Supplementary file 1 [file web_material_uhag211.zip › Supplemental Figure S5.pdf]

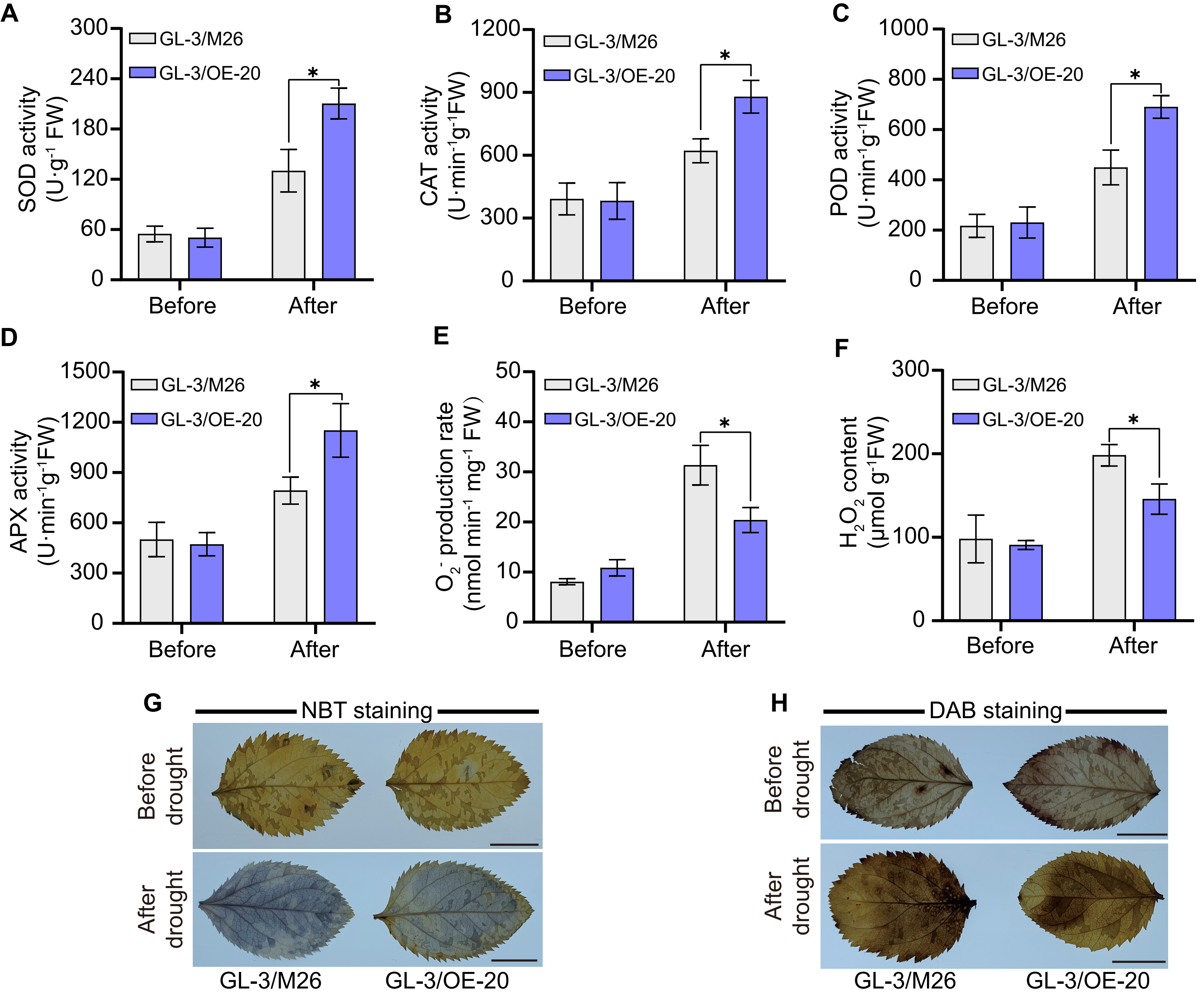
A
SOD activity
(U·g-1 FW)
GL-3/M26
GL-3/OE-20
Before
After
B
CAT activity
(U·min-1g-1FW)
C
POD activity
(U·min-1g-1FW)
D
APX activity
(U·min-1g-1FW)
E
O2- production rate
(nmol min-1 mg-1 FW)
F
H2O2 content
(μmol g-1FW)
G
NBT staining
Before drought
After drought
H
DAB staining
GL-3/M26
GL-3/OE-20
