# Supplementary material for: The MsSCL28.5–MdMYB87 module enhances drought tolerance by promoting adventitious root development in apple rootstocks
Source: Hortic Res. 2026 Jun 2;13(11):uhag211. doi: 10.1093/hr/uhag211 (PMC13718307; doi:10.1093/hr/uhag211)
Supplement: Supplementary file 1 [file web_material_uhag211.zip › Supplemental Figure S6.pdf]

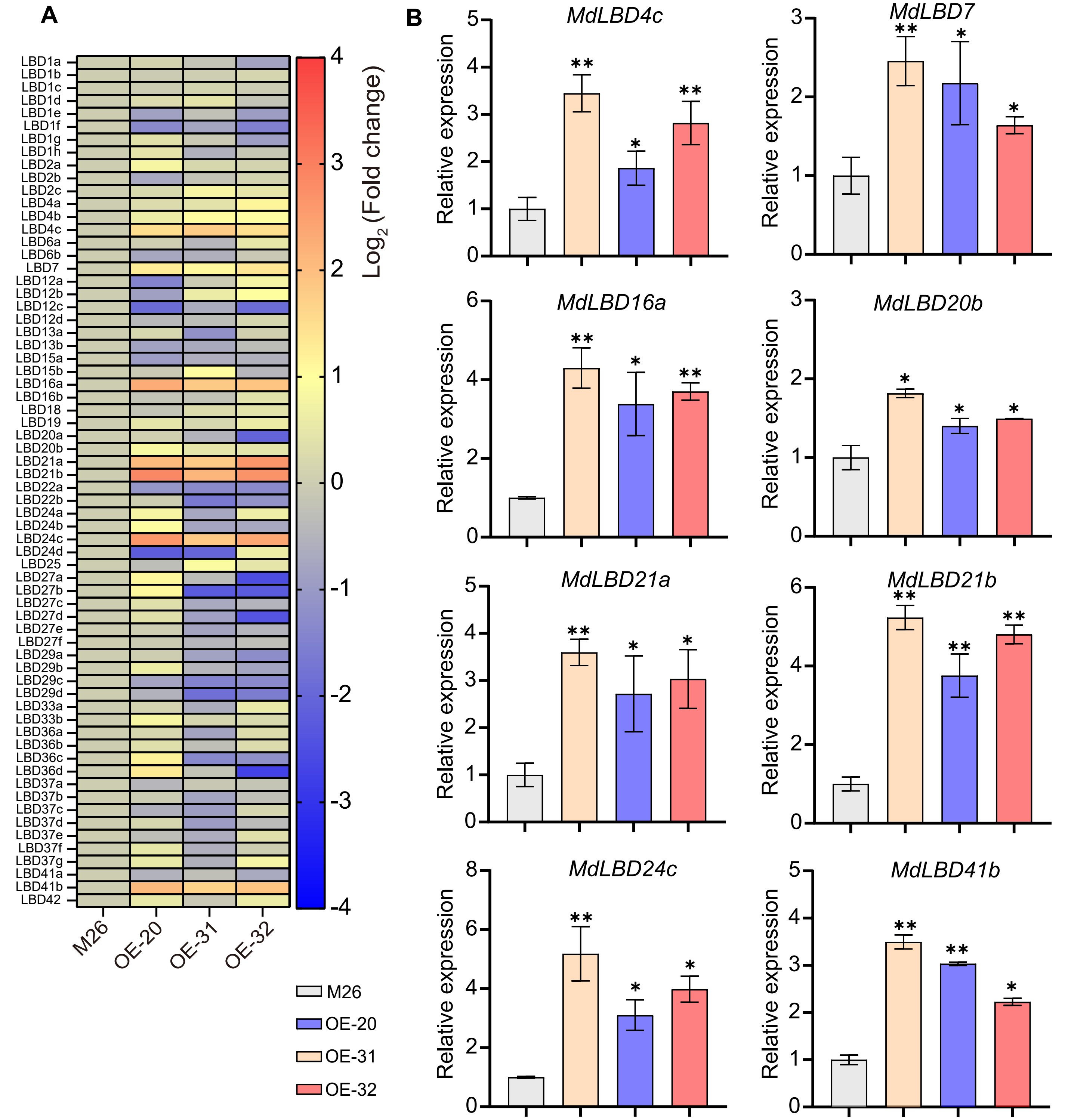
A
LBD1a
LBD1b
LBD1c
LBD1d
LBD1e
LBD1f
LBD1g
LBD1h
LBD2a
LBD2b
LBD2c
LBD4a
LBD4b
LBD4c
LBD6a
LBD6b
LBD7
LBD12a
LBD12b
LBD12c
LBD12d
LBD13a
LBD13b
LBD15a
LBD15b
LBD16a
LBD16b
LBD18
LBD19
LBD20a
LBD20b
LBD21a
LBD21b
LBD22a
LBD22b
LBD24a
LBD24b
LBD24c
LBD24d
LBD25
LBD27a
LBD27b
LBD27c
LBD27d
LBD27e
LBD27f
LBD29a
LBD29b
LBD29c
LBD29d
LBD33a
LBD33b
LBD36a
LBD36b
LBD36c
LBD36d
LBD37a
LBD37b
LBD37c
LBD37d
LBD37e
LBD37f
LBD37g
LBD41a
LBD41b
LBD42
M26
OE-20
OE-31
OE-32
Log_2 (Fold change)
4
3
2
1
0
-1
-2
-3
-4
B
MdLBD4c
MdLBD7
MdLBD16a
MdLBD20b
MdLBD21a
MdLBD21b
MdLBD24c
MdLBD41b
Relative expression
M26
OE-20
OE-31
OE-32
