# Supplementary material for: The MsSCL28.5–MdMYB87 module enhances drought tolerance by promoting adventitious root development in apple rootstocks
Source: Hortic Res. 2026 Jun 2;13(11):uhag211. doi: 10.1093/hr/uhag211 (PMC13718307; doi:10.1093/hr/uhag211)
Supplement: Supplementary file 1 [file web_material_uhag211.zip › Supplemental Figure S7.pdf]

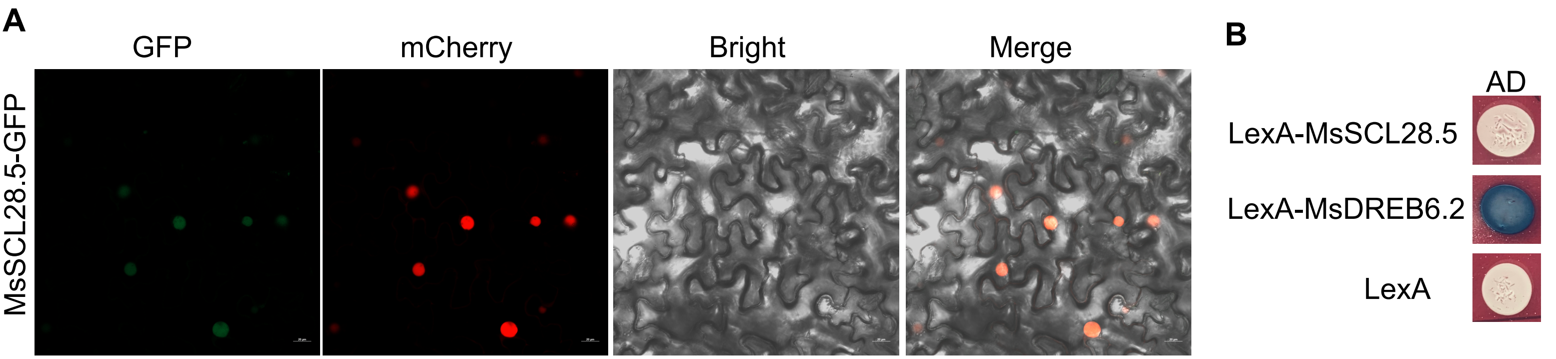
A
GFP
mCherry
Bright
Merge
MsSCL28.5-GFP
B
AD
LexA-MsSCL28.5
LexA-MsDREB6.2
LexA
