# Supplementary material for: The MsSCL28.5–MdMYB87 module enhances drought tolerance by promoting adventitious root development in apple rootstocks
Source: Hortic Res. 2026 Jun 2;13(11):uhag211. doi: 10.1093/hr/uhag211 (PMC13718307; doi:10.1093/hr/uhag211)
Supplement: Supplementary file 1 [file web_material_uhag211.zip › Supplemental Figure S8.pdf]

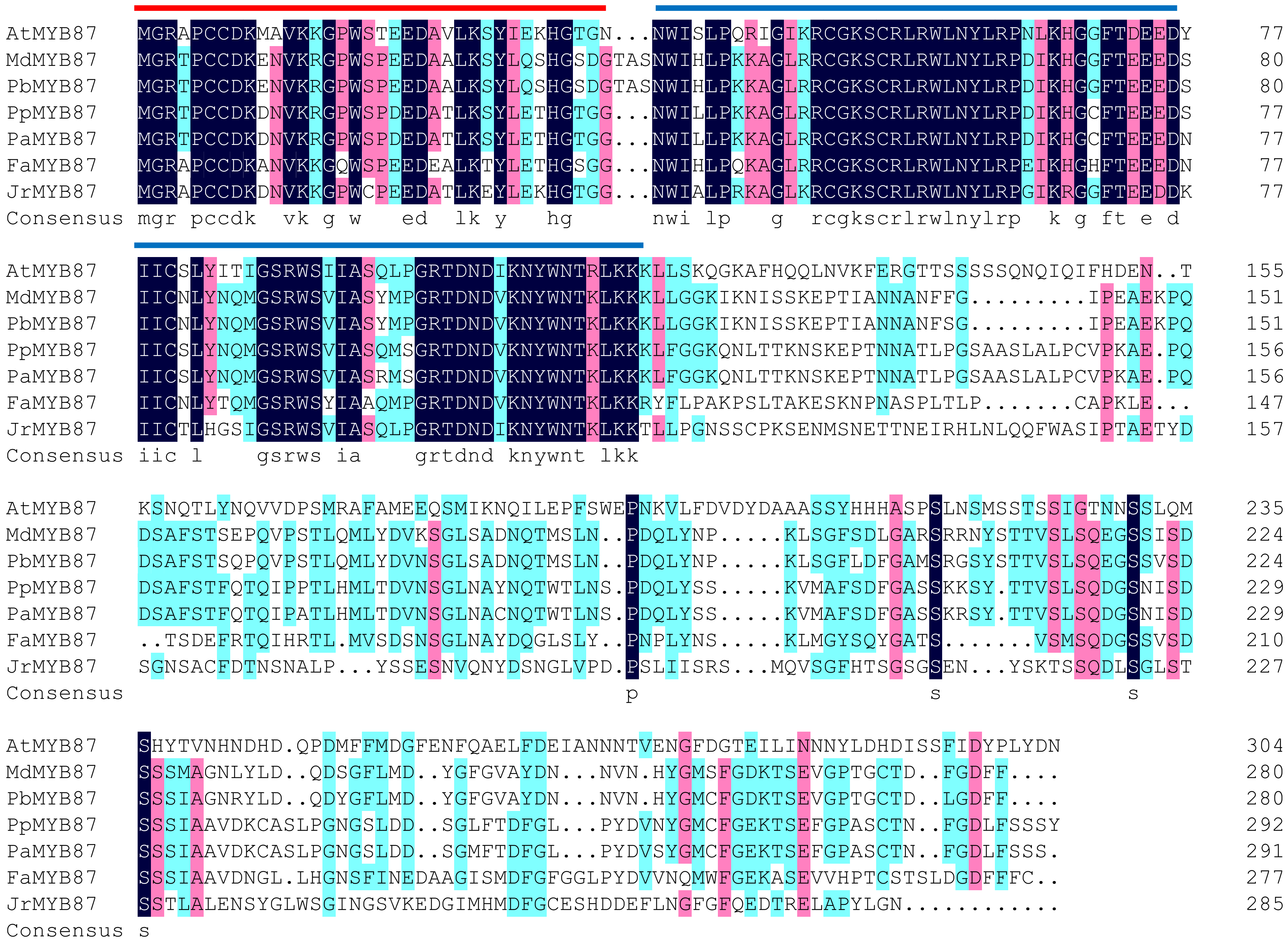
AtMYB87 MGRAPCCDKMAVKKGPWSTEEDAVLKSYIEKHGTGN...NWISLPQRIGIKRCGKSCRLRWLNYLRPNLKHGGFTDEEDY 77
MdMYB87 MGRTPCCDKENVKRGPWSPEEDAALKSYLQSHGSDGTASNWIHLPKKAGLRRCGKSCRLRWLNYLRPDIKHGGFTEEEDS 80
PbMYB87 MGRTPCCDKENVKRGPWSPEEDAALKSYLQSHGSDGTASNWIHLPKKAGLRRCGKSCRLRWLNYLRPDIKHGGFTEEEDS 80
PpMYB87 MGRTPCCDKDNVKRGPWSPDEDATLKSYLETHGTGG...NWILLPKKAGLRRCGKSCRLRWLNYLRPDIKHGCFTEEEDS 77
PaMYB87 MGRTPCCDKDNVKRGPWSPDEDATLKSYLETHGTGG...NWILLPKKAGLRRCGKSCRLRWLNYLRPDIKHGCFTEEEDN 77
FaMYB87 MGRAPCCDKANVKKGQWSPEEDEALKTYLETHGSGG...NWIHLPQKAGLRRCGKSCRLRWLNYLRPEIKHGHFTEEEDN 77
JrMYB87 MGRAPCCDKDNVKKGPWCPEEDATLKEYLEKHGTGG...NWIALPRKAGLKRCGKSCRLRWLNYLRPGIKRGGFTEEDDK 77
Consensus mgr pccdk vk g w ed lk y hg nwi lp g rcgkscrlrwlnylrp k g ft e d
AtMYB87 IICSLYITIGSRWSIIASQLPGRTDNDIKNYWNTRLKKKLLSKQGKAFHQQLNVKFERGTTSSSSSQNQIQIFHDEN..T 155
MdMYB87 IICNLYNQMGSRWSVIASYMPGRTDNDVKNYWNTKLKKKLLGGKIKNISSKEPTIANNANFFG.........IPEAEKPQ 151
PbMYB87 IICNLYNQMGSRWSVIASYMPGRTDNDVKNYWNTKLKKKLLGGKIKNISSKEPTIANNANFSG.........IPEAEKPQ 151
PpMYB87 IICSLYNQMGSRWSVIASQMSGRTDNDVKNYWNTKLKKKLFGGKQNLTTKNSKEPTNNATLPGSAASLALPCVPKAE.PQ 156
PaMYB87 IICSLYNQMGSRWSVIASRMSGRTDNDVKNYWNTKLKKKLFGGKQNLTTKNSKEPTNNATLPGSAASLALPCVPKAE.PQ 156
FaMYB87 IICNLYTQMGSRWSYIAAQMPGRTDNDVKNYWNTKLKKRYFLPAKPSLTAKESKNPNASPLTLP.......CAPKLE... 147
JrMYB87 IICTLHGSIGSRWSVIASQLPGRTDNDIKNYWNTKLKKTLLPGNSSCPKSENMSNETTNEIRHLNLQQFWASIPTAETYD 157
Consensus iic l gsrws ia grtdnd knywnt lkk
AtMYB87 KSNQTLYNQVVDPSMRAFAMEEQSMIKNQILEPFSWEPNKVLFDVDYDAAASSYHHHASPSLNSMSSTSSIGTNNSSLQM 235
MdMYB87 DSAFSTSEPQVPSTLQMLYDVKSGLSADNQTMSLN..PDQLYNP.....KLSGFSDLGARSRRNYSTTVSLSQEGSSISD 224
PbMYB87 DSAFSTSQPQVPSTLQMLYDVNSGLSADNQTMSLN..PDQLYNP.....KLSGFLDFGAMSRGSYSTTVSLSQEGSSVSD 224
PpMYB87 DSAFSTFQTQIPPTLHMLTDVNSGLNAYNQTWTLNS.PDQLYSS.....KVMAFSDFGASSKKSY.TTVSLSQDGSNISD 229
PaMYB87 DSAFSTFQTQIPATLHMLTDVNSGLNACNQTWTLNS.PDQLYSS.....KVMAFSDFGASSKRSY.TTVSLSQDGSNISD 229
FaMYB87 ..TSDEFRTQIHRTL.MVSDSNSGLNAYDQGLSLY..PNPLYNS.....KLMGYSQYGATS.......VSMSQDGSSVSD 210
JrMYB87 SGNSACFDTNSNALP...YSSESNVQNYDSNGLVPD.PSLIISRS...MQVSGFHTSGSGSEN...YSKTSSQDLSGLST 227
Consensus p s s
AtMYB87 SHYTVNHNDHD.QPDMFFMDGFENFQAELFDEIANNNTVENGFDGTEILINNNYLDHDISSFIDYPLYDN 304
MdMYB87 SSSMAGNLYLD..QDSGFLMD..YGFGVAYDN...NVN.HYGMSFGDKTSEVGPTGCTD..FGDFF.... 280
PbMYB87 SSSIAGNRYLD..QDYGFLMD..YGFGVAYDN...NVN.HYGMCFGDKTSEVGPTGCTD..LGDFF.... 280
PpMYB87 SSSIAAVDKCASLPGNGSLDD..SGLFTDFGL...PYDVNYGMCFGEKTSEFGPASCTN..FGDLFSSSY 292
PaMYB87 SSSIAAVDKCASLPGNGSLDD..SGMFTDFGL...PYDVSYGMCFGEKTSEFGPASCTN..FGDLFSSS. 291
FaMYB87 SSSIAAVDNGL.LHGNSFINEDAAGISMDFGFGGLPYDVVNQMWFGEKASEVVHPTCSTSLDGDFFFC.. 277
JrMYB87 SSTLALENSYGLWSGINGSVKEDGIMHMDFGCESHDDEFLNGFGFQEDTRELAPYLGN........... 285
Consensus s
