# Supplementary material for: The MsSCL28.5–MdMYB87 module enhances drought tolerance by promoting adventitious root development in apple rootstocks
Source: Hortic Res. 2026 Jun 2;13(11):uhag211. doi: 10.1093/hr/uhag211 (PMC13718307; doi:10.1093/hr/uhag211)
Supplement: Supplementary file 1 [file web_material_uhag211.zip › Supplemental Figure S10.pdf]

MdSCL28.5	ATGGCACTGAGCCGAAACTTCCTGCCTGAGGAGTACATGATCAATCATGAAAGGACTAATAAAATGAGTGTCCTTGATCTCATTACTCACTCAGCTGCCTCAGCCATGCCTTGCTATCCT	120
MsSCL28.5	ATGGCACTGAGCCGAAACTTCCTGCCTGAGGAGTACATGATCAATCATGAAAGGACTAATAAAATGAGTGTCCTTGATCTCATTACTCACTCAGCTGCCTCAGCCATGCCTTGCTATCCT	120
Consensus	atggcactgagccgaaacttcctgcctgaggagtacatgatcaatcatgaaaggactaataaaatgagtgtccttgatctcattactcactcagctgcctcagccatgccttgctatcct	
MdSCL28.5	TATCCACACACGCCCTTGCTGGAGGAGGGCGGTACAGCTTCTACCCCCCCTCACTTAGATGAAAGCGGAAACCATAAGAGGCTGAAAAGGACTACAAGTATCTCCGACTCTATGTCAAGT	240
MsSCL28.5	TATCCACACACGCCCTTGCTGGAGGAGGGCGGTACAGCTTCTACCCCCCCTCACTTAGATGAAAGCGGAAACCATAAGAGGCTGAAAAGGACTACAAGTATCTCCGACTCTATGTCAAGT	240
Consensus	tatccacacacgcccttgctggaggagggcggtacagcttctaccccccctcacttagatgaaagcggaaaccataagaggctgaaaaggactacaagtatctccgactctatgtcaagt	
MdSCL28.5	CATAACAGTCTTTATAGTGGTGGAGGAAGCGGAGATAGTTGCATTACTGACATCAGCCTCAGTCGCCGAAGCAGCACGAATAGCTTGAATACCTTGCCAAGGCTTCATTTCCGGGACCAT	360
MsSCL28.5	CATAACAGTCTTTATAGTGGTGGAGGAAGCGGAGATAGTTGCATTACTGACATCAGCCTCAGTCGCCGAAGCAGCACGAATAGCTTGAATACCTTGCCAAGGCTTCATTTCCGGGACCAT	360
Consensus	cataacagtctttatagtggtggaggaagcggagatagttgcattactgacatcagcctcagtcgccgaagcagcacgaatagcttgaataccttgccaaggcttcatttccgggaccat	
MdSCL28.5	ATTTGGACTTACACACAAAGGTACCTTGCAGCTGAAGCTGTTGAGGAGGCAGCGACAGTGATGTCCAATGCCGAGGGAAATGAAGAGGAGGAGGACGGAACTGCTGATGGGATGAGGCTT	480
MsSCL28.5	ATTTGGACTTACACACAAAGGTACCTTGCAGCTGAAGCTGTTGAGGAGGCAGCGACAGTGATGTCCAATGCCGAGGGAAATGAAGAGGAGGAGGACGGAACTGCTGATGGGATGAGGCTT	480
Consensus	atttggacttacacacaaaggtaccttgcagctgaagctgttgaggaggcagcgacagtgatgtccaatgccgagggaaatgaagaggaggaggacggaactgctgatgggatgaggctt	
MdSCL28.5	GTTCAGCTCCTAATTGCTTGTGCTGAAGCTGTTGCTTGTCGCGACAAGTCCCATGCCTCTGCGTTACTATCTGAGCTTCGGGCCAATGCCTTGGTCTTTGGCTCTTCATTCCAGCGTGTG	600
MsSCL28.5	GTTCAGCTCCTAATTGCTTGTGCTGAAGCTGTTGCTTGTCGCGACAAGTCCCATGCCTCTGCGTTACTATCTGAGCTTCGGGCCAATGCCTTGGTCTTTGGCTCTTCATTCCAGCGTGTG	600
Consensus	gttcagctcctaattgcttgtgctgaagctgttgcttgtcgcgacaagtcccatgcctctgcgttactatctgagcttcgggccaatgccttggtctttggctcttcattccagcgtgtg	
MdSCL28.5	GCATCATGTTTTGTCCAAGGCCTTGCCAACCGCCTAGCTCTGGTTCAACCGCTTGGGGCAGTAGGGTTCATTGGGTCCGCGATGAGCACAAAAGACTTTGCCTTGGACAAGAAGGAAGAG	720
MsSCL28.5	GCATCATGTTTTGTCCAAGGCCTTGCCAACCGCCTAGCTCTGGTTCAACCGCTTGGGGCAGTAGGGTTCATTGGGTCCGCGATGAGCACAAAAGACTTTGCCTTGGACAAGAAGGAAGAG	720
Consensus	gcatcatgttttgtccaaggccttgccaaccgcctagctctggttcaaccgcttggggcagtagggttcattgggtccgcgatgagcacaaaagactttgccttggacaagaaggaagag	
MdSCL28.5	GCATTGCGCCTTGTTTATGAGATATGCCCCCACATTCAGTTTGGTCACTTTGTGGCCAATTCATCAATATTGGAAGCCTTTGAGGGAGAGAGTTTTGTTCATGTAGTAGACCTTGGGATG	840
MsSCL28.5	GCATTGCGCCTTGTTTATGAGATATGCCCCCACATTCAGTTTGGTCACTTTGTGGCCAATTCATCAATATTGGAAGCCTTTGAGGGAGAGAGTTTTGTTCATGTAGTAGACCTTGGGATG	840
Consensus	gcattgcgccttgtttatgagatatgcccccacattcagtttggtcactttgtggccaattcatcaatattggaagcctttgagggagagagttttgttcatgtagtagaccttgggatg	
MdSCL28.5	ACCCTTGGTCTACCTCATGGAGACCAGTGGCAAGGCTTGATCGAGAGCCTTGCCACACGCACAGGCCAGCCGCCTACACGCCTTAGAATAACCGGTGTTGGCCTTTACGGTGACAGATTG	960
MsSCL28.5	ACCCTTGGTCTACCTCATGGAGACCAGTGGCAAGGCTTGATCGAGAGCCTTGCCACACGCACAGGCCAGCCGCCTACACGCCTTAGAATAACCGGTGTTGGCCTTTACGGTGACAGATTG	960
Consensus	acccttggtctacctcatggagaccagtggcaaggcttgatcgagagccttgccacacgcacaggccagccgcctacacgccttagaataaccggtgttggcctttacggtgacagattg	
MdSCL28.5	CAAATCATTGGAGAAGAGCTTGAGGCCTATGCCGACAGCTTGGGAATAAACCTGGAGTTTTCTGTGGTGGAAAGCAACTTGGAAAACCTGCGTCCGGAGGACATTAAAATGTATGATGGT	1080
MsSCL28.5	CAAATCATTGGAGAAGAGCTTGAGGCCTATGCCGACAGCTTGGGAATAAACCTGGAGTTTTCTGTGGTGGAAAGCAACTTGGAAAACCTGCGTCCGGAGGACATTAAAATGTATGATGGT	1080
Consensus	caaatcattggagaagagcttgaggcctatgccgacagcttgggaataaacctggagttttctgtggtggaaagcaacttggaaaacctgcgtccggaggacattaaaatgtatgatggt	
MdSCL28.5	GAAGTCCTTGTTGTCAATAGCATTCTTCAGTTGCATTGTGTGGTGAAAGAAAGCAGAGGAGCTCTCAATTCTGTCCTGCAGATGGTCCATGAGCTTTCGCCGAAAATCTTGGTTCTTGTG	1200
MsSCL28.5	GAAGTCCTTGTTGTCAATAGCATTCTTCAGTTGCATTGTGTGGTGAAAGAAAGCAGAGGAGCTCTCAATTCTGTCCTGCAGATGGTCCATGAGCTTTCGCCGAAAATCTTGGTTCTTGTG	1200
Consensus	gaagtccttgttgtcaatagcattcttcagttgcattgtgtggtgaaagaaagcagaggagctctcaattctgtcctgcagatggtccatgagctttcgccgaaaatcttggttcttgtg	
MdSCL28.5	GAGCAAGACTCAAGCCACAATGGACCTTTTTTTTTGGGGAGGTTTATGGAGGCACTGCATTATTACTCTGCAATCTTTGACTCCCTAGATGCCATGCTGCCAAAATATGACACGAGGCGC	1320
MsSCL28.5	GAGCAAGACTCAAGCCACAATGGACCTTTTTTTTTGGGGAGGTTTATGGAGGCACTGCATTATTACTCTGCAATCTTTGACTCCCTAGATGCCATGCTGCCAAAATATGACACGAGGCGC	1320
Consensus	gagcaagactcaagccacaatggaccttttttttttggggaggtttatggaggcactgcattattactctgcaatctttgactccctagatgccatgctgccaaaatatgacacgaggcgc	
MdSCL28.5	GCGAAGATGGAGCAGTTCTACTTTGCTGAGGAGATTAAGAACATAATCAGCTGCGAGGGGCCGGCAAGGGTGGAGAGGCACGAGAGGGTTGATCAGTGGCGCCGGAGGATGAGCCGGGCA	1440
MsSCL28.5	GCGAAGATGGAGCAGTTCTACTTTGCTGAGGAGATTAAGAACATAATCAGCTGCGAGGGGCCGGCAAGGGTGGAGAGGCACGAGAGGGTTGATCAGTGGCGCCGGAGGATGAGCCGGGCA	1440
Consensus	gcgaagatggagcagttctactttgctgaggagattaagaacataatcagctgcgaggggccggcaagggtggagaggcacgagagggttgatcagtggcgccggaggatgagccgggca	
MdSCL28.5	GGGTTTCAGGCTGCGCCGATTAAGATGCTGGCGCAGGCAAAGCAGTGGCTTGGGAAGATCAAGGTCTGTGAGGGTTACACTATTTTGGAAGAGAAGGGTTGCCTGGTTCTTGGTTGGAAA	1560
MsSCL28.5	GGGTTTCAGGCTGCGCCGATTAAGATGCTGGCGCAGGCAAAGCAGTGGCTTGGGAAGATCAAGGTCTGTGAGGGTTACACTATTTTGGAAGAGAAGGGTTGCCTGGTTCTTGGTTGGAAA	1560
Consensus	gggtttcaggctgcgccgattaagatgctggcgcaggcaaagcagtggcttgggaagatcaaggtctgtgagggttacactattttggaagagaagggttgcctggttcttggttggaaa	
MdSCL28.5	TCTAAGCCCATTGTTGCTGCCTCCTGCTGGAAATGCTG	1598
MsSCL28.5	TCTAAGCCCATTGTTGCTGCCTCCTGCTGGAAATGCTG	1598
Consensus	tctaagcccattgttgctgcctcctgctggaaatgctg	
