# Supplementary material for: The MsSCL28.5–MdMYB87 module enhances drought tolerance by promoting adventitious root development in apple rootstocks
Source: Hortic Res. 2026 Jun 2;13(11):uhag211. doi: 10.1093/hr/uhag211 (PMC13718307; doi:10.1093/hr/uhag211)
Supplement: Supplementary file 1 [file web_material_uhag211.zip › Supplemental Figure S11.pdf]

```
MdMYB87   ATGGGACGCACTCCATGCTGTGACAAAGAGAACGTGAAGAGAGGTCCGTGGTCTCCGGAAGAAGACGCAGCTCTCAAGTCCTACCTCCAGTCTCATGGCAGTGATGGCACCGCCAGCAAT   120
MsMYB87   ATGGGACGCACTCCATGCTGTGACAAAGAGAACGTGAAGAGAGGTCCGTGGTCTCCGGAAGAAGACGCAGCTCTCAAGTCCTACCTCCAGTCTCATGGCAGTGATGGCACCGCCAGCAAT   120
Consensus atgggacgcactccatgctgtgacaaagagaacgtgaagagaggtccgtggtctccggaagaagacgcagctctcaagtcctacctccagtctcatggcagtgatggcaccgccagcaat

MdMYB87   TGGATTCATCTCCCCAAAAAAGCTGGCCTGAGGCGGTGCGGGAAGAGCTGCCGTCTGCGGTGGCTGAACTATCTGAGGCCAGACATCAAACACGGTGGCTTCACTGAGGAGGAGGACAGC   240
MsMYB87   TGGATTCATCTCCCCAAAAAAGCTGGCCTGAGGCGGTGCGGGAAGAGCTGCCGTCTGCGGTGGCTGAACTATCTGAGGCCAGACATCAAACACGGTGGCTTCACTGAGGAGGAGGACAGC   240
Consensus tggattcatctccccaaaaaagctggcctgaggcggtgcgggaagagctgccgtctgcggtggctgaactatctgaggccagacatcaaacacggtggcttcactgaggaggaggacagc

MdMYB87   ATTATCTGCAACCTCTACAACCAGATGGGAAGCCGATGGTCAGTCATTGCTTCTTACATGCCTGGAAGAACAGACAACGATGTGAAGAACTACTGGAACACCAAATTAAAGAAGAAGCTT   360
MsMYB87   ATTATCTGCAACCTCTACAACCAGATGGGAAGCCGATGGTCAGTCATTGCTTCTTACATGCCTGGAAGAACAGACAACGATGTGAAGAACTACTGGAACACCAAATTAAAGAAGAAGCTT   360
Consensus attatctgcaacctctacaaccagatgggaagccgatggtcagtcattgcttcttacatgcctggaagaacagacaacgatgtgaagaactactggaacaccaaattaaagaagaagctt

MdMYB87   TTGGGAGGAAAGATAAAGAATATCAGCAGCAAAGAGCCCACAATTGCAAACAATGCCAATTTTTTTGGAATCCCAGAAGCCGAGAAACCCCAAGACTCTGCCTTTTCGACGTCCGAACCC   480
MsMYB87   TTGGGAGGAAAGATAAAGAATATCAGCAGCAAAGAGCCCACAATTGCAAACAATGCCAATTTTTTTGGAATCCCAGAAGCCGAGAAACCCCAAGACTCTGCCTTTTCGACGTCCGAACCC   480
Consensus ttgggaggaaagataaagaatatcagcagcaaagagcccacaattgcaaacaatgccaatttttttggaatcccagaagccgagaaaccccaagactctgccttttcgacgtccgaaccc

MdMYB87   CAAGTTCCATCCACATTGCAGATGCTATATGATGTGAATTCTGGACTTAGTGCAGATAACCAGACCATGAGCTTAAACCCTGATCAGTTATACAACCCCAAGCTCTCGGGTTTTTCGGAT   600
MsMYB87   CAAGTTCCATCCACATTGCAGATGCTATATGATGTGAATTCTGGACTTAGTGCAGATAACCAGACCATGAGCTTAAACCCTGATCAGTTATACAACCCCAAGCTCTCGGGTTTTTCGGAT   600
Consensus caagttccatccacattgcagatgctatatgatgtgaattctggacttagtgcagataaccagaccatgagcttaaaccctgatcagttatacaaccccaagctctcgggtttttcggat

MdMYB87   TTGGGTGCAAGGTCAAGGAGGAATTACAGCACAACTGTTTCATTGTCTCAGGAGGGATCAAGCATTTCGGATACGTCTTCCATGGCTGGGAATCTTTATCTTGATCAGGATTCTGGATTT   720
MsMYB87   TTGGGTGCAAGGTCAAGGAGGAATTACAGCACAACTGTTTCATTGTCTCAGGAGGGATCAAGCATTTCGGATTCGTCTTCCATGGCTGGGAATCTTTATCTTGATCAGGATTCTGGATTT   720
Consensus ttgggtgcaaggtcaaggaggaattacagcacaactgtttcattgtctcaggagggatcaagcatttcggat cgtcttccatggctgggaatctttatcttgatcaggattctggattt

MdMYB87   TTGATGGATTATGGATTTGGTGTGGCTTATGATAATAATGTCAACCATTATGGCATGTCCTTTGGAGACAAAACCAGTGAAGTTGGTCCAACTGGATGCACAGATTTTGGTGATTTCTTT   840
MsMYB87   TTGATGGATTATGGATTTGGTGTGGCTTATGATAATAATGTCAACCATTATGGCATGTCCTTTGGAGACAAAACCAGTGAAGTTGGTCCAACTGGATGCACAGATTTTGGTGATTTCTTT   840
Consensus ttgatggattatggatttggtgtggcttatgataataatgtcaaccattatggcatgtcctttggagacaaaaccagtgaagttggtccaactggatgcacagattttggtgatttcttt

MdMYB87   TG   842
MsMYB87   TG   842
Consensus tg
```
