# Supplementary material for: The MsSCL28.5–MdMYB87 module enhances drought tolerance by promoting adventitious root development in apple rootstocks
Source: Hortic Res. 2026 Jun 2;13(11):uhag211. doi: 10.1093/hr/uhag211 (PMC13718307; doi:10.1093/hr/uhag211)
Supplement: Supplementary file 1 [file web_material_uhag211.zip › Supplemental Figure S12.pdf]

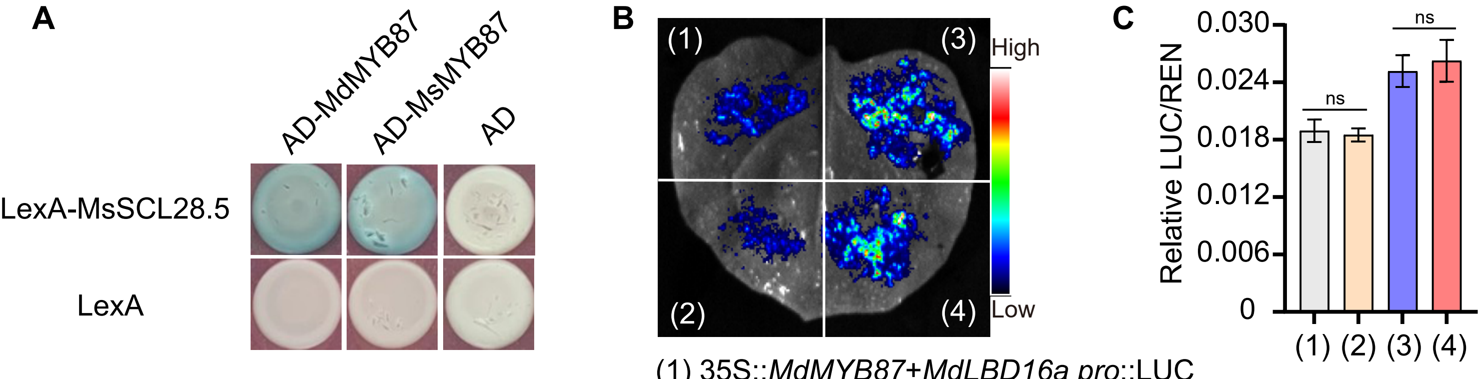

(1) 35S::*MdMYB87*+*MdLBD16a pro*::LUC
(2) 35S::*MsMYB87*+*MdLBD16a pro*::LUC
(3) 35S::*MsSCL28.5*+35S::*MdMYB87*+*MdLBD16a pro*::LUC
(4) 35S::*MsSCL28.5*+35S::*MsMYB87*+*MdLBD16a pro*::LUC
