# Supplementary material for: The MsSCL28.5–MdMYB87 module enhances drought tolerance by promoting adventitious root development in apple rootstocks
Source: Hortic Res. 2026 Jun 2;13(11):uhag211. doi: 10.1093/hr/uhag211 (PMC13718307; doi:10.1093/hr/uhag211)
Supplement: Supplementary file 1 [file web_material_uhag211.zip › Supplemental Figure S13.pdf]

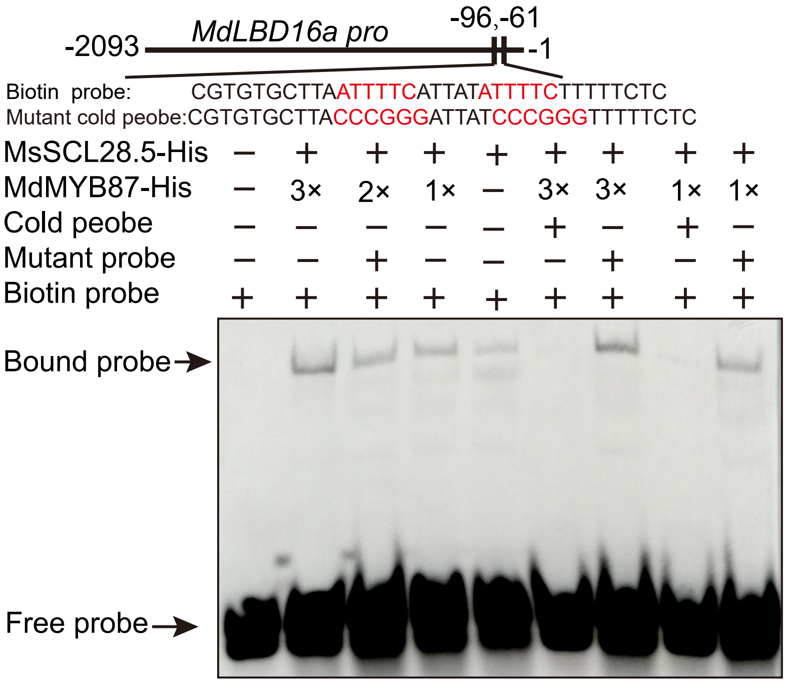

-96,-61
MdLBD16a pro
-2093
-1
Biotin probe: CGTGTGCTTAATTTTCATTATATTTTCTTTTTCTC
Mutant cold peobe:CGTGTGCTTACCCGGGATTATCCCGGGTTTTTCTC
MsSCL28.5-His − + + + + + + + +
MdMYB87-His − 3× 2× 1× − 3× 3× 1× 1×
Cold peobe − − − − − + − + −
Mutant probe − − + − − − + − +
Biotin probe + + + + + + + + +
Bound probe
Free probe
