# Supplementary material for: The MsSCL28.5–MdMYB87 module enhances drought tolerance by promoting adventitious root development in apple rootstocks
Source: Hortic Res. 2026 Jun 2;13(11):uhag211. doi: 10.1093/hr/uhag211 (PMC13718307; doi:10.1093/hr/uhag211)
Supplement: Supplementary file 1 [file web_material_uhag211.zip › Supplemental Figure S15.pdf]

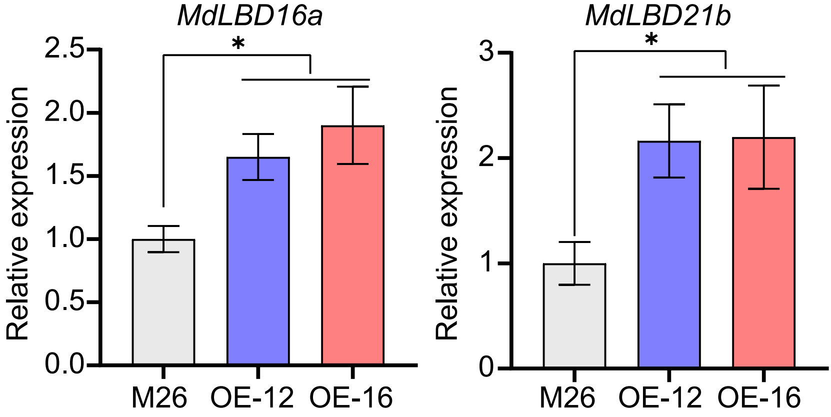

MdLBD16a
MdLBD21b
Relative expression
*
2.5
2.0
1.5
1.0
0.5
0.0
3
2
1
0
M26
OE-12
OE-16
