# Supplementary material for: The MsSCL28.5–MdMYB87 module enhances drought tolerance by promoting adventitious root development in apple rootstocks
Source: Hortic Res. 2026 Jun 2;13(11):uhag211. doi: 10.1093/hr/uhag211 (PMC13718307; doi:10.1093/hr/uhag211)
Supplement: Supplementary file 1 [file web_material_uhag211.zip › Supplemental Figure S16.pdf]

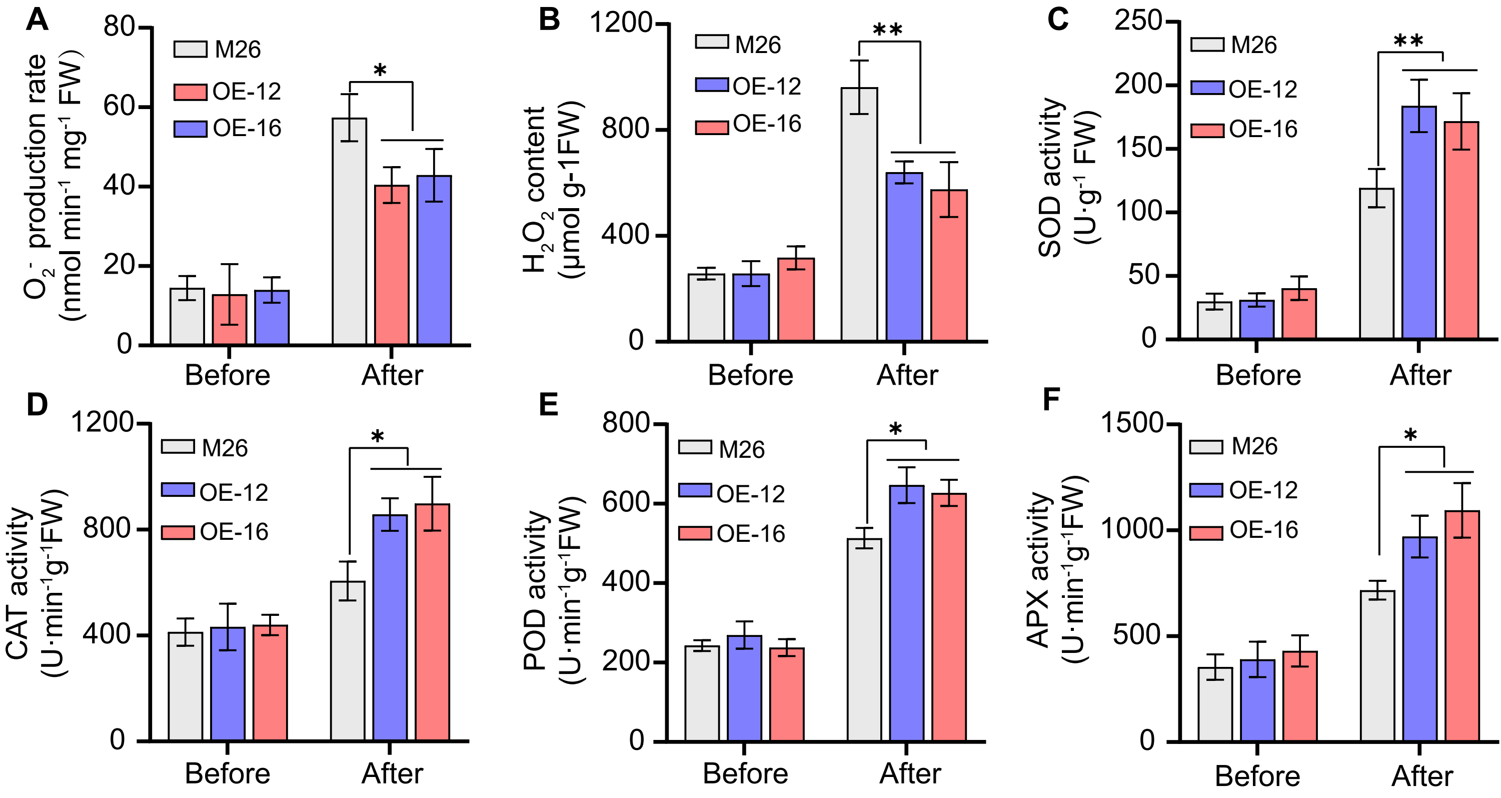

A
O_2^- production rate (nmol min^{-1} mg^{-1} FW)
M26
OE-12
OE-16
*
Before
After
B
H_2O_2 content (μmol g-1FW)
M26
OE-12
OE-16
**
Before
After
C
SOD activity (U·g^{-1} FW)
M26
OE-12
OE-16
**
Before
After
D
CAT activity (U·min^{-1}g^{-1}FW)
M26
OE-12
OE-16
*
Before
After
E
POD activity (U·min^{-1}g^{-1}FW)
M26
OE-12
OE-16
*
Before
After
F
APX activity (U·min^{-1}g^{-1}FW)
M26
OE-12
OE-16
*
Before
After
