# Supplementary material for: The MsSCL28.5–MdMYB87 module enhances drought tolerance by promoting adventitious root development in apple rootstocks
Source: Hortic Res. 2026 Jun 2;13(11):uhag211. doi: 10.1093/hr/uhag211 (PMC13718307; doi:10.1093/hr/uhag211)
Supplement: Supplementary file 1 [file web_material_uhag211.zip › Supplemental Figure S17.pdf]

```
MdProSCL28.5.seq  CAACAACAACAACAACAACCATCAAGCCTTATTCCACTAAATGGGATTGGCTGTATAAATTCTAGAACGCTACTGCGCTGGGTTTTGCTCAAGTCTTTTGTCAGCTCCAAGTAAATTTTCTTTCCAAGTCTTCATGGGTCTTCACATACCCATTTTGCCC  160
MsProSCL28.5.seq  CAACAACAACAACAACAACCATCAAGCCTTATTCCACTAAATGGGATTGGCTGTATAAATTCTAGAACGCTACTGCGCTGGGTTTTGCTCAAGTCTTTTGTCAGCTCCAAGTAAATTTTCTTTCCAAGTCTTCATGGGTCTTCACATACCCATTTTGCCC  160
Consensus         caacaacaacaacaacaaccatcaagccttattccactaaatgggattggctgtataaattctagaacgctactgcgctgggttttgctcaagtcttttgtcagctccaagtaaattttctttccaagtcttcatgggtcttcacatacccattttgccc

MdProSCL28.5.seq  TGAGCCTCAGTCTTATAGTCGCATCTTCTAACCGGAGTATTTGTAAGTCTTCGGTTCACGTGTCCAAACCACCTTAATCGATTTTTTCTCATTTTATCTTCAATTACGGCAACTCGTACTTTATCTTGAATATCCTCTCGTTCTTAAATAACCTTCCTGC  320
MsProSCL28.5.seq  TGAGCCTCAGTCTTATAGTCGCATCTTCTAACCGGAGTATTTGTAAGTCTTCGGTTCACGTGTCCAAACCACCTTAATCGATTTTTTCTCATTTTATCTTCAATTACGGCAATTCGTACTTTATCTTGAATATCCTCTCGTTCTTAAATAACCTTCCAGC  320
Consensus         tgagcctcagtcttatagtcgcatcttctaaccggagtatttgtaagtcttcggttcacgtgtccaaaccaccttaatcgattttttctcattttatcttcaattacggcaa tcgtactttatcttgaatatcctctcgttcttaaataaccttcc gc

MdProSCL28.5.seq  TACATTTGCACAAAAGCTTGATCTATACAGCAGCTTCCCCTTATTGACCAGGCAACATACATACGTGCTAGCTGTTTTCAGTTTCTTGTTGCAAAATATCAAGCCCTTAGAATATGCTACACTGATAGTATCGCTCGATGACGCAAGTTGTTATTATAAA  480
MsProSCL28.5.seq  TACATTTGCACAAAAGCTTGATCTATACAGCAGCTTCCCCTTATTGACCAGGCAACATACATACGTGCTAGCTGTTTTCAGTTTCTTGTTGCAAAATATCAAGCCCTTAGAATATGCTACACTGATAGTATCGCTCGATGACGCAAGTTGTTATTATAAA  480
Consensus         tacatttgcacaaaagcttgatctatacagcagcttccccttattgaccaggcaacatacatacgtgctagctgttttcagtttcttgttgcaaaatatcaagcccttagaatatgctacactgatagtatcgctcgatgacgcaagttgttattataaa

MdProSCL28.5.seq  AATGAAGAAAAATCCACAAATTATTTGTTCTATAATCGGAACATAATACAAGGATGCAACATATAGAAACATAGAGGGCTCTATTGCGTGTCAATGCACCTCCATTTTTACATGCATCTGCTTGTTTTTTCTCATTGTGTTCATGAAAGCCGGCAAAAGA  640
MsProSCL28.5.seq  AATGAAGAAAAATCCACAAATTATTTGTTCTATAATCGGAACATAATACAAGGATGCAACATATAGAAACATAGAGGGCTCTATTGCGTGTCAATGCACCTCCATTTTTACATGCATCTGCTTGTTTTTTCTCATTGTGTTCATGAAAGCCGGCAAAAGA  640
Consensus         aatgaagaaaaatccacaaattatttgttctataatcggaacataatacaaggatgcaacatatagaaacatagagggctctattgcgtgtcaatgcacctccatttttacatgcatctgcttgttttttctcattgtgttcatgaaagccggcaaaaga

MdProSCL28.5.seq  ATGATTTAAAGAAAAATGCATGGGGGGAGGCAGCTGGTCTGGTCTGCATGCTACAAAGTTCCACAATCCTCATTGAATTGTTCGTAATTGAGTACGTAAATACGTGTAAGGGAAATGGAAATTCGAATCGGTGAATCGAAGACGATGAGAGTTGGAATGT  800
MsProSCL28.5.seq  ATGATTTAAAGAAAAATGCATGGGGGGAGGCAGCTGGTCTGGTCTGCATGCTACAAAGTTCCACAATCCTCATTGAATTGTTCGTAATTGAGTACGTAAATACGTGTAAGGGAAATGGAAATTCGAATCGGTGAATCGAAGACGATGAGAGTTGGAATGT  800
Consensus         atgatttaaagaaaaatgcatggggggaggcagctggtctggtctgcatgctacaaagttccacaatcctcattgaattgttcgtaattgagtacgtaaatacgtgtaagggaaatggaaattcgaatcggtgaatcgaagacgatgagagttggaatgt

MdProSCL28.5.seq  ACAATCGATGACAATGAAACTTAGAAAAAAGCAGCACTCTAGGAATCATCAAAACCTAAGTACTGAAAAGGGCAGTAAGAAACAACCTTGTTTTCCGCAATAAAGAAACCTTAGTTTACCCAAAAACATTGGAGAGGTGTAAAGCCGATTGGAGCACTTC  960
MsProSCL28.5.seq  ACAATCGATGACAATGAAACTTAGAAAAAAGCAGCACTCTAGGAATCATCAAAACCTAAGTACTGAAAAGGGCAGTAAGAAACAACCTTGTTTTCCGCAATAAAGAAACCTTAGTTTACCCAAAAACATTGGAGAGGTGTAAAGCCGATTGGAGCACTTC  960
Consensus         acaatcgatgacaatgaaacttagaaaaaagcagcactctaggaatcatcaaaacctaagtactgaaaagggcagtaagaaacaaccttgttttccgcaataaagaaaccttagtttacccaaaaacattggagaggtgtaaagccgattggagcacttc

MdProSCL28.5.seq  CTCACCAACATGGAATCTAGATTGATGTATTTGAAATGACTCACAAAACAACTTGTTAAAGGATGTCTCGCATCGGAAACGTGAGGAACATCCCTCTTATATTGAATGGTTCTACTAATTGCATCGAGACTTTTTGTATAAAACTCGGTCCGCACCTACT  1120
MsProSCL28.5.seq  CTCACCAACATGGAATCTAGATTGATGTATTTGAAATGACTCACAAAACAACTTGTTAAAGGATGTCTCGCATCGGAAACGTGAGGAACATCCCTCTTATATTGAATGGTTCTACTAATTGCATCGAGACTTTTTGTATAAAACTCGGTCCGCACCTACT  1120
Consensus         ctcaccaacatggaatctagattgatgtatttgaaatgactcacaaaacaacttgttaaaggatgtctcgcatcggaaacgtgaggaacatccctcttatattgaatggttctactaattgcatcgagactttttgtataaaactcggtccgcacctact

MdProSCL28.5.seq  AAATTATACAGGTGGTCAAGTTAAGGTCATTATTGATGTGCTTAGTAGTGGACCGTTGGATCGTCTCTTTAACTTTAAGACAGCTTATCTGTTGAGGGGTAAGAAAGATTAAAAGAGGTTCAATGGGCAGGAGGCCCTATAGAATAAGACAACGCAGAAA  1280
MsProSCL28.5.seq  AAATTATACAGGTGGTCAAGTTAAGGTCATTATTGATGTGCTTAGTAGTGGACCGTTGGATCGTCTCTTTAACTTTAAGACAGCTTATCTGTTGAGGGGTAAGAAAGATTAAAAGAGGTTCAATGGGCAGGAGGCCCTATAGAATAAGACAACGCAGAAA  1280
Consensus         aaattatacaggtggtcaagttaaggtcattattgatgtgcttagtagtggaccgttggatcgtctctttaactttaagacagcttatctgttgaggggtaagaaagattaaaagaggttcaatgggcaggaggccctatagaataagacaacgcagaaa

MdProSCL28.5.seq  ATGACATAAACCTTAATAGGTGATTCGATTATAGATAAATTACCTCCTAGGAGATGACAAATGTAAAATCCAAGATTATTCTAATGTGACATAGGCAAGCCTTGTCCTTGGAGATGTCTATGCCGTAAAGTTTATTGATGACGTTTCTCATGATGTTTTG  1440
MsProSCL28.5.seq  ATGACATAAACCTTAATAGGTGATTCGATTATAGATAAATTACCTCCTAGGAGAGGACAAATGTAAAATCCAAGATTATTCTAATGTGACATAGGCAAGCCTTGTCCTTGGAGATGTCTATGCCGTAAAGTTTATTGATGACGTTTCTCATGATGTTTTG  1440
Consensus         atgacataaaccttaataggtgattcgattatagataaattacctcctaggaga gacaaatgtaaaatccaagattattctaatgtgacataggcaagccttgtccttggagatgtctatgccgtaaagtttattgatgacgtttctcatgatgttttg

MdProSCL28.5.seq  TGCAGGTACACACCATCTGTCCAAACTCTTCCCTTCTTTCGAGTTTTGTAATAATCTCCCTTGTTATCTCCAAATAACAAGGAAAGCTGGCTATTTAAGTCCTTGCTGCTTGGGATTAGGGTCTTTCAGAGCTTTGTTGAATATATTTTGAGAAAAAGAG  1600
MsProSCL28.5.seq  TGCAGGTACACACCATCTGTCCAAACTCTTCCCTTCTTTCGAGTTTTGTAATAATCTCCCTTGTTATCTCCAAATAACAAGGAAAGCTGGCTATTTAAGTCCTTGCTGCTTGGGATTAGGGTCTTTCAGAGCTTTGTTGAATATATTTTGAGAAAAAGAG  1600
Consensus         tgcaggtacacaccatctgtccaaactcttcccttctttcgagttttgtaataatctcccttgttatctccaaataacaaggaaagctggctatttaagtccttgctgcttgggattagggtctttcagagctttgttgaatatattttgagaaaaagag

MdProSCL28.5.seq  GAGGGAGAAAAGAAGGTTACGTTCGAGAAATGGCACTGAGCCGAAACTTCCTGCCTGAGGAGTACATGATCAATCATGAAAGGACTAATAAAATGAGTGTCCTTGATCTCATTACTCACTCAGCTGCCT  1729
MsProSCL28.5.seq  GAGGGAGAAAAGAAGGTTACGTTCGAGAAATGGCACTGAGCCGAAACTTCCTGCCTGAGGAGTACATGATCAATCATGAAAGGACTAATAAAATGAGTGTCCTTGATCTCATTACTCACTCAGCTGCCT  1729
Consensus         gagggagaaaagaaggttacgttcgagaaatggcactgagccgaaacttcctgcctgaggagtacatgatcaatcatgaaaggactaataaaatgagtgtccttgatctcattactcactcagctgcct
```
