# Supplementary material for: The MsSCL28.5–MdMYB87 module enhances drought tolerance by promoting adventitious root development in apple rootstocks
Source: Hortic Res. 2026 Jun 2;13(11):uhag211. doi: 10.1093/hr/uhag211 (PMC13718307; doi:10.1093/hr/uhag211)
Supplement: Supplementary file 1 [file web_material_uhag211.zip › Supplemental Figure S18.pdf]

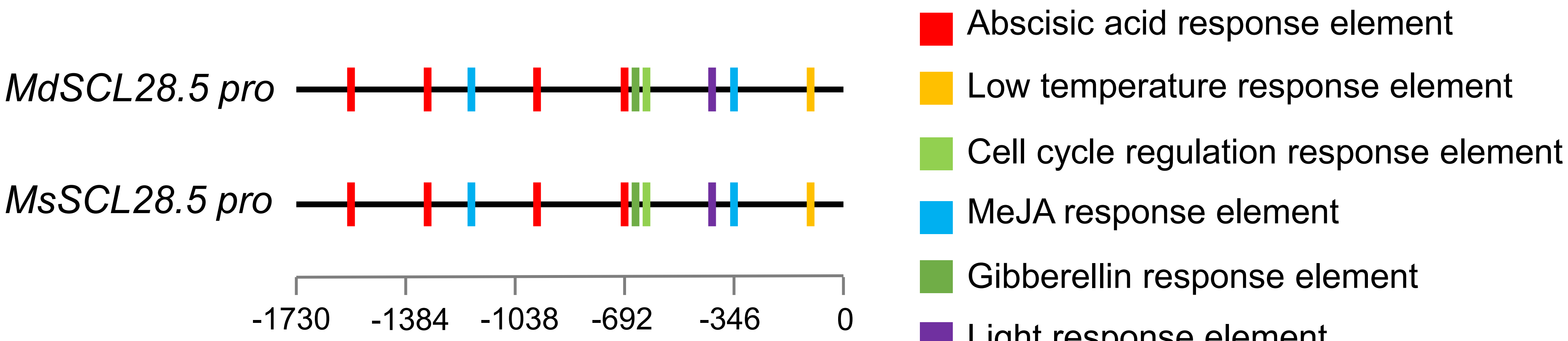
MdSCL28.5 pro
MsSCL28.5 pro
-1730
-1384
-1038
-692
-346
0
Abscisic acid response element
Low temperature response element
Cell cycle regulation response element
MeJA response element
Gibberellin response element
Light response element
