# Supplementary material for: The MsSCL28.5–MdMYB87 module enhances drought tolerance by promoting adventitious root development in apple rootstocks
Source: Hortic Res. 2026 Jun 2;13(11):uhag211. doi: 10.1093/hr/uhag211 (PMC13718307; doi:10.1093/hr/uhag211)
Supplement: Supplementary file 1 [file web_material_uhag211.zip › Supplemental Figure S19.pdf]

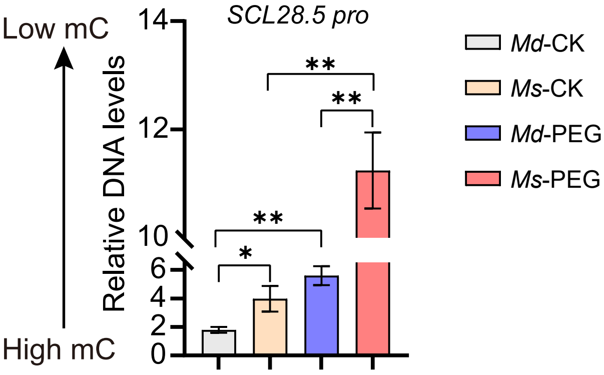

SCL28.5 pro
Low mC
High mC
Relative DNA levels
14
12
10
6
4
2
0
**
**
**
*
Md-CK
Ms-CK
Md-PEG
Ms-PEG
