# Supplementary material for: The MsSCL28.5–MdMYB87 module enhances drought tolerance by promoting adventitious root development in apple rootstocks
Source: Hortic Res. 2026 Jun 2;13(11):uhag211. doi: 10.1093/hr/uhag211 (PMC13718307; doi:10.1093/hr/uhag211)
Supplement: Supplementary file 1 [file web_material_uhag211.zip › Supplemental Figure S20.pdf]

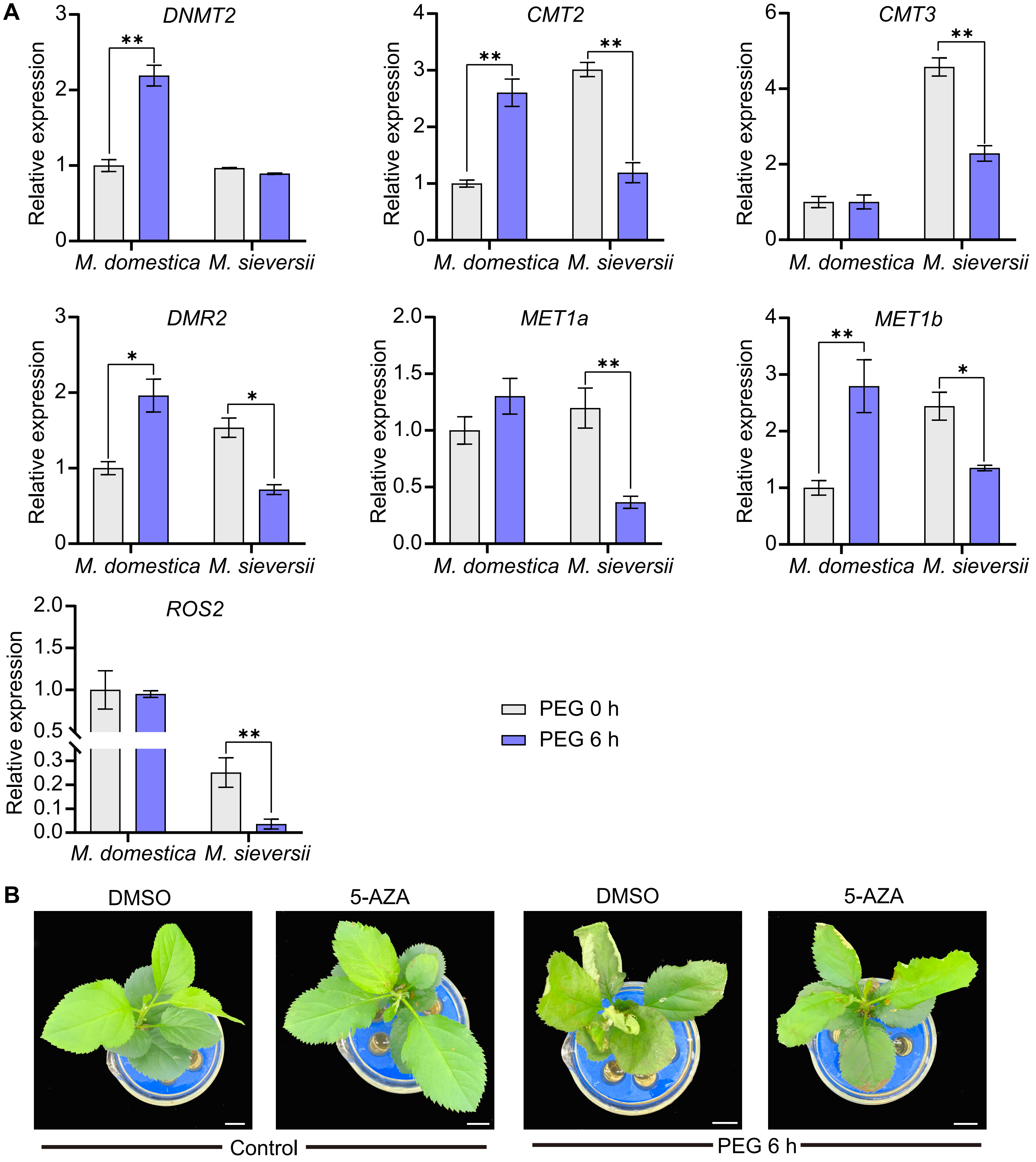

A
DNMT2
CMT2
CMT3
DMR2
MET1a
MET1b
ROS2
Relative expression
M. domestica
M. sieversii
PEG 0 h
PEG 6 h
B
DMSO
5-AZA
DMSO
5-AZA
Control
PEG 6 h
