# Supplementary material for: The MsSCL28.5–MdMYB87 module enhances drought tolerance by promoting adventitious root development in apple rootstocks
Source: Hortic Res. 2026 Jun 2;13(11):uhag211. doi: 10.1093/hr/uhag211 (PMC13718307; doi:10.1093/hr/uhag211)
Supplement: Supplementary file 1 [file web_material_uhag211.zip › Supplemental Figure S21.pdf]

```
ProMdMYB87.seq  ATGGACATACCCTAATGCAATCCAAAACTTAACCATATCGTAGTGCTTCCAAATATATATATATATATATATCTATAT..CTATATCTATATCTATATCTATATCTATATATATACATCAATAATGTGGCGTAAAATGCACGATCTATGACGCCCTACTC  158
ProMsMYB87.seq  ATGGACATACCCTAATGCAATCCAAAACTTAACCATATTGTAGTGCTTCCAAATATATATATATATATATATATATATATCTATATCTATATCTATATCTATATCTATATATATACATCAATAATGTGGCGTAAAATGCACGATCTATGACGCCCTACTC  160
Consensus       atggacataccctaatgcaatccaaaacttaaccatat gtagtgcttccaaatatatatatatatatatat tatat  ctatatctatatctatatctatatctatatatatacatcaataatgtggcgtaaaatgcacgatctatgacgccctactc

ProMdMYB87.seq  CCTAACTATTAATTTTCGGAGATAATTATCGGTGACAAGCTCAACAAAACACTAGTTTAACTAACTATCAATACTTACAATTCTTTACTTCAATTTCTCAATCCTTCACCCAATGATTTATGACCAACATCCATTCATTAAATCATAAGGTTAAATCTCA  318
ProMsMYB87.seq  CCTAACTATTAATTTTCGGAGATAATTATCGGTGACAAGCTCAACAAAACACTAGTTTAACTAACTATCAATACTTACAATTCTTTACTTCAATTTCTCAATCCTTCACCCAATGATTTATGACCAACATCCATTCATTAAATCATAAGGTTAAATCTCA  320
Consensus       cctaactattaattttcggagataattatcggtgacaagctcaacaaaacactagtttaactaactatcaatacttacaattctttacttcaatttctcaatccttcacccaatgatttatgaccaacatccattcattaaatcataaggttaaatctca

ProMdMYB87.seq  CATTTTCCACCAATGTCCCTCACATTGCACAATTTCTCAAACAAGGCTATTGTCTTAATTATAGTATTGCTGCATCTAACGTCTCGGTAGACTGCCTACGTACCCTAAACAAGGGTCAAGCCATTCGTAGTTCACATTAGTACTTCAAAGCATTCACAGT  478
ProMsMYB87.seq  CATTTTCCACCAATGTCCCTCACATTGCACAATTTCCCAAACAAGGCTATTGTCTCAATTATAGTATTGCTGCATCTAACGTCTCGGTAGACTGCCTACGTACCCTAAACAAAGGTCAAGCCATTCATAGTTTGCATTAGTACTTCAAAGCATTCACAGT  480
Consensus       cattttccaccaatgtccctcacattgcacaatttc caaacaaggctattgtct aattatagtattgctgcatctaacgtctcggtagactgcctacgtaccctaaacaa ggtcaagccattc tagtt  cattagtacttcaaagcattcacagt

ProMdMYB87.seq  AATTATATGCAATAATCAATTTATAACCGTTGGTGGAATTATCAATCATATATATATATATATATATATATACA............CGATAGAAAGGAAAAGACCCACTTACTTGAAGTCCGTGCTACGACTTGCATTGACCATTGTCTAGACATCACGA  626
ProMsMYB87.seq  AATTATATGCAATAATCAATTTATAACCTTTGGTGGAATTATCAATTATATATATATATATATAAATATATATATATTTATATATACGATAGAAAGGAAAAGACCCACTTACCTGAGGTCCGTGCTACAACTCCCTAGCACGAATATCAAGACATCTCGA  640
Consensus       aattatatgcaataatcaatttataacc ttggtggaattatcaat atatatatatatatata atatata a            cgatagaaaggaaaagacccacttac tga gtccgtgctac act  c    ac a t tc agacatc cga

ProMdMYB87.seq  ACCATCGTCGCCTGTGACCAAGTTCGACTTGCATTGACCATTGTCTAGACATCACGAACCATCGTCGCCTGTGACCAAGTTCAATTTGGCCGGAAAAGGCTCCAATTTC.CTCAAACCCACCGAAACCCTAAAATGGGTGAACTTCGATTCTCCTTCCTC  785
ProMsMYB87.seq  ACCATCGTCGCCTGTGACCAAGTTCAACTCCCTAGCACGAATATCAAGACATCTCGAACCATCGTCGCCTGTGACCAAGTTCAATTTGGCCGGAAAATGCTCCAATTTCACTCAAACCCACCGAAACCCTAAAATGGGTGAACTTCGATTCTCTTTCCTC  800
Consensus       accatcgtcgcctgtgaccaagttc act  c    ac a t tc agacatc cgaaccatcgtcgcctgtgaccaagttcaatttggccggaaaa gctccaatttc ctcaaacccaccgaaaccctaaaatgggtgaacttcgattctc ttcctc

ProMdMYB87.seq  GGTGTTCCAACGC...............GGTCTTGTTCCTGGGTCCAAGGGGAGTTAGTTAAGGGTGGTGGTGGGTGGTGAAACTCGTTGGATTGAATGAATCATCGTCGAAACCTCACGGGTCACCCGCACGATTTTACACGGTTTTTGGCCTAAAAAT  930
ProMsMYB87.seq  GGTGTTCCAACGCTTTTACAACCAATTGGGTCTTGTTCCTGGGTCCAAGGGGAGTTAGTTAAGGGTGGTGGTGGGTGGTGAAACTCGTCGGATTGAATGAATCATCGTCGAAACCTCACGGGTCACCCGCACGGTTTTACACGGTTTTTGGCGTAAAAAT  960
Consensus       ggtgttccaacgc               ggtcttgttcctgggtccaaggggagttagttaagggtggtggtgggtggtgaaactcgt ggattgaatgaatcatcgtcgaaacctcacgggtcacccgcacg ttttacacggtttttggc taaaaat

ProMdMYB87.seq  CTTCAAATTTAGATGGAGATAGAAGATGGGGTATTGTGGAATGGGTTGCAGGAGAGGGATGAGTGAAAATCACCTGAAAAATTGGTGGAAATGGTCGGAATCGAAACCGAGTTGCCATCGGTTTTGTCCACATGGATGGGGAAAAATTCCCCTTTTTCTC  1090
ProMsMYB87.seq  CTTCAAATTTAGATGGAGATAGAAGATGGGGTATTGTGGAATAGGCTGCATGAGAGGGATGGGTGAAAATCACCTGAAAATTTGGTGGAAATGGTCGGAATCGAAACCGAGTCGCCATCGATTTTGTCCACATGGATGGGGAAAAATTCCCCTTTT.CTC  1119
Consensus       cttcaaatttagatggagatagaagatggggtattgtggaat gg tgca gagagggatg gtgaaaatcacctgaaaa ttggtggaaatggtcggaatcgaaaccgagt gccatcg ttttgtccacatggatggggaaaaattcccctttt ctc

ProMdMYB87.seq  ATTGGGTGATGCCTTTTTTCCTCATTGGGTGATGCCTTTTTCCTTCTTTAACATTAATTAGTCAGTTTTCTCATAAGGTGGGAGTTTAACACTTTATAATCTAAAATATAAATAATCAATTTCAAACATTCGTAACTATACCGTTATAATCCGGACTCGC  1250
ProMsMYB87.seq  ATTGGGTGATGCCTTTTTTCCTCAATGGGTGATGCCTTTTTCCTTCTTTAACATTAATTAGTCAGTTTTCTCATAAGGTGGGAGTTTAACACTTTATAATCTAAAATATAAATAATCAATTTCAAACATTCGTAACTATACCGTTATAGTCCGGACTCGC  1279
Consensus       attgggtgatgccttttttcctca tgggtgatgcctttttccttctttaacattaattagtcagttttctcataaggtgggagtttaacactttataatctaaaatataaataatcaatttcaaacattcgtaactataccgttata tccggactcgc

ProMdMYB87.seq  AAATGACTTTCGTCCATGCGTTTGAACCAACGAGTACTACACAAATACGCTAAAAGAAAAAATCTTACGTTTCTCTAGACAATGGTCAATGCAAGTCCTAGACAATGGTCAATGCAAGTCAAAGTCCTTGCCTCTAGAGTATTTTCGTCAATTCCCTCAA  1410
ProMsMYB87.seq  AAACGGCTTTCGCTCATGCGTTTGAACCAACGAGTACTACACAGATACGCTAAAATAATAAGTCTTACGTTTCTCTAGACAATGGTCAATGCAAGTCCTAGACAATGGTCAATGCAAGTCAAAGTCCTTGCCTCTAGAGTATTTTCGTCAATTCCCTCAA  1439
Consensus       aaa g ctttcg  catgcgtttgaaccaacgagtactacaca atacgctaaaa aa aa tcttacgtttctctagacaatggtcaatgcaagtcctagacaatggtcaatgcaagtcaaagtccttgcctctagagtattttcgtcaattccctcaa

ProMdMYB87.seq  TTAAAATAAATAAAAACGTAAATTCAGGAACGGGTTATCACAATTTAGTGTCACGTAATAATATTTTTCCGGTTAAAACTTTTTGATGAGACAATATTTTAATTTACAGGAGTGAAACAAACTATATTAACAAAAAATCAAATATTAAATCTCTCACTT  1570
ProMsMYB87.seq  TTAAAATAAATAAAAACGTAAATTCAGGAACGGGTTATCACAATTTAGTGTCACGTAATAATATTTTTCCGGTTAAAACTTTTTGATGAGACAATATTTTAATTTACAGGAGTGAAACAAACTATATTAACAAAAAATCAAATATTAAATCTCTCACTT  1599
Consensus       ttaaaataaataaaaacgtaaattcaggaacgggttatcacaatttagtgtcacgtaataatatttttccggttaaaactttttgatgagacaatattttaatttacaggagtgaaacaaactatattaacaaaaaatcaaatattaaatctctcactt

ProMdMYB87.seq  ACAAATAAAAGAAAATATCGTAACACCAAATTTGAACAATAATTCATCTCCAAAAAAAGACGAAGTCAAATACACATCCGTTTGACTGGTCATGTCTCTTCATAAGCAAGAAACAAGAATCATACATAAAATATTACAATTAAAATTGAAAAATATGGAT  1730
ProMsMYB87.seq  ACAAATAAAAGAAAATATCGTAACACCAAATTTGAACAATAATTCATCTCCAAAAAAAGACGAAGTCAAATACACATCCGTTTGACTGGTCATGTCTCTTCATAAGCAAGAAACAAGAATCATACATAAAATATTACAATTAAAATTGAAAAATATGGAT  1759
Consensus       acaaataaaagaaaatatcgtaacaccaaatttgaacaataattcatctccaaaaaaagacgaagtcaaatacacatccgtttgactggtcatgtctcttcataagcaagaaacaagaatcatacataaaatattacaattaaaattgaaaaatatggat

ProMdMYB87.seq  ATCCGTACAGAAGTCAAGCATTTTATTTATTTAATTATTTCAAGGTGCCACCATCTTTATAAACCCCCTCCTCCACCAAAACCCCTTACATTTTGCGTGTCTGATTTTTCCTCTGTCGTCTCGGTCCCTTCTGGTTGGTTCTTGGGGAGTGAGTGAGGGA  1890
ProMsMYB87.seq  ATCCGTACAGAAGTCAAGCATTTTATTTATTTAATTATTTCAAGGTGCCACCATCTTTATAAACCCCCTCCTCCACCAAAACCCCTTACATTTTGCGTGTCTGATTTTTCCTCTGTCGTCTCGGTCCCTTCTGGTTGGTTCTTGGGGAGTGAGTGAGGGA  1919
Consensus       atccgtacagaagtcaagcattttatttatttaattatttcaaggtgccaccatctttataaaccccctcctccaccaaaaccccttacattttgcgtgtctgatttttcctctgtcgtctcggtcccttctggttggttcttggggagtgagtgaggga

ProMdMYB87.seq  ACACAGTAAGTAAC  1904
ProMsMYB87.seq  ACACAGTAAGTAAC  1933
Consensus       acacagtaagtaac
```
