# Supplementary material for: The MsSCL28.5–MdMYB87 module enhances drought tolerance by promoting adventitious root development in apple rootstocks
Source: Hortic Res. 2026 Jun 2;13(11):uhag211. doi: 10.1093/hr/uhag211 (PMC13718307; doi:10.1093/hr/uhag211)
Supplement: Supplementary file 1 [file web_material_uhag211.zip › Supplemental Figures and Tables.docx]

**Supplemental Figures:**

**Supplemental Figure S1.** Identification of *MsSCL28.5* overexpression apple plants and measurement of soil relative water content. (A) Relative transcript levels of *MsSCL28.5* in the roots of ‘M26’ and *MsSCL28.5*-OE apple plants analyzed by RT‑qPCR. (B) Soil relative water content during the drought treatment (at 0 d, 7 d, and 14 d). Data are represented as means ± SD (*n* = 3). Asterisks indicate significant differences between the *MsSCL28.5*-OE plants and ‘M26’ plants (**P* < 0.05, ***P* < 0.01; based on the one-way ANOVA with Tukey’s multiple comparisons test).

**Supplemental Figure S2.** Overexpression of *MsSCL28.5* enhances anti-oxidant enzymes activities in transgenic plants under drought stress. (A) Superoxide dismutase (SOD), (B) catalase (CAT), (C) peroxidase (POD), and (D) ascorbate peroxidase (APX) activity. (E) Superoxide (O_2_^-^) content, (F) hydrogen peroxide (H_2_O_2_) content of non-transgenic ‘M26’ and *MsSCL28.5*-overexpressing (OE) apple plants. Measurements were taken before and after a 14-day drought treatment. (G, H) Detection of O_2_^-^, H_2_O_2_ accumulation by nitroblue tetrazolium (NBT) staining and 3,3′-diaminobenzidine (DAB) staining, respectively, scale bars = 1 cm. All parameters were measured in leaf samples. Data represent means ± SD (*n* = 3). Asterisks indicate significant differences between the *MsSCL28.5*-OE plants and the non-transgenic ‘M26’ plants (**P* < 0.05, ***P* < 0.01; based on one‐way ANOVA with Tukey’s multiple comparisons test).

**Supplemental Figure S3.** Overexpression of *MsSCL28.5* enhances root growth of transgenic apple plants by increasing cell length. The longitudinal section of the root tip of ‘M26’ plants (A) and *MsSCL28.5*-OE plants (B). (C) Cell length of root elongation region. Data represent means ± SD (*n* = 50). Asterisks indicate significant differences between the *MsSCL28.5*-OE plants and the ‘M26’ plants (**P* < 0.05; based on one‐way ANOVA with Tukey’s multiple comparisons test).

**Supplemental Figure S4.** Overexpression of *MsSCL28.5* enhances the osmotic stress tolerance of ‘M26’. (A) Phenotypes of forty-day-old ‘M26’ and *MsSCL28.5*-OE apple plants under control or a 14-day 300 mM Mannitol treatment, scale bar = 1 cm. (B) Proline content, (C) MDA content in ‘M26’ and *MsSCL28.5*-OE apple plants under control or a 14-day 300 mM Mannitol treatment. (D) Detection of O_2_^-^ accumulation by NBT staining, scale bar = 1 cm. (E) Detection of H_2_O_2_ accumulation by DAB staining, scale bar=1 cm. (F) Quantitative measurement of O_2_^-^. (G) Quantitative measurement of H_2_O_2_. All parameters were measured in leaf samples. Data are represented as means ± SD (*n* = 3). Asterisks indicate significant differences between the *MsSCL28.5*-OE plants and ‘M26’ plants (**P* < 0.05, ***P* < 0.01; based on the one-way ANOVA with Tukey’s multiple comparisons test).

**Supplemental Figure S5.** *SCL28.5*-OE rootstock enhances the drought tolerance of wild type scion ‘GL-3’ by increasing antioxidant enzyme capacity. (A) SOD, (B) CAT, (C) POD, and (D) APX activity of GL-3/M26 and GL-3/OE-20 plants. (E) O_2_^-^, (F) H_2_O_2_ contents in the leaves of GL-3/M26 and GL-3/OE-20 plants. (G, H) Detection of O_2_^-^, H_2_O_2_ accumulation by NBT staining and DAB staining, respectively, scale bars = 1 cm. Measurements were taken before and after a 14-day drought treatment. All parameters were measured in leaf samples. Data represent means ± SD (*n* = 3). Asterisks indicate significant differences between the GL-3/OE-20 plants and GL-3/M26 plants (**P* < 0.05; based on Student’s *t*-test).

**Supplemental Figure S6.** Overexpression of *MsSCL28.5* alters the expression of *LBD* family genes in transgenic apple plants. (A) Heat map showing the relative mRNA levels of *LBDs* in ‘M26’ and *MsSCL28.5*-OE plant roots, expression of each *LBD* gene in non-transgenic ‘M26’ was normalized to 1. (B) Validation of expression pattern for eight selected *LBD* genes (*MdLBD4c*, *MdLBD7*, *MdLBD16a*, *MdLBD20b*, *MdLBD21a*, *MdLBD21b*, *MdLBD24c* and *MdLBD41b*) that were upregulated in the OE-plants. Data are represented as means ± SD (*n* = 3). Asterisks indicate significant differences between the *MsSCL28.5*-OE plants and ‘M26’ plants (**P* < 0.05, ***P* < 0.01; based on the one-way ANOVA with Tukey’s multiple comparisons test).

**Supplemental Figure S7.** Subcellular localization and yeast transcription activation analysis of MsSCL28.5. (A) Subcellular localization of MsSCL28.5*-*GFP fusion protein in *N. benthamiana* leaves, the mCherry fluorescent protein was used as nucleus marker, scale bars = 200 μm. (B) Transcription activation activity analysis of MsSCL28.5 in yeast. The full-length *MsSCL28.5* coding sequence was fused to the DNA-binding domain (BD) in the LexA vector. This construct and an empty activation domain (AD; pB42AD) vector were co-transformed into the reporter yeast strain EGY48. Growth and blue color development on selective medium containing X-Gal indicate transcriptional activation. MsDREB6.2 was used as a positive control.

**Supplemental Figure S8.** Multiple sequence alignment of MYB87 homologs**.** Protein sequences of MYB87 from *Arabidopsis thaliana*, apple (*Malus domestica*), pear (*Pyrus bretschneideri*), peach (*Prunus persica*), apricot (*Prunus armeniaca*), strawberry (*Fragaria ananassa*), and walnut (*Juglans regia*) were aligned. Conserved MYB DNA-binding domains are highlighted: the R2 domain is indicated by a red line, and the R3 domain by a blue line.

**Supplemental Figure S9.** Subcellular localization and yeast transcription activation analysis of MdMYB87. (A) Subcellular localization of MdMYB87*-*GFP fusion protein in *N. benthamiana* leaves, the mCherry fluorescent protein was used as nucleus marker, scale bars = 200 μm. (B) Transcription activation activity analysis of MdMYB87 in yeast. The full-length *MdMYB87* coding sequence was fused to the DNA-binding domain (BD) in the LexA vector. This construct and an empty activation domain (AD; pB42AD) vector were co-transformed into the reporter yeast strain EGY48. Growth and blue color development on selective medium containing X-Gal indicate transcriptional activation. MsDREB6.2 was used as a positive control.

**Supplemental Figure S10.** Alignment of *SCL28.5* coding sequences from cultivated and wild apple**.** The coding sequences of the *SCL28.5* from the cultivated rootstock ‘M26’ (*Malus domestica*) and wild apple (*Malus sieversii*) were aligned. The sequences are 100% identical, with no nucleotide variations detected.

**Supplemental Figure S11.** Alignment of *MYB87* coding sequences from cultivated and wild apple**.** The coding sequences of the *MYB87* from the cultivated rootstock ‘M26’ (*Malus domestica*) and wild apple (*Malus sieversii*) were aligned. A single nucleotide polymorphism (SNP) was identified between the two genotypes.

**Supplemental Figure S12.** Both MdMYB87 and MsMYB87 can interacts with MsSCL28.5 and exhibited equivalent synergistic effects with MsSCL28.5 in transactivating the *LBD16a* promoter. (A) Y2H assay showing that MsSCL28.5 interacts with MdMYB87 and MsMYB87, MsSCL28.5 was fused with the LexA DNA binding domain in pEG202 vector, and MdMYB87 and MsMYB87 were fused to the AD in pB42AD. LexA and AD served as the negative controls. (B, C) Dual-luciferase assay showing that co-transformation of MsSCL28.5 and MdMYB87 or MsSCL28.5 and MsMYB87 resulted in equivalent synergistic effects in transactivating the *MdLBD16a* expression in *N. benthamiana* leaves. Representative images of *N. benthamiana* leaves 72 h after infiltration are shown. The relative LUC activities normalized to the REN activity (LUC/REN) were quantified. Data are represented as means ± SD (*n* = 3).

**Supplemental Figure S13.** MdMYB87 enhanced the DNA-binding activity of MsSCL285. EMSA assay confirming that MdMYB87 enhanced the binding of MsSCL285 to the GAAAAT element in the *MdLBD16a* promoter.

**Supplemental Figure S14.** Identification of *MdMYB87* overexpression apple plants. Relative transcript levels of *MdMYB87* in the roots of ‘M26’ and *MdMYB87*-OE apple plants analyzed by RT‑qPCR. Data are represented as means ± SD (*n* = 3). Asterisks indicate significant differences between the *MdMYB87*-OE plants and ‘M26’ plants (***P* < 0.01; based on the one-way ANOVA with Tukey’s multiple comparisons test).

**Supplemental Figure S15.** MdMYB87 promotes AR development in transgenic apple plants by affecting the expression of *MdLBD16a* and *MdLBD21b*. Relative expression levels of *MdLBD16a* and *MdLBD21b* were analyzed by RT-qPCR in the roots of ‘M26’ and *MdMYB87*-OE plants. Data are represented as means ± SD (*n* = 3). Asterisks indicate significant differences between the *MdMYB87*-OE plants and ‘M26’ plants (**P* < 0.05; based on the one-way ANOVA with Tukey’s multiple comparisons test).

**Supplemental Figure S16.** Overexpression of *MdMYB87* enhances the drought tolerance of ‘M26’. (A) O_2_^-^ content in the leaves of ‘M26’ and *MdMYB87*-OE plants. (B) H_2_O_2_ content in the leaves of ‘M26’ and *MdMYB87*-OE plants. (C) SOD, (D) CAT, (E) POD, and (F) APX activity in the leaves of ‘M26’ and *MdMYB87*-OE plants. Measurements were taken before and after a 14-day drought treatment. Data are represented as means ± SD (*n* = 3). Asterisks indicate significant differences between the *MdMYB87*-OE plants and ‘M26’ plants (**P* < 0.05, ***P* < 0.01; based on the one-way ANOVA with Tukey’s multiple comparisons test).

**Supplemental Figure S17.** Alignment of the *SCL28.5* promoter sequences from cultivated and wild apple**.** Promoter sequences of the *SCL28.5* gene from the cultivated rootstock ‘M26’ (*Malus domestica*) and wild apple (*Malus sieversii*) were aligned to identify sequence variations.

**Supplemental Figure S18.** Prediction of *cis*-regulatory elements in the *SCL28.5* promoters. *Cis*-regulatory elements in the *SCL28.5* promoters from the cultivated rootstock ‘M26’ (*Malus domestica*) and wild apple (*Malus sieversii*) were predicted using the PlantCARE database.

**Supplemental Figure S19.** Promoter methylation level of *SCL28.5* in ‘M26’ was higher than that in *Malus sieversii*. McrBC-qPCR analysis of DNA methylation levels at the *SCL28.5* promoter, qPCR was performed using genomic DNA treated with or without McrBC. The relative levels of DNA retained after McrBC digestion were calculated using the 2^⁻ΔΔCt^ method with *MdActin7* serving as an internal control. Data are represented as means ± SD (*n* = 3). Asterisks indicate significant differences (**P* < 0.05, ***P* < 0.05; based on the one-way ANOVA with Tukey’s multiple comparisons test).

**Supplemental Figure S20.** Expression analysis of DNA methyltransferases and demethylases and effect of DNA methylation inhibitor on drought tolerance in apple rootstock. (A) Relative transcript levels of key DNA methylation pathway genes in *M. domestica* and *M. sieversii* under normal and PEG-induced drought stress conditions. Data are represented as means ± SD (*n* = 3). Asterisks indicate significant differences between the PEG 0 h and PEG 6 h (**P* < 0.05; based on Student’s *t*-test). (B) Phenotypes of two-month-old, hydroponically cultured ‘M26’ plants pre-treated for 10 days with either DMSO (control) or 5-aza-2′-deoxycytidine (5-AZA), followed by 6 hours of PEG treatment to induce osmotic stress, scale bars = 1 cm.

**Supplemental Figure S21.** Alignment of the *MYB87* promoter sequences from cultivated and wild apple**.** Promoter sequences of the *MYB87* gene from the cultivated rootstock ‘M26’ (*Malus domestica*) and wild apple (*Malus sieversii*) were aligned to identify sequence variations.

**Supplemental Figure S22.** Drought induced the expression of *TBP1b* in cultivated and wild apple rootstocks. Transcript levels of *TBP1b* were analyzed by RT-qPCR in the cultivated rootstock ‘M26’ (*Malus domestica*) and wild apple (*Malus sieversii*) under control conditions and after 8 h of treatment with 20% PEG-6000 to induce drought stress. Data are represented as means ± SD (*n* = 3). Asterisks indicate significant differences between the PEG 0 h and PEG 8 h (**P* < 0.05; based on Student’s *t*-test).

**Supplemental Figure S23.** Expression levels of *LBD16a* and *LBD21b* were higher in wild apple (*Malus sieversii*) under drought stress. Transcript levels of *LBD16a* and *LBD21b* in roots of the cultivated rootstock ‘M26’ (*Malus domestica*) and wild apple (*Malus sieversii*) were analyzed by RT‑qPCR following PEG‑induced drought stress. Data are represented as means ± SD (*n* = 3). Asterisks indicate significant differences between the *Malus sieversii* and ‘M26’ (**P* < 0.05; based on Student’s *t*-test).

**Supplemental Figure S24.** Drought stress suppresses the auxin biosynthesis in apple roots. Endogenous indole-3-acetic acid (IAA) content in the roots of four-month-old soil-grown ‘M26’ plants under well-watered (control) and drought (water withheld for 14 days) condition. Data are represented as means ± SD (*n* = 3). Asterisks indicate significant differences between the control and drrought (**P* < 0.05; based on Student’s *t*-test).

**Supplemental Tables**

**Supplemental Table S1 Primers**

**Reverse Transcription Quantitative PCR (RT-qPCR)**

| Gene name | Sequences (5′**→**3′) |
| --- | --- |
| MdActin7-F | GGCTGGATTTGCTGGTGATG |
| MdActin7-R | TGCTCACTATACCGTGCTCA |
| MdSCL28.5-F | GGCAGTAGGGTTCATTGGGTC |
| MdSCL28.5-R | TTGCAATCTGTCACCGTAAAGG |
| MdMYB87-F | GTCTGCGGTGGCTGAACTATCTG |
| MdMYB87-R | CATCGGCTTCCCATCTGGTTGTAG |
| MdLBD1a-F | ATCAACCTCATCATCACCATCAGTAC |
| MdLBD1a-R | TCCTCCTCCTCCTCCTCCATTC |
| MdLBD1b-F | AAGCAATGTCACCAAACTTCTAAACG |
| MdLBD1b-R | GCACCAACACAGCCATAGACC |
| MdLBD1c-F | CAATCTCCTCCTTCTTCTTCACCATC |
| MdLBD1c-R | CACTTGTCTGCACATCTTCTCCTC |
| MdLBD1d-F | AGGCACAGGCTGAGGTTGTC |
| MdLBD1d-R | CCGTGAGGAATGTTGGTGATGAAG |
| MdLBD1e-F | CTCCATCTTCCTCTCCTTCTTCTCC |
| MdLBD1e-R | GTGGTGGTGGTGGTGGTGAG |
| MdLBD1f-F | CTACAAATCCATTTCCGACCTCTCC |
| MdLBD1f-R | CGCACTTCTCCGCACACTTG |
| MdLBD1g-F | CTAATCCATTTCCGACCTATCCTTCAC |
| MdLBD1g-R | CCAAAGACTACACTTCTCCACACAC |
| MdLBD1h-F | GATGCTACGCCGAAGATGTGAC |
| MdLBD1h-R | TTCTTGTCTCTTCCACCATCTGAATC |
| MdLBD2a-F | AAGGTATAAGCAGCAGCAGAATCG |
| MdLBD2a-R | ACCATTCCAATTACCCACCAAATCC |
| MdLBD2b-F | GCAAGAACAGCAAAGAGCATTAGG |
| MdLBD2b-R | TGTGGTTGACGATGATGATAAGGTG |
| MdLBD2c-F | GTTGGGCAAGGACAGCAAAGAG |
| MdLBD2c-R | GCGGTTGTTGGTGGTGATAAGG |
| MdLBD4a-F | GAAGCAAATGCCAGGGTGAGAG |
| MdLBD4a-R | CTAGAGCCAGTTGGGTTTGAAGC |
| MdLBD4b-F | GAAGAAGGTGTGCTCAGGATTGTG |
| MdLBD4b-R | CATCTCCTCTCTGGTGCTCTGG |
| MdLBD4c-F | GATCCTGTGTACGGTTGTGTTGG |
| MdLBD4c-R | AGCAGTAGTAGTGGTAATAGCGAATTG |
| MdLBD6a-F | CTCTCTCCTCCAACACCAACTCC |
| MdLBD6a-R | GCGGCGGCGGCTATGAG |
| MdLBD6b-F | TGCCAGCCACGGACTCATAG |
| MdLBD6b-R | GAAGAGGACTGTAAATGTAGAGGATGG |
| MdLBD7-F | TTATGCCGATGGTTATGCCGATG |
| MdLBD7-R | ACATTGCCTTGTTCTTCTTGTTCTTG |
| MdLBD12a-F | TGCTCCTTTCTTTCCTTCAGATGAC |
| MdLBD12a-R | ATTTGCTTCATACACTAAACTGCTCAC |
| MdLBD12b-F | TCAATCACACTTTCTTCCTCTTCTCTC |
| MdLBD12b-R | CGGCGTCGCAGCAACTTG |
| MdLBD12c-F | GTAGCCTTACCAACCCAGATTGATC |
| MdLBD12c-R | CTGCTGAAGTATTGATGTGGAATGC |
| MdLBD12d-F | TCAACACCAACACCAACACCATC |
| MdLBD12d-R | GCTGCTGAAGTATTGATGTGGAATG |
| MdLBD13a-F | CGTCATCGTCATCGTCGTTTGG |
| MdLBD13a-R | GGCACTCTTCAGCACATCTTCTTC |
| MdLBD13b-F | GCCCTTTCTCTCCCTACTTCTCTC |
| MdLBD13b-R | CAGCCACCTCCATTAGCATCTTG |
| MdLBD15a-F | ATGAGGTAGGCAAGAAGTTTACAAGAG |
| MdLBD15a-R | GAGGAGTTTACAGGCAGCACAAG |
| MdLBD15b-F | AGGAAGTTGATGAGTTAGGCAAGAAG |
| MdLBD15b-R | GAGGAGTTTACAGGCAGCACAAG |
| MdLBD16a-F | TCCGAGTTATACGAGTTACATGAATCC |
| MdLBD16a-R | CTCCTCTCTGCTGCTTTGAATCTC |
| MdLBD16b-F | CCAAGTTATCCGAGCTACGTGAATC |
| MdLBD16b-R | CTCCTCTCTGCTGCTTTGAATCTC |
| MdLBD18-F | TCACATATTCGCTCTCCAACAACAG |
| MdLBD18-R | GGTAATGAGTGCTGCTGCTGATG |
| MdLBD19-F | CGCTGCCGCCACAATCAC |
| MdLBD19-R | CCATATCTGAGGACGATGACGATG |
| MdLBD20a-F | CACACCCTATTTCGGTTCAGTCC |
| MdLBD20a-R | CTTGAGCCTCGTAAGTGATGGTAAC |
| MdLBD20b-F | AACACCTCCGCCTCCACTAAC |
| MdLBD20b-R | TCTTCCTCATCATCTTCATCATCTTGG |
| MdLBD21a-F | TTGTCGTCGTGCTCTTCTTCTTG |
| MdLBD21a-R | GCTTGTCCATGCTGGCTGATC |
| MdLBD21b-F | AGTGTTCGGAGCAAGCAATGTG |
| MdLBD21b-R | AGCCGTAAACAGGGTCTCTCAG |
| MdLBD22a-F | CGCCAATAGCAACAACAATAGTGAC |
| MdLBD22a-R | CCATTCATCCCACCACCACATTG |
| MdLBD22b-F | CAACAAGAACAACAACAACAACAAGTG |
| MdLBD22b-R | AATGCTGATGTGGATATGGATGTGG |
| MdLBD24a-F | GCTCCAGCAACTACCAAACCATC |
| MdLBD24a-R | CTTGACACAACCGCAAACTGAATC |
| LBD24b-F | AAGACTCAGTGTACGGTTGTGTTAAG |
| MdLBD24b-R | TCCGCCGCCAGAGTTGATG |
| MdLBD24c-F | CCAGAGAGATTTGCTTGTGTTCATAAG |
| MdLBD24c-R | CATGGAGTCTGCTGCTGCTTC |
| MdLBD24d-F | TTTGCGAAGAAGATGCCCTCAAG |
| MdLBD24d-R | TTAAATGGACTGGAAGTTGCTCTAGC |
| MdLBD25-F | GAAGGAGTTGGACGAGACTAATGC |
| MdLBD25-R | CCACGAACCCTACTACTACTAGACC |
| MdLBD27a-F | CTCGTGTCCTTCATCGCAGTTG |
| MdLBD27a-R | GCCACCAGAAGCATATTGATACGG |
| MdLBD27b-F | CCAATTACAGCAAGCCCTAGAAGAG |
| MdLBD27b-R | AGAGTAGTGCGGTGAGGAAGAAG |
| MdLBD27c-F | AGCAGTCCCTATCACCTTGTATCTC |
| MdLBD27c-R | CTCCTCCTTTCGCTGGCATTG |
| MdLBD27d-F | AGCAGTCCCTATCACCTTGTATCTC |
| MdLBD27d-R | CTCCTCCTTTCGCTGGCATTG |
| MdLBD27e-F | GGGTGCTGGGAGGTGATCTG |
| MdLBD27e-R | GTTGCTGGTGGTGCTGTCTG |
| MdLBD27f-F | TTTACGGGTGCTGGGAGGTG |
| MdLBD27f-R | TGGTGGCGGTGCTGTCTG |
| MdLBD29a-F | AAGATCCTATTTATGGCTGTGTTTCCC |
| MdLBD29a-R | TTAGCTGCTTGTTCCTTGACTGAAG |
| MdLBD29b-F | GGTTCTGGTTCTCCTTGTGGTG |
| MdLBD29b-R | GATGGTTGCGAAGTGGGTAGC |
| MdLBD29c-F | TTCTGCCATGAACAAGGTGCTAC |
| MdLBD29c-R | GGCGGCTTCACAACGATCAC |
| LBD29d-F | TTCTGCCATGAACAAGGTGCTAC |
| MdLBD29d-R | GCCGCTTCACAACGATCACTC |
| MdLBD33a-F | GTTCGCTCCTTACTTCTGCTATGAC |
| MdLBD33a-R | CGCTTCGTAAGACATGGTGATGG |
| MdLBD33b-F | CGAATGCGTGATCCTGTGTATGG |
| MdLBD33b-R | ATGTGTAAGTGAGAAGTTTGGAGTAGG |
| MdLBD53-F | TGGCTGCGTCGGACTCATC |
| MdLBD53-R | GGCGGCTGGAGAATGGAGAG |
| MdLBD36b-F | CCTGGCTCGGTCACTGTGG |
| MdLBD36b-R | GGCTGCTGCTGCTGAATCTG |
| MdLBD36c-F | GGTGTGGTGTCTGGTTCATTGG |
| MdLBD36c-R | CGGTTGTGGTTGGAGCATCAG |
| MdLBD36d-F | CGGCAAGATCCTGAACGAACTC |
| MdLBD36d-R | GAGATGAAACCAACACAACCATACAC |
| MdLBD37a-F | CGGCGGCGTCATCAACTG |
| MdLBD37a-R | CTTGCGTTTCGGCGAGGAG |
| MdLBD37b-F | TGCCAGGCTGCCGTTGAG |
| MdLBD37bR | AGTGACGCCGCCGACATC |
| MdLBD37c-F | GAGAGAGGAAGAGGGAAGGGAATG |
| MdLBD37c-R | CTCGTCGTTGTCGTAGTCATCAC |
| MdLBD37d-F | CCTGTTCCTCGCCAAGTTCTTC |
| MdLBD37d-R | CCCACCGCTCCGTTCACC |
| MdLBD37e-F | TTGGATCGGATGGTGGAGATGAG |
| MdLBD37e-R | CCTTGGGCTTCGGGAGAGTC |
| MdLBD37f-F | TCTCCTTCATCAACGCCGTCTC |
| MdLBD37f-R | CACACCTCCCACTTCCCACTC |
| MdLBD37g-F | AATGCCTACAATGGATGGAAGACC |
| MdLBD37g-R | GACTGCGTTGATGAAGGAGAAGAG |
| MdLBD41a-F | CCCGAGCCGAGCCACAATC |
| MdLBD41a-R | TTCAGCCTCAATTCTTCTCTTCTTCAC |
| MdLBD41b-F | AAGCGGACGGAGTCTGTTCAG |
| MdLBD41b-R | AATCCAGCCTCAATTCTTCTCTTCTTC |
| MdLBD42-F | TACACCTCCAATCTCCTCCATTCAC |
| MdLBD42-R | AGGGTCTGATCTCGCATTCGTC |
| MdTBP1b-F | ACTGCTGGAGACCTTTGCTC |
| MdTBP1b-R | ACCCTTGTTTTGTGGGCTCA |
| MdCMT2-F | ACCGTTTGCGAGGTTATGGT |
| MdCMT2-R | AAAGCTCGTGCAACCGAAAC |
| MdROS1-F | GCAAGCCAGATATCTGACGC |
| MdROS1-R | TGGTTGGAATTTCGTCAGGACCT |
| MdROS2-F | AACTATGCCGACACAGAA |
| MdROS2-R | GCCACCAATTCAACTTCAT |
| MdDNMT2-F | GTGCTCTGTGAACTTGATAA |
| MdDNMT2-R | TGCCGCTACAATATCCAT |
| MdDRM2-F | AAGAAGAAGAAGAGGCAGAT |
| MdDRM2-F | GGAGAGTCCTATGTGTCAG |
| MdDRM3b-F | CAGGTCGTCCAGGCAGATAGCTGAA |
| MdDRM3b-R | TCTCTTCCATCCATGTTGGGTCAAGG |
| MdMET1a-F | GAGGATGTAGAAGAGGATGAA |
| MdMET1a-R | TGAAGACTGGCACACTTAT |
| MdDME1-F | ACAAGAATCGGACATCCA |
| MdDME1-R | CATTCAAGTCTATACCACCATC |
| MdMET1b-F | CTCATTCGCTGCCAGAGGAT |
| MdMET1b-R | CAGGCACCACGGGATTAAGT |
| MdCMT3-F | CCGATGGAAGATGAGGAGGC |
| MdCMT3-R | TCCTTGCACATGGGCATCAT |
| MdDRM3a-F | AAAGCTCCTCAGAACGGGAA |
| MdDRM3a-R | CTTTGTCCACGAGTGATGGTGAA |

**Construction of Overexpression Vectors**

| Gene Name | Sequences (5′**→**3′) |
| --- | --- |
| MsSCL28.5-pRI101-F | CACTGTTGATACATATGCCCGTCGAC ATGGCACTGAGCCGAAACTTC |
| MsSCL28.5-pRI101-R | CCCTTGCTCACCATGGATCCGGTACC GCATTTCCAGCAGGAGGCA |
| MdMYB87-pRI101-F | CACTGTTGATACATATGCCCGTCGAC ATGGGACGCACTCCATGCTGTG |
| MdMYB87-pRI101-R | CCCTTGCTCACCATGGATCCGGTACC AAAGAAATCACCAAAATCTG |

**Yeast One-Hybrid (Y1H) and Yeast Two-Hybrid (Y2H)**

| Gene Name | Sequences (5′**→**3′) |
| --- | --- |
| MsSCL28.5-pB42AD-F | GCCAGATTATGCCTCTCCCGAATTCATGGCACTGAGCCGAAACTT |
| MsSCL28.5-pB42AD-R | GCGAAGAAGTCCAAAGCTTCTCGAGGCATTTCCAGCAGGAGGCA |
| MdMYB87-pB42AD-F | GCCAGATTATGCCTCTCCCGAATTC ATGGGACGCACTCCATGCTGTG |
| MdMYB87-pB42AD-R | GCGAAGAAGTCCAAAGCTTCTCGAG AAAGAAATCACCAAAATCTG |
| MdTBP1a-pB42AD F | GCCAGATTATGCCTCTCCCGAATTC ATGGAAGAACAAGGTGGATCTG |
| MdTBP1a-pB42AD-R | GCGAAGAAGTCCAAAGCTTCTCGAG CTGGAAACAGTGTTGCTGGACC |
| MdTBP1b-pB42AD-F | GCCAGATTATGCCTCTCCCGAATTC ATGGGGGATCAAGGTGGATTAG |
| MdTBP1b-pB42AD-R | GCGAAGAAGTCCAAAGCTTCTCGAG CCAGCAGCAGCAGGAGGCAT |
| MdTBP8a-pB42AD-F | GCCAGATTATGCCTCTCCCGAATTC ATGAGCCATGGAGATGCGGAC |
| MdTBP8a-pB42AD-R | GCGAAGAAGTCCAAAGCTTCTCGAG CAACTGAGTGAGTTCCTGTGGG |
| MdTBP8c-pB42AD-F | GCCAGATTATGCCTCTCCCGAATTC ATGACCGATGGGGGTGTGGAG |
| MdTBP8c-pB42AD-R | GCGAAGAAGTCCAAAGCTTCTCGAG TAACTCAGCAAGCTCCTCTGGG |
| MdTBP10-pB42AD-F | GCCAGATTATGCCTCTCCCGAATTCATGAACCAGAACCCAACTCAAAAC |
| MdTBP10-pB42AD-R | GCGAAGAAGTCCAAAGCTTCTCGAG CTCTTCCCTTGTTGCAGGATCC |
| MdTBP11-pB42AD-F | GCCAGATTATGCCTCTCCCGAATTC ATGAAGCAGCAATCCAAGGACC |
| MdTBP11-pB42AD-R | CGAAGAAGTCCAAAGCTTCTCGAG CCGGAAGAGCCTAGACTGTGATC |
| MdTBP12b-pB42AD-F | GCCAGATTATGCCTCTCCCGAATTC ATGGCGGAAAACGGCCCATCG |
| MdTBP12b-pB42AD-R | GCGAAGAAGTCCAAAGCTTCTCGAG AAACCGAGTCATTTGCTGCATC |
| MdTBP13-pB42AD-F | GCCAGATTATGCCTCTCCCGAATTC ATGAGTAACCCTTCTGGAGGG |
| MdTBP13-pB42AD-R | GCGAAGAAGTCCAAAGCTTCTCGAG CTCCGTTGACCCGACTTTATC |
| MdLBD4cPro-lacz2u-F | ACCCGGGGATCTGTCGACCTCGAG GAGGTTCCTCCCATAGGAACGG |
| MdLBD4cPro-lacz2u-R | TATACATACAGAGCACATGCCTCGAG CACAATCCTGAGCACATCTCC |
| MdLBD7Pro-lacz2u-F | CCCGGGGATCTGTCGACCTCGAG GGGAGTTAGTGAGGAGTCCAAGGG |
| MdLBD7Pro-lacz2u-R | TATACATACAGAGCACATGCCTCGAG GCGGTTGGAGGGGAAGTAGG |
| MdLBD16aPro-lacz2u-F | ACCCGGGGATCTGTCGACCTCGAG TGGGTTCTTCTCGTGTGACCTG |
| MdLBD16aPro-lacz2u-R | TATACATACAGAGCACATGCCTCGAG GGAGATCCAGTGCCGATGCCA |
| MdLBD20bPro-lacz2u-F | ACCCGGGGATCTGTCGACCTCGAG CACGGGTTAAGCAGACGTGC |
| MdLBD20bPro-lacz2u-R | TATACATACAGAGCACATGCCTCGAG CATAGAAGCACGCTTTCCTCCC |
| MdLBD21aPro-lacz2u-F | ACCCGGGGATCTGTCGACCTCGAG GAGCGCTTCGTTTACAGTCC |
| MdLBD21aPro-lacz2u-R | TATACATACAGAGCACATGCCTCGAG CTTGCTCACATTGCTCGCCC |
| MdLBD21bPro-lacz2u-F | ACCCGGGGATCTGTCGACCTCGAG CCGTCCAAAGATGAACGTGAAG |
| MdLBD21bPro-lacz2u-R | TATACATACAGAGCACATGCCTCGAG CACTTTGTGAACTTGGGCG |
| MdLBD24cPro-lacz2u-F | ACCCGGGGATCTGTCGACCTCGAG GGGATAGAGGGTGAGCGACTG |
| MdLBD24cPro-lacz2u-R | TATACATACAGAGCACATGCCTCGAG GGGGGCCAAAACACAATCTTG |
| MdLBD41bPro-lacz2u-F | ACCCGGGGATCTGTCGACCTCGAG CTGTGAGTCATTTTGGTTCAGCTC |
| MdLBD41bPro-lacz2u-R | TATACATACAGAGCACATGCCTCGAG AACTTGGCGAGGAAGACGGTG |
| MYB87pro-lacz2u-F | ACCCGGGGATCTGTCGACCTCGAG ATGGACATACCCTAATGCAATCC |
| MYB87pro-lacz2u-R | TATACATACAGAGCACATGCCTCGAG CTTGAGAGCTGCGTCTTCTTCC |
| MdMYB87-pEG202-F | CGACTGGCTGGAATTC ATGGGACGCACTCCATGCTGTG |
| MdMYB87-pEG202-R | TGCAGGTCGACTCGAG AAAGAAATCACCAAAATCTG |

**Luciferase Complementation Imaging (LCI) assay**

| Gene Name | Sequences (5′**→**3′) |
| --- | --- |
| MsSCL28.5-Nluc-F | AACACGGGGGACGAGCTCGGTACCC ATGGCACTGAGCCGAAACTT |
| MsSCL28.5-Nluc-R | CGGGACGCGTACGAGATCTGGTCGAC GCATTTCCAGCAGGAGGCA |
| MdMYB87-Nluc-F | TCTCGTACGCGTCCCGGGGCGGTACC ATGGGACGCACTCCATGCTGTG |
| MdMYB87-Nluc-R | TACGAACGAAAGCTCTGCAGGTCGAC TCAAAAGAAATCACCAAAATCTG |

**Dual-luciferase assay**

| Gene Name | Sequences (5′**→**3′) |
| --- | --- |
| MsSCL28.5-62sk-F | GTGGATCCCCCGGGCTGCAG GAATTC ATGGCACTGAGCCGAAACTTC |
| MsSCL28.5-62sk-R | GAATTGGTACCGGGCCCCCC CTCGAG GCATTTCCAGCAGGAGGCA |
| MdMYB87-62sk-F | GTGGATCCCCCGGGCTGCAG GAATTC ATGGGACGCACTCCATGCTGTG |
| MdMYB87-62sk-R | GAATTGGTACCGGGCCCCCCCTCGAGAAAGAAATCACCAAAATCTGTGC |
| MdTBP1b-62sk-F | GTGGATCCCCCGGGCTGCAG GAATTC ATGGGGGATCAAGGTGGATTAG |
| MdTBP1b-62sk-R | GAATTGGTACCGGGCCCCCC CTCGAG CCAGCAGCAGCAGGAGGCAT |
| MdLBD16a pro-08luc-F | ACTCACTATAGGGCGAATTG GGTACC TGGGTTCTTCTCGTGTGACCTG |
| MdLBD16a pro-08luc-R | AGCTTATCGATACCGTCGAC CTCGAG GGAGATCCAGTGCCGATGCCA |
| MdLBD21b pro-08luc-F | ACTCACTATAGGGCGAATTG GGTACC CCGTCCAAAGATGAACGTGAAG |
| MdLBD21b pro-08luc-R | AGCTTATCGATACCGTCGAC CTCGAG CACTTTGTGAACTTGGGCG |
| MYB87 proP1-08luc-F | ACTCACTATAGGGCGAATTG GGTACC ATGGACATACCCTAATGCAATCC |
| MYB87 proP1-08luc-R | AGCTTATCGATACCGTCGAC CTCGAG GAACTTGGTCACAGGCGACGA |

**Electrophoretic Mobility Shift Assay (EMSA) and *In situ* Hybridization Assay**

| Gene Name | Sequences (5′**→**3′) |
| --- | --- |
| MsSCL28.5+PGEX4T-F | TCCGCGTGGATCCCCGGAATTC ATGGCACTGAGCCGAAACTT |
| MsSCL28.5+PGEX4T-R | CGATGCGGCCGCTCGAGTCGACC GCATTTCCAGCAGGAGGCA |
| MsSCL28.5+PET32a-F | GCCATGGCTGATATCGGATCC ATGGCACTGAGCCGAAACTT |
| MsSCL28.5+PET32a-R | TGCGGCCGCAAGCTTGTCGAC GCATTTCCAGCAGGAGGCA |
| MdMYB87-PGEX4T-F | TCCGCGTGGATCCCCGGAATTC ATGGGACGCACTCCATGCTGTG |
| MdMYB87-PGEX4T-R | CGATGCGGCCGCTCGAGTCGACC AAAGAAATCACCAAAATCTG |
| MdMYB87+PET32a-F | GCCATGGCTGATATCGGATCC ATGGGACGCACTCCATGCTGTG |
| MdMYB87+PET32a-R | TGCGGCCGCAAGCTTGTCGAC AAAGAAATCACCAAAATCTG |
| MdTBP1b-PGEX4T-F | TCCGCGTGGATCCCCGGAATTC ATGGGGGATCAAGGTGGATTAG |
| MdTBP1b-PGEX4T-R | CGATGCGGCCGCTCGAGTCGACC CCAGCAGCAGCAGGAGGCAT |
| MdTBP1b-PET32a-F | GCCATGGCTGATATCGGATCC ATGGGACGCACTCCATGCTGTG |
| MdTBP1b-PET32a-R | TGCGGCCGCAAGCTTGTCGAC AAAGAAATCACCAAAATCTG |
| MdLBD16a pro-28.5-Biotin-F | CGTGTGCTTAATTTTCATTATATTTTCTTTTTCTC |
| MdLBD16a pro-28.5-Cold-F | CGTGTGCTTAATTTTCATTATATTTTCTTTTTCTC |
| MdLBD16a pro-28.5-Cold-R | GAGAAAAAGAAAATATAATGAAAATTAAGCACACG |
| MdLBD16a pro-28.5-Mutant-F | CGTGTGCTTACCCGGGATTATCCCGGGTTTTTCTC |
| MdLBD16a pro-28.5-Mutant-R | GAGAAAAACCCGGGATAATCCCGGGTAAGCACACG |
| MdLBD21b pro-28.5-Biotin-F | AGAAATTAAATTTTATTTTCTAATTAGCCATGAAG |
| MdLBD21b pro-28.5-Cold-F | AGAAATTAAATTTTATTTTCTAATTAGCCATGAAG |
| MdLBD21b pro-28.5-Cold-R | CTTCATGGCTAATTAGAAAATAAAATTTAATTTCT |
| MdLBD21b pro-28.5-Mutant-F | AGAAATTAAATTTTCCCGGGTAATTAGCCATGAAG |
| MdLBD21b pro-28.5-Mutant-R | CTTCATGGCTAATTACCCGGGAAAATTTAATTTCT |
| MdLBD16a pro-87-Biotin-F | CCACAGGAGCACCTGTTGTATTATTCATCC |
| MdLBD16a pro-87-Cold-F | CCACAGGAGCACCTGTTGTATTATTCATCC |
| MdLBD16a pro-87-Cold-R | GGATGAATAATACAACAGGTGCTCCTGTGG |
| MdLBD16a pro-87-Mutant-F | CCACAGGAGCACAAAAAATATTATTCATCC |
| MdLBD16a pro-87-Mutant-R | GGATGAATAATATTTTTTGTGCTCCTGTGG |
| MdLBD21b pro-87-Biotin-F | CCATAACCGCAATAACCATTTCAAATTTTG |
| MdLBD21b pro-87-Cold-F | CCATAACCGCAATAACCATTTCAAATTTTG |
| MdLBD21b pro-87-Cold-R | CAAAATTTGAAATGGTTATTGCGGTTATGG |
| MdLBD21b pro-87-Mutant-F | CCATAACCGCAAAAAAAATTTCAAATTTTG |
| MdLBD21b pro-87-Mutant-R | CAAAATTTGAAATTTTTTTTGCGGTTATGG |
| MsMYB87 pro-TBP1b-Biotin-F | TATATATATATATATATAAATATATATATATTTATATATACGATAGA |
| MsMYB87 pro-TBP1b-Cold-F | TATATATATATATATATAAATATATATATATTTATATATACGATAGA |
| MsMYB87 pro-TBP1b-Cold-R | TCTATCGTATATATAAATATATATATATTTATATATATATATATATA |
| MdMYB87 pro-TBP1b-Biotin-F | CATATATATATATATATATATATATACACGATAGA |
| MdMYB87 pro-TBP1b-Biotin-R | TCTATCGTGTATATATATATATATATATATATATG |
| Antisense oligonucleotide probe for *in situ* hybridization | CTATTGACAACAAGGACTTCACCATCATACATTT |

**GUS Reporter Assay**

| Gene Name | Sequences (5′**→**3′) |
| --- | --- |
| MYB87 pro-1301-F | ACAGCTATGACCATGATTACGAATTC ATGGACATACCCTAATGCAATCC |
| MYB87 pro-1301-R | TTACCCTCAGATCTACCATGG CTTGAGAGCTGCGTCTTCTTCC |
| MYB87 proP1-1301-F | ACAGCTATGACCATGATTACGAATTC ATGGACATACCCTAATGCAATCC |
| MYB87 proP1-1301-R | TTACCCTCAGATCTACCATGG GAACTTGGTCACAGGCGACGA |
| MYB87 proP2-1301-F | ACAGCTATGACCATGATTACGAATTC CTCCCTAGCACGAATATCAAG |
| MYB87 proP2-1301-R | TTACCCTCAGATCTACCATGG GACTTGCATTGACCATTGTCTAG |
| MYB87 proP3-1301-F | ACAGCTATGACCATGATTACGAATTC CTAGACAATGGTCAATGCAAGTC |
| MYB87 proP3-1301-R | TTACCCTCAGATCTACCATGG CTTGAGAGCTGCGTCTTCTTCC |

**Bisulfite Sequencing (BSP)**

| Gene Name | Sequences (5′**→**3′) |
| --- | --- |
| SCL28.5 pro-BSP-F | ATGGAATTTAGATTGATGTATTTGAAAT |
| SCL28.5 pro-BSP-R | CTAAACACATCAATAATAACCTTAACTT |

**McrBC-qPCR**

| Gene Name | Sequences (5′**→**3′) |
| --- | --- |
| SCL28.5 pro-McrBC-F | GAGGAACATCCCTCTTATATTG |
| SCL28.5 pro-McrBC-R | CCACCTGTATAATTTAGTAGG |

**ChIP-qPCR**

| Gene Name | Sequences (5′**→**3′) |
| --- | --- |
| ChIP-SCL28.5-LBD16a-F | CTAAATGCCCCACTTTCCTCCTT |
| ChIP-SCL28.5-LBD16a-R | CATTTTTGGTACAGACAAACAC |
| ChIP-MYB87-LBD16a-F | ATGTGCCCTAAGGAGGTGGC |
| ChIP-MYB87-LBD16a-R | CGTGGTCAATCAAGTATATGAC |
| ChIP-SCL28.5-LBD21b-F | ATGCATGTGATGGAAAGG |
| ChIP-SCL28.5-LBD21b-R | GGTGGGAGTGCATCTTCTCTT |
| ChIP-MYB87-LBD21b-F | CAAACATTCTTTGTGCACGATG |
| ChIP-MYB87-LBD21b-R | CCTTTCCATCACATGCAT |

**Supplemental Table S2 Accession numbers**

| Gene | Accession No. |
| --- | --- |
| MdActin7 | [XM_070822516.1](https://www.ncbi.nlm.nih.gov/nucleotide/XM_070822516.1?report=genbank&log$=nuclalign&blast_rank=4&RID=PR4DVAHE016) |
| MsSCL28.5 | MW308139.1 |
| MdMYB87 | MD08G1074700 |
| MdLBD1a | MD14G1174300 |
| MdLBD1b | MD06G1169000 |
| MdLBD1c | MD11G1125200 |
| MdLBD1d | MD03G1111600 |
| MdLBD1e | MD07G1178900 |
| MdLBD1f | MD01G1130900 |
| MdLBD1g | MD01G1130800 |
| MdLBD1h | MD07G1152600 |
| MdLBD2a | MD07G1305100 |
| MdLBD2b | MD10G1286400 |
| MdLBD2c | MD05G1307000 |
| MdLBD4a | MD10G1238700 |
| MdLBD4b | MD05G1259100 |
| MdLBD4c | MD05G1222100 |
| MdLBD6a | MD00G1016900 |
| MdLBD6b | MD12G1261100 |
| MdLBD7 | MD15G1058700 |
| MdLBD12a | MD01G1157900 |
| MdLBD12b | MD07G1226600 |
| MdLBD12c | MD03G1060100 |
| MdLBD12d | MD11G1063200 |
| MdLBD13a | MD03G1059400 |
| MdLBD13b | MD11G1061600 |
| MdLBD15a | MD01G1162900 |
| MdLBD15b | MD07G1231000 |
| MdLBD16a | MD10G1187700 |
| MdLBD16b | MD05G1200900 |
| MdLBD18 | MD02G1202500 |
| MdLBD19 | MD02G1203000 |
| MdLBD20a | MD11G1229500 |
| MdLBD20b | MD14G1006900 |
| MdLBD21a | MD07G1224700 |
| MdLBD21b | MD01G1155900 |
| MdLBD22a | MD00G1059900 |
| MdLBD22b | MD09G1192800 |
| MdLBD24a | MD07G1263500 |
| MdLBD24b | MD01G1196600 |
| MdLBD24c | MD09G1160200 |
| MdLBD24d | MD17G1146000 |
| MdLBD25 | MD17G1029300 |
| LBD27a | MD09G1045500 |
| MdLBD27b | MD17G1046200 |
| MdLBD27c | MD07G1060400 |
| MdLBD27d | MD07G1060600 |
| MdLBD27e | MD14G1191900 |
| MdLBD27f | MD06G1185700 |
| MdLBD29a | MD05G1200700 |
| MdLBD29b | MD10G1187300 |
| MdLBD29c | MD05G1200800 |
| MdLBD29d | MD10G1187600 |
| MdLBD33a | MD07G1202600 |
| MdLBD33b | MD01G1138100 |
| MdLBD36a | MD02G1166900 |
| MdLBD36b | MD15G1279500 |
| MdLBD36c | MD05G1277500 |
| MdLBD36d | MD10G1256700 |
| MdLBD37a | MD00G1015800 |
| MdLBD37b | MD15G1294700 |
| MdLBD37c | MD08G1086800 |
| MdLBD37d | MD15G1071500 |
| MdLBD37e | MD02G1165000 |
| MdLBD37f | MD05G1097900 |
| MdLBD37g | MD10G1103500 |
| MdLBD41a | MD09G1088700 |
| MdLBD41b | MD17G1078600 |
| MdLBD42 | MD16G1042900 |
| MdTBP1a | MD12G1176800 |
| MdTBP1b | MD14G1002000 |
| MdTBP8a | MD12G1113700 |
| MdTBP8c | MD09G1068300 |
| MdTBP810 | MD08G1049200 |
| MdTBP11 | MD10G1223700 |
| MdTBP12b | MD09G1184100 |
| MdTBP13 | MD02G1287100 |
| MdCMT2 | MD06G1022700 |
| MdCMT3 | MD11G1012800 |
| MdMET1a | MD02G1115100 |
| MdMET1b | MD15G1231100 |
| MdDME1 | MD11G1157400 |
| MdROS1 | MD12G1017600 |
| MdROS2 | MD14G1015300 |
| MdDRM2 | MD17G1031900 |
| MdDNMT2a | MD15G1120500 |
| MdDRM3a | MD16G1140200 |
| MdDRM3b | MD13G1146100 |
